# From Natriuretic Peptides to microRNAs: Multi-Analyte Liquid Biopsy Horizons in Heart Failure

**DOI:** 10.3390/biom15081189

**Published:** 2025-08-19

**Authors:** Evelina Charidemou, Kyriacos Felekkis, Christos Papaneophytou

**Affiliations:** 1 Department of Life Sciences, School of Life and Health Sciences, University of Nicosia, 2417 Nicosia, Cyprus; charidemou.e@unic.ac.cy (E.C.); felekkis.k@unic.ac.cy (K.F.); 2 Non-Coding RNA Research Laboratory, School of Life and Health Sciences, University of Nicosia, 2417 Nicosia, Cyprus

**Keywords:** circulating microRNAs, heart failure, multianalyte liquid biopsy, stratification, natriuretic peptides

## Abstract

Heart failure (HF) is a leading cause of morbidity and mortality worldwide, underscoring the need for improved diagnostic, prognostic, and therapeutic strategies. Circulating microRNAs (c-miRNAs) have emerged as promising non-invasive biomarkers due to their stability, tissue specificity, and regulatory roles in cardiac pathophysiology. This review highlights the potential of c-miRNAs in enhancing HF diagnosis, risk stratification, and therapeutic monitoring, particularly when integrated with conventional biomarkers such as natriuretic peptides, galectin-3, soluble ST2, and high-sensitivity troponins. We explore the roles of key miRNAs in HF pathogenesis—including cardiac hypertrophy, fibrosis, inflammation, apoptosis, and vascular remodeling—and discuss their diagnostic and prognostic significance. The potential of multi-analyte liquid biopsy approaches that combine c-miRNAs with protein biomarkers is also examined within the context of precision medicine. Despite promising data, challenges related to standardization, assay variability, and clinical validation remain. Addressing these gaps through harmonized protocols and large-scale studies will be essential for translating c-miRNAs into routine HF management.

## 1. Introduction

Heart failure (HF) is a major global health problem and one of the leading causes of hospitalization and mortality worldwide [1]. In Western countries, the annual incidence ranges from 1 to 9 cases per 1000 individuals [2], with a prevalence of approximately 1–2% in the adult population [3]. Currently, more than 64 million people are affected by HF globally [4].

In 2021, a comprehensive universal definition and classification system for HF was established, characterizing HF as a clinical syndrome manifesting through symptoms and/or signs attributable to structural and/or functional cardiac abnormalities, with confirmation provided by elevated natriuretic peptide (NP) concentrations and/or objective evidence of pulmonary or systemic congestion [5]. This framework introduced a revised four-stage classification system: stage A (at-risk for HF), stage B (pre-HF), stage C (symptomatic HF), and stage D (advanced HF). The classification system based on ejection fraction (EF) parameters was simultaneously updated to include three primary categories: HF with reduced ejection fraction (HFrEF; EF ≤ 40%), HF with mildly reduced ejection fraction (HFmrEF; EF 41– 49%), and HF with preserved ejection fraction (HFpEF; EF ≥ 50%) [6]. A novel classification, HF with improved ejection fraction (HFimpEF), was introduced to address the dynamic nature of HF pathophysiology. This category is defined by baseline left ventricular ejection fraction (LVEF) ≤ 40%, a minimum 10-point improvement from baseline LVEF, and subsequent LVEF measurement >40%, thereby capturing the clinical reality of treatment-responsive HF phenotypes [7]. In addition, according to the new 2023 European Society of Cardiology (ESC) guidelines, HF is defined as a clinical syndrome characterized by typical symptoms and/or signs that result from structural and/or functional abnormalities of the heart, leading to elevated intracardiac pressures and/or reduced cardiac output at rest or during exertion [3].

HF commonly arises from a range of cardiovascular conditions, including ischemic heart disease [8], hypertension [9], valvular abnormalities [10], and cardiomyopathies [10]. It is characterized by the heart’s inability to pump sufficient oxygenated blood to meet the body’s metabolic demands, resulting in clinical manifestations such as fatigue, dyspnea, fluid retention, and reduced exercise tolerance [11]. Depending on the underlying pathophysiology, HF may present with impaired systolic function, diastolic dysfunction, or both, underscoring the need for precise diagnosis and phenotyping [12]. HF can present in either acute (AHF) or chronic (CHF) forms and typically follows a progressive trajectory if not diagnosed and managed in a timely manner. Notably, HF and atrial fibrillation (AF) may develop independently of coronary artery disease (CAD), with hypertension and congenital heart defects also contributing to their pathogenesis [13].

Clinically, HF is broadly classified into ischemic cardiomyopathy (ISCM) and non-ischemic cardiomyopathy (NISCM) [14]. ISCM is primarily caused by obstructive CAD leading to impaired myocardial perfusion and reduced cardiac output, typically due to atherosclerotic plaque buildup [15]. In contrast, NISCM represents a heterogeneous group of conditions with diverse etiologies, including genetic mutations, autoimmune disorders, and metabolic abnormalities [16]. The prevalence, presentation, and outcomes of NISCM vary considerably across populations and are influenced by demographic, geographic, and socioeconomic factors [17]. A recent review by Elendu et al. [18] provides a comprehensive overview of HF, highlighting the interplay of genetic predisposition, hypertension, and coronary artery disease in its development. The review also explores key mechanisms such as cardiac remodeling, neurohormonal activation, endothelial dysfunction, and mitochondrial impairment.

Despite considerable advances in pharmacological therapies, device-based interventions, and evidence-based care strategies, HF remains a complex and heterogeneous syndrome with a high clinical and economic burden [19,20]. Although incidence rates have stabilized in high-income countries, global prevalence continues to rise, driven by aging populations, improved survival following myocardial infarction, and the availability of life-prolonging therapies [20]. In addition, significant regional variation in HF epidemiology persists, with limited data from low- and middle-income countries where HF may present with different features [21].

Diagnosis of HF is primarily based on clinical evaluation, including symptom assessment and physical examination, supported by imaging techniques. Echocardiographic assessment, particularly LVEF quantification, remains the cornerstone of HF phenotypic characterization. Complementary imaging modalities including chest radiography, cardiac magnetic resonance imaging (MRI), computed tomography (CT), and coronary angiography (when coronary artery disease is suspected) provide additional diagnostic information for etiological determination and therapeutic planning [22]. Moreover, levels of plasma NPs, particularly B-type natriuretic peptide (BNP) and N-terminal proBNP (NT-proBNP), are routinely used for the diagnosis and prognosis of HF [23]; however, their diagnostic accuracy is limited in certain clinical contexts—especially in HFpEF—and their circulating levels can be influenced by non-cardiac factors such as renal dysfunction, obesity, and advanced age [24,25,26]. These limitations underscore the need for more precise, phenotype-specific biomarkers to improve diagnostic precision and guide individualized therapy. To address these gaps, several emerging soluble biomarkers have been proposed that reflect additional aspects of HF pathophysiology, including myocardial injury, fibrosis, inflammation, and extracellular matrix remodeling. High-sensitivity cardiac troponins (hs-cTn), galectin-3 (Gal-3), and soluble suppression of tumorigenicity-2 (sST2) have shown promise for enhancing risk stratification and may provide incremental prognostic value when used alongside NPs [27,28]. However, these alternative biomarkers also exhibit several limitations (discussed further below).

Building on the ongoing pursuit of novel soluble biomarkers, recent years have seen growing interest in circulating nucleic acids (cNAs), including cell free DNA (cfDNA) and non-coding RNAs (ncRNAs), as promising diagnostic and prognostic tools in HF. Among ncRNAs, microRNAs (miRNAs) have emerged as especially compelling candidates, given their potential not only as biomarkers for disease detection and risk stratification but also as therapeutic targets, owing to their regulatory influence on key pathophysiological pathways. MiRNAs modulate diverse biological processes relevant to HF progression, including apoptosis, inflammation, fibrosis, angiogenesis, and metabolic remodeling [29,30,31]. Notably, miRNAs are detectable in peripheral circulation and exhibit remarkable stability, largely due to their encapsulation within extracellular vesicles or binding to protective proteins (reviewed in [32]). However, the clinical translation of circulating miRNAs (c-miRNAs) remains limited by several challenges, such as inter-platform variability, lack of standardized pre-analytical and analytical protocols, and insufficient large-scale, multicenter validation studies [33].

Despite considerable advances, no single biomarker class can fully capture the multifactorial and dynamic pathophysiology of HF. Recognizing this, both the new 2023 ESC and the 2022 American Heart Association/American College of Cardiology/Heart Failure Society of America (AHA/ACC/HFSA) guidelines now support a multi-marker approach to improve diagnostic accuracy, risk stratification, and therapeutic decision-making [3,34]. Combining biomarkers such as NPs, soluble sST2, Gal-3, and hs-TnT/I enables clinicians to assess diverse yet complementary biological pathways—including myocardial stretch, fibrosis, inflammation, oxidative stress, and cardiomyocyte injury. Emerging evidence suggests that integrating these biomarkers within a liquid biopsy (LB) framework can yield a more comprehensive molecular snapshot of disease status. Building on this integrative strategy, c-miRNAs offer an additional and highly promising layer of information. Through their regulatory control of fundamental processes such as apoptosis, fibrosis, angiogenesis, and metabolic remodeling, c-miRNAs may enhance the sensitivity and specificity of current biomarker panels in a multi-analyte LB [35].

This narrative review aims to explore the potential of combining c-miRNAs with other soluble biomarkers as a strategy for the prevention, early detection, stratification, and monitoring of HF progression. We begin by discussing the LB concept and summarizing key soluble biomarkers currently recommended or under investigation for HF management. Next, we examine the biological functions and clinical relevance of miRNAs, emphasizing their diagnostic, prognostic, and therapeutic potential. Finally, we review studies that have evaluated the combined use of c-miRNAs with established biomarkers, including Gal-3, sST2, and hs-TnT/I, within a multi-analyte LB framework. Through this synthesis, we aim to elucidate the added value of c-miRNAs in advancing precision medicine for HF.

## 2. Methods

### 2.1. Search Strategy

This narrative review followed the Scale for the Assessment of Narrative Review Articles (SANRA) guidelines [36] to ensure methodological rigor and transparency. A targeted literature search was conducted using PubMed, Scopus, and Web of Science, covering publications up to June 2025. Boolean operators were used to combine search terms relevant to the topic, including: “heart failure”, “chronic heart failure”, “microRNA”, “circulating miRNA”, “cardiac biomarkers”, “prognostic biomarkers”, “diagnostic biomarkers”, “therapeutic targets”, and “miRNA and heart failure subtypes”. Additional search terms such as “natriuretic peptides”, “NT-proBNP”, “galectin-3”, “sST2”, “troponin” and “multi-marker strategy” were included to capture studies exploring the integration of miRNAs with conventional biomarkers.

### 2.2. Eligibility Criteria

We included peer-reviewed articles published in English that examined the role of miRNAs and/or c-microRNAs in the diagnosis, prognosis, pathophysiology, or treatment of HF. Eligible publications encompassed original research articles (clinical, translational, and mechanistic), systematic reviews, meta-analyses, clinical guidelines, and select high-impact narrative reviews. Exclusion criteria were: (i) articles not published in English, (ii) preclinical animal-only studies without translational relevance, and (iii) abstracts, conference proceedings, and unpublished materials such as theses or dissertations.

### 2.3. Study Selection Process

Titles and abstracts were first screened for relevance, followed by full-text review of eligible articles. Two reviewers (E.C. and K.F.) independently assessed each study for inclusion. Any disagreements were first discussed between the reviewers, and if unresolved, a third reviewer (C.P.) was consulted to reach consensus. The reference lists of key articles and recent reviews were manually scanned to identify additional relevant studies.

### 2.4. Quality Appraisal

Although this is a narrative review, a brief critical appraisal of the included literature was undertaken. For narrative and expert reviews, the SANRA checklist was used to evaluate comprehensiveness, synthesis quality, and critical perspective. For empirical studies, we applied the following criteria (i) clarity of study design, (ii) appropriateness of miRNA detection and analysis methods, and (iii) strength and validity of reported conclusions. Studies with significant methodological limitations were excluded unless they offered historical context or valuable conceptual insights.

### 2.5. Data Synthesis

Key information from the selected studies, including study design, patient population, miRNAs investigated, biomarker roles (diagnostic, prognostic, therapeutic), and integration with other biomarkers, was extracted and synthesized narratively. Thematic categories were developed inductively, focusing on (i) molecular mechanisms linking miRNAs to HF pathophysiology, (ii) diagnostic and prognostic potential of specific c-miRNAs, (iii) therapeutic relevance, and (iv) multi-marker strategies incorporating miRNAs and conventional biomarkers. This structured approach allowed the integration of heterogeneous evidence into a cohesive overview aligned with the aims of the review.

### 2.6. Use of Generative AI Tools

ChatGPT-4.0 (OpenAI; https://chat.openai.com, accessed on 14 July 2025) was used to refine the manuscript text originally drafted by the authors. The following prompt was provided: “Improve the text below for clarity, coherence, flow, and grammar for an academic audience.” The authors reviewed and edited the output and take full responsibility for the final content.

## 3. Liquid Biopsy in Heart Failure: Established Biomarkers and the Unmet Need for Innovation

LB, the molecular profiling of biomarkers from non-solid biological specimens, primarily peripheral blood, offers tissue-level diagnostic insights with minimal procedural risk [37]. Initially developed for oncology applications, LB is now being explored for cardiovascular diseases (CVDs), particularly for detecting myocardial injury and monitoring HF progression [38]. When coupled with advanced genomic and proteomic technologies, LB enables dynamic monitoring of disease status and offers a powerful tool for precision medicine [39]. Several soluble biomarkers have been proposed to complement traditional imaging approaches in HF diagnosis and management (discussed further below).

However, the principal challenge in cardiovascular applications lies not in the sampling technique itself, but in identifying circulating biomarkers that combine high sensitivity with cardiac specificity, particularly in inflammatory or infiltrative myocardial diseases [40]. The clinical utility of LB is particularly compelling in its potential to detect early molecular changes associated with HF progression, such as recurrent myocardial injury, mitochondrial dysfunction, apoptosis, and inflammation, even before overt symptoms emerge. Such precision diagnostics may prove especially valuable in differentiating between HF phenotypes (e.g., HFpEF vs. HFrEF), thereby enabling timely and tailored therapeutic interventions. This is crucial, as preventing the transition from HFpEF to HFrEF can significantly alter long-term clinical outcomes [41].

As discussed earlier, both the 2023 ESC and the 2022 AHA/ACC/HFSA guidelines both underscore the central role of NPs in the diagnosis and prognostic assessment of HF [3,34]. Moreover, the 2022 AHA/ACC/HFSA guidelines recommend the use of Gal-3 and sST2 as adjunctive biomarkers for risk stratification and outcome prediction [34]. Notably, Gal-3 and sST2 are useful in identifying patients with increased fibrotic or inflammatory burden, although their specificity is reduced by elevations observed in other inflammatory diseases such as pneumonia and chronic obstructive pulmonary disease [42]. Additionally, the 2023 ESC guidelines recommend the use of hs-cTn in cases of suspected myocardial injury, particularly for distinguishing acute coronary syndromes (ACS) from other causes of myocardial stress in patients presenting with unexplained decompensated HF [3]. The 2022 AHA/ACC/HFSA guidelines emphasize the prognostic value of hs-cTn in CHF They specifically recommend its measurement in individuals with structural heart disease (stage B) or symptomatic HF (stages C), even in the absence of acute coronary symptoms, to support more accurate risk stratification and prognostication [34]. Furthermore, troponins are often chronically elevated in HF due to ongoing myocardial stress rather than acute ischemia, which may complicate interpretation in the setting of comorbidities like renal dysfunction or sepsis [43].

Beyond these, several emerging soluble biomarkers are under active investigation for their potential roles in HF pathophysiology (Table 1). These analytes are thought to reflect a range of pathological mechanisms, including volume overload, neurohormonal activation, oxidative stress, and renal impairment.

However, despite their promising mechanistic relevance, none of these alternative biomarkers have yet received formal endorsement in clinical practice guidelines, largely due to inconsistent performance across patient populations and insufficient validation in large, diverse cohorts. This underscores the ongoing need for more robust, reproducible, and phenotype-specific molecular tools that can reliably augment current HF diagnostic and prognostic strategies.

**Figure 1:**
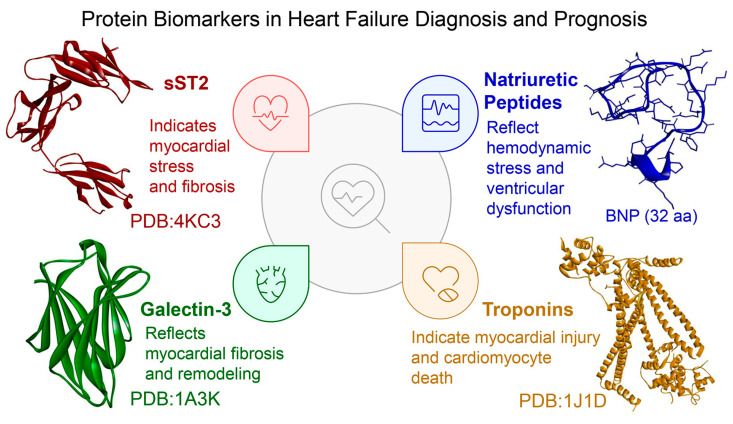
Key protein biomarkers clinically used for heart failure (HF) diagnosis and prognosis. The figure depicts soluble suppression of tumorigenicity 2 (sST2, PDB: 4KC3), galectin-3 (Gal-3, PDB: 1A3K), and cardiac troponin (cTn, PDB: 1J1D), with 3D structures retrieved from the RCSB Protein Data Bank (https://www.rcsb.org/, accessed on 1 July 2025) and visualized via BIOVIA Discovery Studio Visualizer 2025 (Dassault Systèmes, San Diego, CA, USA). The sequence of B-type natriuretic peptide (BNP, 32 amino acids: SPKMVQGSGCFGRKMDRISSSSGLGCKVLRRH) was obtained from [45] and its 3D conformation was predicted using ColabFold (AlphaFold2 implementation, https://colab.research.google.com/github/sokrypton/ColabFold/blob/main/AlphaFold2.ipynb, accessed 1 July 2025) and visualized via BIOVIA Discovery Studio Visualizer 2025. Each biomarker is annotated with its principal clinical role: sST2 reflects myocardial stress and fibrosis; Gal-3 signals myocardial fibrosis and remodeling; natriuretic peptides (e.g., BNP) indicate hemodynamic stress and ventricular dysfunction; and troponins mark myocardial injury and cardiomyocyte death.

In conclusion, current HF management, alongside imaging techniques, incorporates four key classes of soluble biomarkers strongly recommended by the 2023 ESC and 2022 AHA/ACC/HFSA guidelines: NPs, Gal-3, sST2, hs-TnT/I, each reflecting distinct pathophysiological processes (Figure 1); below, we provide a concise overview of these biomarkers (reviewed in [44]). Although other candidate biomarkers (listed in Table 1) may hold clinical promise, they are not discussed in detail within the scope of this review.

### 3.1. Established yet Imperfect: The Role of Natriuretic Peptides in Heart Failure Stratification

NPs are a family of structurally related cardiac biomarkers secreted in response to myocardial stretch and biomechanical stress. The principal members include atrial natriuretic peptide (ANP), BNP, and C-type natriuretic peptide (CNP), with MR-proANP also receiving increasing attention. These peptides exert cardioprotective effects by antagonizing the renin–angiotensin–aldosterone system (RAAS), promoting vasodilation, natriuresis, and diuresis, and limiting cardiac fibrosis and hypertrophy [46].

Among the NPs, BNP and its cleavage product, NT-proBNP, are the most extensively validated and clinically used biomarkers for HF [3,34]. Released in proportion to ventricular wall stress, both BNP and NT-proBNP provide robust diagnostic and prognostic information across a range of HF phenotypes [47,48,49]. NT-proBNP, in particular, has demonstrated high negative predictive value (NPV ≈ 0.88–0.98) for ruling out HF, although reference thresholds vary by age, sex, and comorbidity profiles [50]. These markers also correlate with disease severity and predict future cardiovascular events and long-term outcomes—even in asymptomatic patients with structural cardiac abnormalities [51,52].

Despite their widespread utility, important limitations must be acknowledged. NPs levels can be elevated in non-cardiac conditions such as chronic kidney disease, pulmonary embolism, and respiratory failure, thereby reducing diagnostic specificity [53]. Conversely, factors such as obesity and peripheral edema may suppress NP concentrations, potentially leading to underdiagnosis [54,55]. Sex, age, and race also influence their baseline levels [56,57,58]. Additionally, diagnostic sensitivity may decline at higher cut-off values or in HF with HFpEF, where wall stress may be insufficient to provoke strong NP elevations [59].

### 3.2. Galectin-3: A Fibrosis-Related Biomarker with Prognostic Potential in Heart Failure

In recent years, there has been growing interest in multi-marker strategies that integrate indicators of inflammation, fibrosis, and cellular injury to enhance phenotyping and improve patient stratification in HF. In line with this approach, Gal-3 has emerged as a supplementary biomarker to aid in risk stratification for both AHF and CHF [34]. Notably, since 2014, Gal-3 has been approved by the U.S. Food and Drug Administration (FDA) for use in assessing risk in HF patients [60]. Its value lies in providing prognostic information that is both independent of and complementary to established biomarkers such as natriuretic peptides and high-sensitivity cardiac troponins [61,62].

Biologically, Gal-3 is a β-galactoside-binding lectin expressed in immune cells, epithelial cells, and sensory neurons [63]. It plays a critical role in fibrotic remodeling by regulating apoptosis, inflammation, angiogenesis, and extracellular matrix turnover. In the healthy mouse heart, Gal-3 is constitutively expressed in macrophages and localizes primarily to atrial but not ventricular cardiomyocytes [64], suggesting its potential involvement in the early stages of cardiac remodeling.

Epidemiological data from large cohort studies support the clinical utility of Gal-3. In the Framingham Offspring Study (n = 3353), elevated Gal-3 levels were independently associated with a higher risk of incident HF and all-cause mortality [65]. Similarly, findings from a prospective study of 5958 participants demonstrated that elevated Gal-3 concentrations were predictive of HF onset and adverse cardiovascular outcomes [66]. Gal-3 has also been associated with pathological ventricular remodeling, a key driver of HF progression [67].

Although Gal-3 offers potential as a biomarker, its use is limited by the fact that it is not specific to the heart. Its levels are elevated in various non-cardiac conditions, including chronic kidney disease, liver disease, malignancy, and systemic inflammation. Moreover, Gal-3 concentrations are influenced by age, sex, renal function, and comorbid inflammatory states. A further challenge is the lack of assay standardization across commercial platforms, which hampers comparability between studies and limits routine clinical implementation [68]. The diagnostic and prognostic roles of Gal-3 in HF, along with its limitations, have been extensively reviewed in the literature [68,69,70,71].

### 3.3. Soluble ST2: A Prognostic Biomarker Reflecting Myocardial Stress and Remodeling

Suppression of tumorigenicity-2, a member of the interleukin-1 receptor family, has emerged as a promising prognostic biomarker in heart HF. It exists in two primary isoforms: a membrane-bound form (ST2L) and a soluble circulating form (sST2) [72]. The functional ligand for ST2L is interleukin-33 (IL-33), a cytokine released in response to cellular stress, necrosis, or inflammation. IL-33 functions as an “alarmin,” triggering protective immune responses and exerting cardioprotective effects by attenuating fibrosis, limiting hypertrophy, and promoting cardiomyocyte survival [73,74]. However, sST2 acts as a decoy receptor, binding IL-33 in the circulation and inhibiting its beneficial effects, thereby facilitating myocardial fibrosis and adverse cardiac remodeling [75].

Clinically, elevated sST2 levels are strongly associated with worse outcomes in both AHF and CHF. They serve as independent predictors of all-cause mortality and HF-related hospitalization [75,76]. Notably, a key advantage of sST2 over traditional NP biomarkers is its biological stability across a wide range of clinical contexts. Unlike BNP or NT-proBNP, sST2 levels are minimally affected by age, sex, renal function, and body mass index [72,77]. This makes it particularly useful in cohorts where NPs may be less reliable, including those with obesity, renal dysfunction, or advanced age.

Nonetheless, several limitations restrict the widespread clinical adoption of sST2. Its lack of cardiac specificity remains a significant drawback, as elevated levels can occur in various non-cardiac inflammatory conditions, including sepsis, pneumonia, chronic obstructive pulmonary disease, and systemic infections [78]. Furthermore, standardized reference ranges and universally accepted cut-off values are lacking, leading to variability across commercial assays and complicating inter-study comparisons [79].

In summary, sST2 provides valuable prognostic information in HF and holds promises for inclusion in multi-marker strategies. However, additional standardization, validation in randomized clinical trials, and clarification of its role in therapeutic decision-making are necessary before broader clinical implementation can be recommended.

### 3.4. High-Sensitivity Cardiac Troponins: Markers of Myocardial Injury and Prognosis in Heart Failure

Cardiac troponins—troponin T (cTnT) and troponin I (cTnI)—are integral components of the cardiomyocyte contractile machinery and are highly specific biomarkers of myocardial injury [80]. These proteins are part of a three-subunit complex that also includes troponin C (TnC); however, only cTnT and cTnI are specific to cardiac tissue. Of the two, cTnI is particularly valued for its superior sensitivity and specificity, making it a key biomarker for diagnosing ACS and stratifying cardiovascular risk. Its clinical utility is further underscored by its designation as the preferred marker in the Third Universal Definition of Myocardial Infarction [80].

Troponins are released into the bloodstream primarily following necrotic cell death; however, they may also leak through compromised sarcolemmal membranes in the absence of overt necrosis [81]. In the context of HF, elevated troponin levels reflect a spectrum of pathological processes, including myocardial ischemia (due to epicardial or microvascular dysfunction), neurohormonal activation, inflammation, infiltrative cardiomyopathies, apoptosis, autophagy, and increased cardiomyocyte turnover [81].

In stable CHF, conventional assays detect elevated troponin levels in approximately 10–60% of patients. High-sensitivity cTn (hs-cTn) assays, however, detect measurable concentrations in nearly all cases, with a significant proportion exceeding the 99th percentile threshold established for healthy individuals [81]. Notably, hs-cTnI has demonstrated strong prognostic utility across various HF populations, allowing for earlier and more accurate identification of myocardial injury [82]. In patients with HF with reduced HFrEF, elevated troponin levels are consistently associated with increased risks of hospitalization and mortality [83,84]. In addition, elevated troponin levels in HF are associated with a hazard ratio of 2.85 (95% CI: 2.02–4.03) for mortality, underscoring their strong predictive value [85].

However, the enhanced sensitivity of hs-cTn assays comes at the cost of reduced specificity. Elevated troponin concentrations can also occur in a range of non-ischemic and non-cardiac conditions, including CHF, renal impairment, sepsis, systemic inflammation, and intense physical exertion [86,87]. In patients with chronic kidney disease (CKD), persistent troponin elevation may result from both impaired renal clearance and subclinical myocardial stress [88]. Importantly, while elevated troponin confirms the presence of myocardial injury, it does not reveal its underlying cause [89]. The temporal profile of troponin release further complicates interpretation in HF [90].

Furthermore, despite their prognostic significance, several limitations constrain the broader application of troponins in HF management. First, their limited specificity to cardiac pathology reduces diagnostic accuracy. Second, the near-universal detection of troponins in CHF, especially with hs assays, complicates differentiation between physiological and pathological elevations. Third, variability among commercial assay platforms hinders standardization and limits longitudinal comparability. Fourth, unlike in ACS, troponin levels in HF show a continuous relationship with risk. Therefore, using simple “positive/negative” cut-offs is clinically inadequate in the HF setting. Finally, although elevated troponin levels identify high-risk individuals, there is insufficient evidence that serial troponin monitoring improves clinical outcomes. As such, their role in HF remains predominantly prognostic rather than therapeutic [90].

### 3.5. Circulating Free Nucleic Acids: A New Era of Biomarkers for Heart Failure Management?

CNAs, including DNA and various RNA species, represent a novel class of biomarkers isolated from cell-free plasma, serum, or other body fluids such as lymph, cerebrospinal fluid, urine, and bronchial lavage [91]. When derived specifically from cell-free sources, these are collectively termed cell-free nucleic acids (cfNAs), which include both cell-free DNA (cfDNA) and cell-free RNAs (cfRNAs) [91,92]. These biomarkers are increasingly being recognized for their potential in the early detection, monitoring, and prognostication of CVDs, including HF [93]. Epigenetic mechanisms such as DNA methylation, histone modifications, and ncRNAs (especially miRNAs) play central roles in regulating cardiomyocyte apoptosis and necrosis without altering DNA sequences and are critically involved in HF pathogenesis [94]. Advances in next-generation sequencing (NGS) have further expanded the potential of LB assays integrating both cfDNA and miRNAs to support precision medicine approaches in HF management [95]. The molecular perturbations driving pathological cardiac remodeling, characterized by progressive ventricular dilation and impaired contractility, precede the clinical onset of HF symptoms. This temporal disconnect underscores the potential value of sensitive, network-informed biomarker panels for early detection and intervention.

#### 3.5.1. The Potential of Cell-Free DNAs as a Biomarkers in Heart Failure

CfDNA comprises short, double-stranded fragments typically ranging from 160–180 bp, with a predominant size around 167 bp [96]. These fragments are released into circulation from diverse sources including the nucleus, mitochondria, damaged or apoptotic cells, and even transplanted or infected tissues. Despite their short half-life, cfDNA fragments are continually replenished, offering dynamic snapshots of genetic and epigenetic states [97].

Under physiological conditions, cfDNA concentrations in healthy individuals range between 7–18 ng/mL and are cleared primarily by the liver [98]. Elevated cfDNA levels reflect acute cellular injury or apoptosis and significantly contribute to inflammatory responses [99]. In HF patients, elevated cfDNA levels correlate with disease severity and cellular damage intensity [100]. Notably, unmethylated cfDNA fragments from the refilin A (FAM101A) gene specifically trace cardiomyocyte apoptosis during ischemic events, complementing existing biomarkers [101]. Additionally, elevated mitochondrial cfDNA has been linked to increased mortality in acute severe HF, suggesting utility in risk stratification [102]. Therapeutic interventions can modulate cfDNA levels, as demonstrated by studies showing that levosimendan infusion in ischemic HF patients with severe left ventricular dysfunction significantly decreased plasma cfDNA levels, correlating with improved echocardiographic and biochemical indices of cardiac function [103].

The potential of cfDNAs as biomarkers has been evaluated in recent studies. Berezina et al. [104] prospectively enrolled 452 chronic HFrEF patients initiating guideline-directed therapy to determine whether baseline and six-month changes in circulating cell-free nuclear DNA (cf-nDNA) predict systolic recovery. They defined HFimpEF as ≥10% LVEF gain to >40% at six months (n = 177 vs. persistent HFrEF, n = 275). A cf-nDNA threshold of ≤7.5 µmol/L yielded an area under the curve (AUC) of 0.875 (87% sensitivity, 73.5% specificity) for HFimpEF and remained an independent predictor in multivariable models (OR 1.64; 95% CI 1.10–2.07; *p* = 0.001), even after adjustment for NT-proBNP dynamics, ischemic etiology, NYHA class, and other clinical covariates.

Salzano et al. [105] evaluated the prognostic relevance of cfDNA in 71 stable HF patients with reduced ejection fraction (EF < 50%) compared with 64 healthy controls over a 30-month follow-up period. cfDNA levels were significantly elevated in the HF group, and patients with cfDNA concentrations above the median demonstrated significantly lower event-free survival. Importantly, receiver operating characteristic (ROC) curve and net reclassification index (NRI) analyses revealed that adding cfDNA to BNP levels improved risk stratification better than the use of BNP alone.

Zhang et al. [106] conducted a pilot prospective cohort study of 98 acute myocardial infarction (AMI) patients to determine whether plasma cfDNA levels at hospital admission predict subsequent HF development. During a median follow-up of 345 days, 46 patients (52.6%) developed HF. Individuals with cfDNA above the cohort median (14.39 ng/mL) had higher LDL-C, cTnI, and sST2 levels. In multivariate Cox models, cfDNA >9.227 ng/mL independently predicted HF incidence (adjusted HR 2.81; 95% CI 1.09–7.24; C-index 0.74; *p* = 0.033), with a dose–response relationship after adjusting for age, sex, and chronic kidney disease history.

Collectively, these findings underscore the growing clinical relevance of cfDNA as a non-invasive, dynamic biomarker for HF diagnosis, prognosis, and treatment monitoring. As evidence continues to accumulate, cfDNA profiling holds promise for integration into precision cardiology workflows, offering new avenues for earlier intervention and more personalized disease management.

#### 3.5.2. Cell-Free RNAs: Emerging Biomarkers for Heart Failure Management?

In parallel with advances in cfDNA analysis, interest has surged in cfRNAs—particularly ncRNAs such as miRNAs, lncRNAs, and circRNAs—as emerging biomarkers in cardiovascular medicine [107]. Among the various ncRNAs, miRNAs have attracted significant attention as prognostic, diagnostic, and therapeutic biomarkers for cardiovascular diseases, including HF. Their appeal stems from their unique properties: miRNAs are highly tissue-specific, play key regulatory roles, and exhibit remarkable stability in the bloodstream (discussed further below). Compared with cfDNA, these qualities make miRNAs particularly promising for clinical applications. This is supported by the recent systematic review by Lewandowski et al. [108] who evaluated the utility of circulating cfDNA and miRNAs as biomarkers of myocardial inflammation, with a focus on myocarditis and inflammatory dilated cardiomyopathy (InfDCM). Among 1185 screened records, 56 studies met inclusion criteria and reported 187 unique miRNAs. The review identified miR-Chr8:96, miR-155, and miR-206 as the most promising diagnostic candidates, with some achieving AUC values exceeding 0.90.

Overall, while cfDNA provides valuable information on genetic and epigenetic changes, miRNAs offer insight into the real-time regulation of gene expression. This distinction is crucial: although higher cfDNA levels are linked to acute cellular injury and inflammation in HF, the broader application of cfDNA in CHF care remains under investigation and requires further validation.

Given their growing significance as both biomarkers and therapeutic agents, this review highlights the central role of miRNAs in HF pathophysiology and management. The following sections will explore their biological functions, diagnostic and prognostic value, and how they may be integrated into multi-marker approaches—demonstrating their significant potential to advance precision management of HF.

## 4. The Multiple Roles of miRNAs in Heart Failure

### 4.1. An Overview of miRNA Biogenesis, Function and Circulation

MiRNA biogenesis and function have been extensively reviewed elsewhere [32,109]; here, we provide below a concise summary to contextualize their relevance in HF. MiRNAs are ~22-nucleotide non-coding RNAs that regulate gene expression post-transcriptionally by promoting mRNA degradation or translational repression [110]. Dysregulated miRNA expression is linked to diverse diseases such as cancer, cardiovascular, and immune disorders [111]. Conserved across species, most human miRNAs arise from non-coding regions or introns, though some derive from protein-coding exons [112]. In the canonical pathway, primary miRNAs (pri-miRNAs) are transcribed and processed in the nucleus by the Drosha-DGCR8 complex into precursor miRNAs (pre-miRNAs) with characteristic 3′ overhangs [113]. Pre-miRNAs are exported to the cytoplasm via exportin-5/RanGTP and cleaved by Dicer into ~22-nt duplexes [113]. One strand as the guide is loaded into Argonaute (AGO) to form the RNA-induced silencing complex (RISC), which targets complementary mRNAs for repression, while the passenger strand is typically degraded [114]. Non-canonical pathways bypass Drosha and/or Dicer. These include mirtrons (spliced introns mimicking Dicer substrates), Dicer-independent pathways involving AGO2-mediated cleavage, and m7G-capped pre-miRNAs exported via exportin-1 [115,116]. Additionally, Drosha and Dicer may directly regulate protein-coding transcripts, indicating broader roles beyond miRNA maturation [117].

The presence of miRNAs in the circulation was first reported in human blood in 2008, when they were detected in plasma, erythrocytes, platelets, and other nucleated blood cells [118]. Endogenous plasma miRNAs remain strikingly stable even after extreme pH, boiling, repeated freeze-thaw cycles, or prolonged room-temperature storage, whereas synthetic miRNA spikes are quickly degraded by plasma RNases [118]. MiRNAs enter the bloodstream both passively, through cell damage, and actively, via secretion. Once extracellular, they partition into two main pools, as follows:

- Vesicle-encapsulated miRNAs packaged in exosomes, microvesicles, or apoptotic bodies [119].
- Vesicle-free miRNAs complexed with RNA-binding proteins such as Argonaute-2 (Ago2) or nucleophosmin-1 (NPM1), or carried by high-density lipoprotein (HDL) [120,121].

The dominance of each pool depends on the miRNA species, its cellular source, and the physiological or pathological context. In most biofluids, protein-bound miRNAs predominate [121,122], although vesicle-encapsulated forms can be more abundant in serum or saliva [121]. Binding to either proteins or vesicles shields miRNAs from RNase activity, accounting for the extraordinary stability of endogenous circulating miRNAs—and the rapid degradation observed for synthetic counterparts [123].

Beyond their diagnostic value, c-miRNAs are increasingly recognized for their role in cell-to-cell communication. Encapsulated miRNAs can be selectively secreted by donor cells and internalized by recipient cells, modulating gene expression in an autocrine, paracrine, or endocrine fashion [124,125]. This intercellular transfer of miRNAs suggests a dual role in both signaling and systemic regulation of disease processes.

C-miRNAs are now front-line candidates for non-invasive cardiovascular diagnostics because they combine exceptional stability in biofluids with tissue-restricted expression and rapid responsiveness to cellular stress (reviewed in [126]). Found in plasma, serum, and extracellular vesicles, these molecules mirror molecular events in the heart and vasculature, allowing insight into disease onset, progression, and therapy response.

Intense effort is focused on HF [29,127], but c-miRNAs are also being evaluated for myocardial infarction [128], atherosclerosis [129], type 2 diabetes mellitus [130] and hypertension [131]. Their diagnostic power hinges on how they enter and persist in the circulation, whether they are released passively from injured cells or secreted in vesicles and protein complexes that protect them from RNases. Clarifying these release and stabilization mechanisms is essential for translating c-miRNA signatures into clinical tests for HF and other cardiovascular conditions. Similar principles underpin their expanding use as biomarkers in oncology and neurodegenerative disorders [132,133].

### 4.2. MiRNA Regulation of Key Pathophysiological Mechanisms in Heart Failure

As stated above, HF is characterized by the heart’s inability to pump sufficient blood to meet the body’s metabolic demands, often resulting from structural or functional cardiac impairments [3,21]. The clinical syndrome of HF encompasses a spectrum of etiologies, including ischemic heart disease, hypertension, valvular disorders, and cardiomyopathies, each contributing to distinct yet overlapping pathophysiological cascades [134]. At the molecular level, HF progression involves a complex interplay of maladaptive processes such as myocardial hypertrophy, interstitial fibrosis, chronic inflammation, endothelial dysfunction, apoptosis, and impaired angiogenesis [135,136].

Traditional paradigms focused on neurohormonal activation, particularly the renin–angiotensin–aldosterone system and sympathetic nervous system. However, recent advances have illuminated the pivotal role of ncRNAs, particularly miRNAs, as master regulators of these multifaceted biological pathways [137]. MiRNAs exert their effects by fine-tuning gene expression networks that govern cardiac remodeling, immune responses, vascular homeostasis, and cardiomyocyte survival [138]. Dysregulated miRNA expression contributes to the onset and progression of HF, influencing the balance between compensatory and pathological remodeling processes [139]. Their stability in circulation, tissue specificity, and responsiveness to physiological stress underscore their potential as biomarkers and therapeutic targets.

In the following sections, we discuss the roles of miRNAs in myocardial hypertrophy, fibrosis, inflammation, apoptosis, and angiogenesis, which are central mechanisms driving HF progression. By elucidating these pathways, we aim to clarify how distinct miRNA signatures contribute to HF onset and progression, while also evaluating their potential as diagnostic biomarkers and therapeutic targets. Representative examples are summarized in Table 2.

It should be noted that while miRNAs have also been implicated in other critical aspects of HF pathogenesis—such as electrical remodeling (miR-1, miR-30d, miR-21, miR-328) [140,141,142], metabolic dysfunction (miR-378, miR-499, miR-696, miR-532, miR-690, miR-345-3p) [143,144], and skeletal muscle wasting (miR-21, miR-133a, miR-434-3p, miR-424-5p, miR-455-mir-181a) [145], these areas fall beyond the scope of this review.

**Table 2:** Representative miRNAs involved in key pathophysiological processes of heart failure.

Pathophysiological Process	Representative miRNAs	Functional Role	Ref.
Cardiac hypertrophy	miR-21, miR-208a, miR-1, miR-340	Modulate hypertrophic signaling and fetal gene expression	[140,146,147,148,149]
Fibrosis	miR-29, miR-133a, miR-21	Regulate fibroblast activation, collagen synthesis, ECM turnover	[150,151]
Inflammation	miR-146a, miR-125, miR-21, miR-155	Modulate cytokine expression, macrophage polarization, and inflammatory signaling	[152,153,154]
Apoptosis	miR-15 family, miR-34a, miR-195	Regulate apoptosis-related genes (Bcl-2, SIRT1, Notch1) and cardiomyocyte survival	[155,156,157]
Angiogenesis	miR-126, miR-210, miR-92a	Promote or inhibit neovascularization; regulate VEGF signaling and endothelial function	[158,159]

#### 4.2.1. MiRNA Regulation of Cardiac Hypertrophy and Structural Remodeling

In HF, the myocardium undergoes structural remodeling marked by cardiomyocyte hypertrophy and excessive extracellular matrix (ECM) deposition, often resulting in fibrosis [135]. Connective tissue growth factor (CTGF) is a major fibrotic mediator and therapeutic target [160]. While CTGF’s transcriptional regulation is well studied, its post-transcriptional control by miRNAs remains less understood. Cardiac hypertrophy begins as an adaptive response to biomechanical or neurohormonal stress but becomes maladaptive with chronicity, contributing to HF progression [136]. This involves cardiomyocyte enlargement, increased protein synthesis, and reactivation of fetal gene programs [161].

MiRNAs have emerged as key post-transcriptional regulators, capable of promoting or suppressing hypertrophic signaling [162]. By targeting multiple pathways, they govern the shift from compensatory to maladaptive remodeling and are increasingly viewed as both biomarkers and therapeutic targets in HF [163,164]. Certain miRNAs mediate protective or pathological cardiac responses (Table 3).

While cardiomyocyte hypertrophy represents an initial adaptive response to cardiac stress, persistent hypertrophic signaling ultimately triggers secondary pathological processes, including excessive extracellular matrix deposition and myocardial fibrosis, which further compromise cardiac function and represent distinct therapeutic targets.

#### 4.2.2. MiRNA Regulation of Myocardial Fibrosis and Extracellular Matrix Dysregulation

Myocardial fibrosis disrupts cardiac architecture, promotes arrhythmias, and worsens HF outcomes [174]. Cardiac fibroblasts orchestrate ECM production and turnover, but under pathological stress, they proliferate through local expansion, progenitor recruitment, and endothelial-to-mesenchymal transition [175]. While ECM provides structural support, sustained fibroblast activation leads to maladaptive fibrosis.

Fibroblasts maintain ECM balance via matrix metalloproteinases (MMPs) and their inhibitors (TIMPs). Cardiac fibroblasts can be directly reprogrammed into cardiomyocyte-like cells using either key cardiac transcription factors, such as GATA4, MEF2C, HAND2, and TBX5 [176], or a defined microRNA cocktail (‘miR-combo’: miR-1, miR-133, miR-208, miR-499) [177]. Both strategies have yielded induced cardiomyocytes displaying cardiac gene expression, sarcomeric structures, contractility, and calcium flux in vitro and within infarcted hearts in vivo, underscoring their promising therapeutic potential. Several miRNAs critically modulate myocardial fibrosis by regulating ECM synthesis, fibroblast survival, and apoptosis (Table 4).

Pro-fibrotic miRNAs include miR-21, which is upregulated in remodeling fibroblasts and promotes fibrosis by repressing PTEN, Spry1, and TGFBR3, thereby activating ERK–MAPK signaling [178]. MiR-214 and miR-144-3p further drive fibrosis via ERK1/2 activation and PTEN inhibition, respectively [181,182]. Conversely, the miR-29 family (miR-29a/b/c) directly suppresses ECM gene expression, including collagens and laminins. Loss of miR-29 post-infarction exacerbates fibrosis, while its restoration attenuates ECM deposition [180]. MiR-30, especially miR-30c, counters fibrosis by targeting CTGF, although oxidative damage can impair its function [183]. MiR-133 exerts anti-fibrotic effects through modulation of pathways like RhoA/ROCK, TGF-β/Smad, and PI3K/Akt [173]. Notably, miR-133 demonstrates dual protective roles, as it also suppresses cardiac hypertrophy (Table 3), highlighting how certain miRNAs can coordinate multiple aspects of cardiac remodeling. Emerging data also highlight miR-590-3p as an anti-fibrotic and regenerative agent, reducing fibrosis and enhancing cardiac regeneration via TSC22D2 and PKM2 pathway activation [184].

Collectively, miRNAs represent central regulators of myocardial fibrosis and potential therapeutic targets to counteract fibrosis-driven cardiac dysfunction.

#### 4.2.3. The Inflammatory Heart: MiRNA Regulation of Immune Cell Activation in HF

HF is no longer viewed purely as a hemodynamic disorder; it is now recognized as a state of chronic, low-grade inflammation in which persistent activation of innate and adaptive immune pathways fuels maladaptive remodeling and disease progression [185]. Although an early, self-limited inflammatory burst is essential for wound healing, the protracted immune stimulation seen in HF perpetuates cardiomyocyte loss, fibroblast activation and ventricular dysfunction [186]. MiRNAs shape this immune landscape by fine-tuning cytokine networks and immune-cell phenotypes; their actions are summarized in Table 5. Notably, failing hearts harbor miRNA signatures consistent with sustained immune activation [186]. Furthermore, miRNAs, especially those carried in exosomes, orchestrate this inflammatory microenvironment by modulating immune signaling and cell behavior (Table 5).

Among the most influential species is miR-155, which is upregulated in activated macrophages and T-cells; it represses SOCS-1, amplifies NF-κB signaling and intensifies leukocyte infiltration, thereby exacerbating injury in ischaemia reperfusion, viral myocarditis and pressure-overload models [154]. In addition, miR-155 modulates endothelial activation by downregulating NF-κB p65, yet it also suppresses eNOS, contributing to nitric oxide depletion and vascular dysfunction [201]. By contrast, miR-146a/b, induced by NF-κB, targets TRAF6 (and TLR3) to provide a negative-feedback brake on inflammatory cascades [202]. Notably, pmiR-146a/b generally confers vascular protection by inhibiting NF-κB and MAPK pathways; however, their function can be inhibited by the long non-coding RNA MALAT1. Silencing MALAT1 restores miR-146a/b activity, enhancing endothelial stability [203,204].

C-miR-21 shows an inverse relationship with serum TNF-α and IL-6 in HF cohorts, suggesting a modulatory rather than strictly pro-inflammatory role [185]. Additional modulators include miR-223 and the miR-17~92 cluster, which collectively balance immune-cell proliferation and cytokine output [186]. MiR-939-5p, regulated by lncRNA NOS2P3, modulates inflammatory apoptosis in CHF and may serve as a biomarker of immune-driven decompensation [195]. Cardiomyocytes subjected to hypoxia or mechanical strain release extracellular vesicles enriched in miR-146a-5p, miR-10a/b, miR-143, and miR-423; when macrophages take up these vesicles, they polarize toward a pro-inflammatory (M1) phenotype and secrete higher levels of interleukin-6 via activation of the ERK, JNK, and p38 MAPK pathways [194,205]. Exosomal miR-155 is a particularly potent amplifier of this feed-forward circuit [194].

Cardiosphere-derived cells (CDCs) add a counter-vailing influence: their vesicles carry miR-181b, miR-26a, miR-27a-5p and miR-101a, all of which foster M2-like macrophage polarisation, enhance efferocytosis and improve post-infarct healing [196,197]. Endothelial-cell vesicles containing miR-222 or miR-126 repress ICAM-1 and VCAM-1, thereby limiting leukocyte adhesion and transendothelial migration [199].

In summary, both intracellular and exosomal miRNAs—particularly miR-21, miR-146a/b, and miR-155—have dual roles in immune regulation and fibrosis, underscoring their pleiotropic impact on HF pathophysiology (Table 5).

#### 4.2.4. Apoptosis and Cell Survival Signaling: MiRNA-Mediated Control of Cardiomyocyte Fate in Heart Failure

Apoptosis—a tightly orchestrated, energy-dependent form of cell death—is a major driver of cardiomyocyte loss HF [206]. Hemodynamic overload, ischemia-reperfusion, oxidative stress and neuro-hormonal cues converge on caspase cascades, Bcl-2-family shifts and PI3K-Akt imbalance, ultimately curtailing contractile mass and fueling ventricular remodeling [207]. A growing body of work assigns a central regulatory tier to miRNAs, which fine-tune mitochondrial dynamics, autophagy, and death-receptor signaling [208].

Pro-apoptotic miRNAs include miR-103-3p, which targets hepatic leukaemia factor (Hlf) to heighten apoptosis and suppress autophagy [209]; the miR-34 family, whose member miR-34a represses Bcl-2, SIRT1, Notch1 and PNUTS, thereby driving age-, infarction- and diabetes-related myocyte attrition [210]; and the miR-15 family (miR-15a/b, miR-16, miR-195, miR-497), upregulated after ischaemia and shown to silence Bcl-2, Cyclin D2, SIRT3 and c-Myb, with pharmacological antimiR therapy limiting infarct size [211]. miR-30a-5p likewise promotes hypoxia/reoxygenation (H/R) apoptosis by down-regulating the transcription factor E2F3 [212].

By contrast, miR-26a promotes cell survival through PTEN inhibition and downstream PI3K-Akt and JAK/STAT engagement [213], whereas miR-24 directly represses Bim (and p53) to blunt ischemia-reperfusion (I/R)-triggered apoptosis [214]. MiR-214 safeguards mitochondrial integrity and Akt phosphorylation by targeting PTEN and Bim1, thereby reducing cell loss after I/R [215]. Examples of apoptosis-regulating miRNAs, their principal targets and functional direction is provided in Table 6. Several miRNAs (e.g., miR-34a, miR-195, miR-214, miR-30a-5p) recur across hypertrophy, fibrosis, and immune sections, underscoring their integrative therapeutic potential in HF.

#### 4.2.5. Vascular Balance in Heart Failure: MiRNA Regulation of Angiogenesis and Endothelial Dysfunction

Vascular homeostasis is critical for maintaining myocardial perfusion and supporting tissue repair. In HF, impaired angiogenesis and endothelial dysfunction accelerate disease progression by exacerbating hypoxia, promoting fibrosis, and driving maladaptive cardiac remodeling. Endothelial cells, which regulate vascular tone, leukocyte adhesion, and barrier integrity, lose their protective phenotype under oxidative stress, inflammation, and neurohormonal dysregulation, thereby compounding HF pathology [218].

MiRNAs have emerged as key regulators of vascular biology, modulating angiogenesis, endothelial function, and inflammatory signaling [219]. In HF, miRNA dysregulation contributes to reduced perfusion, increased apoptosis, and impaired vascular repair mechanisms [220]. Among the pro-angiogenic miRNAs, miR-126, miR-132, and miR-210 are particularly important. MiR-126 enhances VEGF signaling by targeting SPRED1 and PIK3R2, promoting endothelial proliferation and neovascularization. Its deficiency leads to vascular leakage and impaired neovascularization, while circulating levels correlate with HF severity [130,159]. MiR-132 promotes angiogenesis and reduces fibrosis via the PTEN/PI3K/Akt pathway, with circulating levels predictive of HF severity and rehospitalization risk [221]. Hypoxia-inducible miR-210 supports neovascularization by targeting ephrin-A3 and PTP1B, enhancing VEGF and FGF secretion to facilitate endothelial proliferation and tube formation [222].

Conversely, several miRNAs act as anti-angiogenic or dysfunction-promoting factors. MiR-92a, part of the miR-17–92 cluster, inhibits angiogenesis by targeting ITGA5 and SIRT1; its inhibition restores endothelial gene expression, limits endothelial-to-mesenchymal transition (EndMT), and improves diastolic function in HFpEF models [223,224]. miR-34a, elevated in aging and HF, induces endothelial senescence and dysfunction by suppressing SIRT1 and eNOS; its inhibition improves vascular function and reduces post-MI fibrosis [210]. miR-1285-3p, upregulated in CHF, suppresses endothelial proliferation and induces apoptosis [225]. miR-200b impairs angiogenesis by targeting VEGF-A, VEGFR1/2, ETS1, and GATA2, leading to reduced nitric oxide bioavailability, elevated oxidative stress, and compromised vascular function [226].

A summary of key pro- and anti-angiogenic miRNAs and their regulatory targets is provided in Table 7. Notably, several miRNAs—miR-126, miR-132, miR-210, miR-34a, appear recurrently, underscoring their central roles in endothelial regulation, inflammation, apoptosis, and remodeling. This complex crosstalk highlights the integrative regulatory networks orchestrated by miRNAs in HF pathology.

Furthermore, circulating levels of miR-126 [130], miR-132 [221], and miR-1285-3p [210] have potential as biomarkers of endothelial damage and HF progression. Therapeutic strategies aimed at modulating these miRNAs through mimics or inhibitors represent promising avenues to improve myocardial perfusion and curb HF progression. Nonetheless, significant challenges remain regarding delivery specificity, molecular stability, and off-target effects. These insights underscore the complex and integrative regulatory networks formed by miRNAs in vascular homeostasis, angiogenesis, and endothelial function within the broader landscape of HF pathophysiology.

## 5. Circulating microRNAs in Heart Failure: Diagnostic, Prognostic, and Therapeutic Applications

Beyond their multifaceted roles in HF pathophysiology, miRNAs present in biofluids, particularly miRNAs in the bloodstream, have emerged as promising non-invasive biomarkers for HF diagnosis and prognosis. Their remarkable stability in peripheral blood, coupled with the feasibility of detection via standardized molecular assays, underpins this potential. Importantly, c-miRNAs display reproducible expression patterns that correlate with HF severity, cardiac function, and clinical outcomes. As such, c-miRNAs hold significant potential to complement traditional protein-based biomarkers, such as NT-proBNP and BNP, by providing additional molecular insights into the underlying pathophysiology of HF. Numerous studies have demonstrated the ability of specific c-miRNA signatures not only to discriminate HF patients from healthy controls but also to stratify individuals according to disease subtype, functional class, and prognosis. Examples of c-miRNAs with diagnostic, prognostic, and stratification potential are discussed in more detail below.

### 5.1. Diagnostic Applications of Circulating miRNAs

The identification of specific miRNA profiles has revealed several key diagnostic markers for HF (Table 8).

Tijsen et al. [228] identified six c-miRNAs, namely miR-423-5p, miR-18b, miR-129-5p, miR-1254, miR-675, and miR-622, that were significantly upregulated in patients with HF. Among these, miR-423-5p emerged as the most clinically relevant, being notably enriched in the blood of HF patients and exhibiting strong discriminatory power in distinguishing HF from non-HF individuals, including patients presenting with dyspnea. The AUC values ranged from 0.86 to 0.91 (*p* < 0.001), and elevated miR-423-5p levels were observed in both human and experimental rat models of systolic HF, with expression showing strong correlations with NT-proBNP concentrations and reduced ejection fraction.

Complementing these findings, the cardiac myocyte-enriched miR-499 was markedly elevated in patients with AHF compared to healthy controls. Importantly, its expression remained unaffected by potential confounding clinical variables such as age, sex, body mass index, renal function, systolic blood pressure, and white blood cell count [229] underscoring its specificity for cardiac injury and utility in AHF diagnosis. Similarly, the liver-enriched miR-122, while traditionally considered a marker of hepatic injury, was significantly elevated in AHF patients, probably reflecting hepatic venous congestion, a common complication of advanced HF [230]. In their study, Goren et al. [231] reported that serum levels of miR-423-5p, miR-320a, miR-22, and miR-92b were significantly increased in patients with systolic HF. These miRNAs correlated with key echocardiographic and clinical indicators, including elevated NT-proBNP levels, QRS complex widening, and left ventricular and atrial dilatation, underscoring their potential as diagnostic and phenotypic biomarkers in HF. Notably, circulating levels of miR-155 show heterogeneous behavior: they are reduced in chronic stable HF and associated with ventricular arrhythmias [232], yet trans-cardiac gradient data indicate release and net elevation of miR-155-5p from the failing myocardium, linking it to oxidative stress-driven decompensation [233].

Watson et al. [234] identified a signature of c-miRNAs—miR-30c, miR-146a, miR-221, miR-328, and miR-375—that, when assessed alongside BNP levels, significantly enhanced the ability to distinguish HFrEF from HFpEF, independent of echocardiographic findings. This combined approach improved diagnostic accuracy and effectively differentiated between the two HF subtypes.

In-depth analyses of miRNA expression in peripheral blood mononuclear cells (PBMCs) have revealed disease-specific expression profiles that distinguish between HF etiologies [235]. Notably, miR-107, miR-139, and miR-142-5p were consistently downregulated in both NIDCM and ICM [235]. In contrast, miR-142-3p and miR-29b were significantly upregulated in NIDCM, whereas miR-125b and miR-497 were specifically downregulated in ICM patients, reflecting distinct underlying pathophysiological mechanisms [235].

Muscle-enriched miR-133 demonstrated a strong inverse relationship with HF severity as classified by the New York Heart Association (NYHA) functional class. Its expression progressively declined with worsening clinical status, with patients having NT-proBNP levels >1800 pg/mL exhibiting a 25% reduction in circulating miR-133 levels compared with those with NT-proBNP <300 pg/mL, suggesting its role in disease monitoring and severity stratification [236].

Together the above suggests that c-miRNAs offer a promising and multifaceted approach for the diagnosis and phenotyping of HF. Their disease-specific expression patterns, strong correlations with established biomarkers and clinical features, and capacity to distinguish between HF subtypes position them as valuable additions to the current diagnostic arsenal. As research advances, integrating miRNA profiling with conventional assessments may significantly enhance diagnostic accuracy and personalized management for individuals with HF.

Advanced molecular profiling studies employing parallel miRNA and mRNA expression analyses have identified several differentially expressed c-miRNAs with enhanced diagnostic relevance [237]. In cases of end-stage HF, at least eight circulating miRNAs, including miR-30b, miR-103, miR-199a-3p, miR-23a, miR-27b, miR-324-5p, miR-342-3p, and miR-142-3p, exhibited significant alterations, further supporting their relevance for both diagnosis and disease staging [233].

Several microRNAs involved in the regulation of angiogenesis serve dual roles as mechanistic mediators and diagnostic biomarkers in HF. The endothelial-specific miR-126 was significantly downregulated in patients with congestive HF, exhibiting a negative correlation with age, logBNP levels, and NYHA class, suggesting its potential as a marker of endothelial dysfunction and disease severity [238]. In contrast, miR-210 and miR-30a were significantly upregulated in congestive HF patients compared to healthy controls and positively correlated with NT-proBNP concentrations. Notably, their expression was higher in patients with preserved ejection fraction (EF > 40%) than in those with reduced EF (<40%), highlighting their potential for HF subtype classification [239].

Recent meta-analytical approaches have provided comprehensive evaluations of miRNA diagnostic performance. Parvan et al. [240] systematically reviewed 45 studies to evaluate the diagnostic performance of circulating miRNAs for CHF. For HFrEF, an eight-miRNA panel—miR-18b-3p, miR-21-5p, miR-22-3p, miR-92b-3p, miR-129-5p, miR-320a-5p, miR-423-5p, and miR-675-5p—achieved a pooled sensitivity of 0.85, specificity of 0.88, and an AUC of 0.91. In contrast, for HFpEF, a seven-miRNA panel—miR-19b-3p, miR-30c-5p, miR-206, miR-221-3p, miR-328-5p, miR-375-3p, and miR-424-5p—demonstrated slightly lower diagnostic performance with sensitivity of 0.82, specificity of 0.61, and AUC of 0.79.

Further supporting the diagnostic promise of miRNAs, Kuai et al. [241] investigated their expression in high-risk hypertensive patients and identified 12 dysregulated miRNAs in those who developed HFrEF. A five-miRNA combination—miR-133a-3p, miR-378, miR-1-3p, miR-106b-5p, and miR-133b—achieved outstanding diagnostic performance with an AUC of 0.997, comparable to NT-proBNP. Bioinformatics analysis indicated that these miRNAs targeted 130 genes enriched in key cardiac signaling pathways, including MAPK, ErbB, and TGF-β. Similarly, Chen et al. [242] identified three novel miRNAs—miR-3135b, miR-3908, and miR-5571-5p—that were significantly elevated in HF patients. These miRNAs demonstrated AUC values of 1.00, 0.86, and 0.94, respectively, with miR-3135b and miR-3908 showing discriminatory power between HFrEF and HFpEF—an advantage not observed with NT-proBNP.

Collectively, these findings underscore the vast potential of c-miRNAs as diagnostic, phenotypic, and prognostic biomarkers in HF. Their tissue specificity, strong associations with established clinical parameters, and capacity to distinguish HF subtypes highlight their clinical relevance. Moreover, multi-miRNA panels and integrative approaches combining miRNAs with conventional biomarkers could substantially enhance precision in HF diagnosis and management. Future research should focus on standardizing miRNA detection methods, validating findings in larger and diverse populations, and integrating miRNA profiling into routine clinical workflows to fully harness their diagnostic and therapeutic potential.

### 5.2. Prognostic Value and Therapeutic Implications of c-microRNAs in Heart Failure

Beyond diagnostic applications, c-miRNAs demonstrate substantial prognostic value in HsF management. Seronde et al. [243] demonstrated that low admission levels of miR-423-5p in AHF patients were independently associated with increased risk of one-year hospital readmission and mortality. Patients in the lowest quartile of miR-423-5p expression had significantly poorer survival outcomes, supporting its role in risk stratification. In their study, Xiao et al. [244] examined the prognostic value of miR-30d in 96 patients with AHF over a one-year follow-up period. Lower circulating levels of miR-30d were significantly associated with increased all-cause mortality. Multivariate analysis identified miR-30d, alongside heart rate, hemoglobin, and serum sodium, as independent predictors of mortality. Notably, miR-30d demonstrated superior prognostic accuracy compared to hemoglobin and sodium (AUC = 0.806), with Kaplan–Meier survival analysis confirming that patients with higher miR-30d levels had significantly better survival outcomes (*p* = 0.001).

De Rosa et al. [245] analyzed transcoronary miRNA gradients in 75 patients undergoing coronary angiography, revealing etiology-specific release patterns. Patients with ischemic HF (ICM-HF) demonstrated significant cardiac release of miR-423 and miR-34a, while those with non-ischemic HF (NICM-HF) showed elevated transcoronary gradients for miR-21-3p and miR-30a. No gradients were observed for miR-126 or miR-199, suggesting distinct etiology-specific release patterns. These findings underscore the potential of transcoronary miRNA profiling as a heart-derived liquid biopsy to distinguish between HF etiologies.

Furthermore, c-miRNAs have demonstrated significant potential for predicting therapeutic responses in HF management. Sucharov et al. [246] investigated whether myocardial miRNA dynamics could predict β-blocker–induced reverse remodeling in idiopathic dilated cardiomyopathy (DCM). Endomyocardial biopsies from 43 DCM patients revealed that miR-208a-3p, miR-208b-3p, miR-21-5p, and miR-199a-5p were downregulated, while miR-1-3p was upregulated in therapy responders. These miRNAs were associated with pathways regulating apoptosis, hypertrophy, and contractility, supporting their utility as potential plasma biomarkers for treatment response prediction.

In a randomized controlled study nested within the GISSI-Heart Failure trial, Masson et al. [221] assessed c-miR-132 levels in 953 patients with chronic symptomatic HF. The association of c-miR-132 with adverse outcomes, including all-cause mortality, cardiovascular mortality, and HF-related hospitalizations, was evaluated. Elevated miR-132 levels were independently associated with several baseline features, including younger age, male sex, preserved renal function, ischemic HF etiology, and higher symptom burden. Although miR-132 was not independently associated with mortality after multivariate adjustment, it remained a significant predictor of HF-related rehospitalization (HR = 0.79; 95% CI: 0.66–0.95; *p* = 0.01). The addition of miR-132 to standard clinical risk models significantly improved predictive accuracy for hospitalization, with a continuous net reclassification improvement (cNRI) of 0.205 (*p* = 0.001).

Cardiac resynchronization therapy represents another area where miRNA biomarkers show promise for response prediction. Marfella et al. [247] examined 81 HF patients undergoing CRT and found that at 12 months, responders—defined by ≥15% increase in LVEF—exhibited significant upregulation of 19 miRNAs. Notably, miR-26b-5p, miR-145-5p, miR-92a-3p, miR-30e-5p, and miR-29a-3p were markedly elevated in responders versus non-responders (*p* < 0.01), suggesting a five-miRNA panel for predicting CRT benefit. Complementing these findings, Melman et al. [248] identified miR-30d as a strong predictor of CRT-induced reverse remodeling. In discovery and validation cohorts, higher baseline levels of miR-30d, especially from coronary sinus samples, predicted ≥10% improvement in LVEF. Functional studies confirmed that miR-30d is mechanically regulated and cardioprotective, targeting MAP3K4 and promoting adaptive cardiomyocyte growth.

In summary, the evolving landscape of c-miRNA research highlights their multifaceted value as powerful tools for the diagnosis, prognosis, and therapeutic monitoring of HF. By capturing nuanced molecular changes that precede and accompany clinical manifestations, c-miRNAs offer a level of specificity and sensitivity that surpasses many traditional biomarkers. Their proven associations with disease subtype, severity, treatment response, and long-term outcomes underscore their potential for guiding personalized HF management. As technological advances and validation studies continue to unfold, integrating c-miRNAs into routine clinical workflows holds the promise of transforming the standard of care, enabling earlier intervention and more tailored therapeutic strategies for patients with HF.

### 5.3. MicroRNAs in Acute Coronary Syndromes: Diagnostic Performance and Cardioprotective Mechanisms

C-miRNAs have also emerged as promising biomarkers in ACS, with potential utility for both early diagnosis and risk stratification. Specific miRNA expression patterns can help distinguish between ST-segment elevation myocardial infarction (STEMI) and non-STEMI (NSTEMI). For instance, miR-25-3p, miR-221-3p, and miR-374b-5p have been associated with STEMI, while miR-221-3p and miR-483-5p correlate more strongly with NSTEMI [249]. Cardiac-enriched miRNAs such as miR-1, miR-133a, miR-208b, and miR-499 are released within hours of symptom onset [250], offering a potential diagnostic advantage over traditional protein biomarkers. Among these, miR-499 stands out for its correlation with myocardial damage and its ability to differentiate ACS from stable coronary artery disease [251]. A recent plasma small RNA sequencing study identified 288 differentially expressed c-miRNAs in AMI patients compared to healthy controls, including 58 upregulated and 230 downregulated transcripts [252]. Beyond diagnosis, several miRNAs have prognostic value, such as miR-133a and miR-208; their plasma levels, measured six months post-AMI, were associated with increased all-cause mortality [253]. In this context, c-miRNAs not only serve as indicators of cardiac injury but also as markers of long-term outcomes.

Functionally, certain miRNAs play active roles in cardioprotection. For example, miR-21 has been shown to modulate inflammation and reduce peri-infarct apoptosis by suppressing inflammatory signaling pathways, reinforcing the dual role of miRNAs as biomarkers and therapeutic targets in ACS [254]. Several miRNAs, including miR-208b, miR-133a, miR-486, miR-150, and miR-21, are under investigation as novel diagnostic tools for AMI [255].

Emerging technologies, including neural network-based diagnostic models incorporating miRNA signatures, are also being developed to improve diagnostic precision across the ACS spectrum [256]. Although c-miRNAs show great promise, they have not yet replaced traditional biomarkers like cardiac troponins in clinical practice. Still, they represent an exciting frontier for improving diagnostic accuracy, risk stratification, and potentially guiding therapy in ACS.

### 5.4. MicroRNA Signatures in Heart Transplantation: Non-Invasive Biomarkers for Rejection and Graft Surveillance

Endomyocardial biopsy remains the gold standard for detecting acute cellular rejection (ACR) following heart transplantation [257]. However, its clinical utility is limited by procedural risks, sampling variability, and inter-observer inconsistency. Commonly used non-invasive blood biomarkers such as troponin and CRP lack the sensitivity and specificity required for early detection of rejection. In this context, c-miRNAs have emerged as promising non-invasive biomarkers for identifying ACR in heart transplant recipients [258].

In the immediate postoperative period after orthotopic heart transplantation, plasma levels of miR-133a, miR-133b, and miR-208a rise sharply within hours and decline over the subsequent two weeks. This kinetic profile closely mimics that of cTnI. Notably, miR-133b demonstrates a stronger correlation than troponin with key perioperative variables, including cardiopulmonary bypass time, central venous pressure (CVP), pulmonary capillary wedge pressure (PCWP), cardiac output (CO), ventilation time, and intensive care unit (ICU) stay duration. These findings suggest that miR-133b may serve as a more precise marker for detecting perioperative graft dysfunction [259]. The diagnostic utility of miRNA in this concept has also been supported by a multicenter Canadian study comparing 26 cases of ACR to 37 non-rejection controls. Seven miRNAs were significantly elevated during histologically confirmed rejection, with miR-142-3p (AUC = 0.78; 95% CI: 0.67–0.89) and miR-101-3p (AUC = 0.75; 95% CI: 0.62–0.87) demonstrating the highest discriminatory performance [260].

Beyond diagnostics, miRNA signatures offer mechanistic insights into I/R injury, a key contributor to graft dysfunction. In a mouse heterotopic heart transplant model, 20 miRNAs were upregulated and 39 downregulated in graft tissue compared to non-I/R controls. These dysregulated miRNAs were linked to pathways involved in apoptosis, inflammation, and tissue remodeling, highlighting both diagnostic and therapeutic potential [261].

Despite this promise, not all candidate miRNAs have translated successfully into clinical practice. A large prospective study involving 461 heart transplant recipients across 11 centers (831 biopsies, 79 rejections) evaluated a predefined panel of c-miRNAs (miR-10a, miR-92a, and miR-155). No significant associations were found between miRNA levels and biopsy-confirmed rejection, with odds ratios near 1 and *p*-values > 0.3. The trial was ultimately halted due to futility, underscoring the challenges of validating miRNA biomarkers at scale [262].

Among the currently studied candidates, miR-142-3p and miR-101-3p continue to show the most consistent performance in distinguishing ACR across independent cohorts [260]. However, major challenges remain, including standardizing pre-analytical workflows, validating expression kinetics over time, and integrating miRNA testing into routine post-transplant surveillance.

Future research should focus on validating combinatorial miRNA panels, developing point-of-care testing platforms, and correlating miRNA expression with long-term graft survival. Given their involvement in inflammation, fibrosis, and cell survival, miRNA-based therapies such as mimics or antagomirs are also under preclinical investigation as potential adjunctive interventions to mitigate rejection and promote myocardial recovery [263].

### 5.5. Therapy-Specific miRNA Shifts with Modern Disease-Modifying Drugs

Sodium-glucose cotransporter 2 (SGLT2) inhibitors and angiotensin receptor–neprilysin inhibitors (ARNIs) such as sacubitril/valsartan have become cornerstone therapies for HFrEF, offering significant improvements in morbidity and mortality [264,265]. Despite their widespread adoption, biomarkers capable of monitoring therapeutic response and guiding personalized treatment remain limited.

Recent studies have begun to reveal the potential of c-miRNAs as dynamic indicators of drug efficacy and underlying mechanisms of action. For instance, clinical data from Mone et al. [266] demonstrated that empagliflozin, an SGLT2 inhibitor, modulates miRNAs linked to endothelial dysfunction—a key pathophysiological feature in HFpEF. In frail older adults with HFpEF and diabetes mellitus, miR-21, and miR-92 were significantly upregulated compared to healthy controls, reflecting endothelial injury. Following three months of empagliflozin therapy, both miRNAs were markedly downregulated, suggesting restoration of endothelial function. Other miRNAs such as miR-126, miR-342-3p, and miR-638 were also dysregulated in HFpEF patients but remained unchanged with metformin or insulin, highlighting the drug-specific responsiveness of miR-21 and miR-92 to SGLT2 inhibition. Preclinical studies further support these findings. In a rat model of type 2 diabetes mellitus, Kiyak-Kirmaci et al. [267] compared the effects of empagliflozin and dapagliflozin on cardiac miRNA profiles. Empagliflozin significantly upregulated miR-146a and miR-34a, while dapagliflozin increased miR-146a but exerted distinct vascular effects in the thoracic aorta. Additional miRNAs—including miR-223, miR-373, miR-22, miR-9, miR-21, miR-144, and miR-221—were differentially expressed across treatment groups, pointing to divergent cardioprotective mechanisms between SGLT2 inhibitors.

ARNIs also modulate c-miRNAs in HF. In a study by Brioschi et al. [268], six months of sacubitril/valsartan therapy in HFrEF patients resulted in significant reductions in miR-29b-3p, miR-221-3p, and miR-503-5p, particularly in those with high baseline expression. These miRNAs were inversely correlated with peak VO_2_ (a measure of exercise capacity) and positively correlated with ST2 (a marker of inflammation and fibrosis). Bioinformatic analysis identified PIK3R1—a key regulator of PI3K signaling—as a common target of all three miRNAs, implicating a role in cardiac remodeling and endothelial function. Among these, miR-29b-3p is particularly noteworthy due to its established involvement in cardiac hypertrophy and dysfunction; its downregulation may reflect reverse remodeling in response to ARNI therapy.

Together, these findings underscore the emerging role of c-miRNAs as therapy-responsive biomarkers that not only reflect the molecular effects of disease-modifying drugs but also hold promise for guiding personalized treatment strategies in HF.

### 5.6. Diagnostic Potential of microRNAs in Heart Failure

Beyond their diagnostic and prognostic utility, miRNAs represent a novel class of therapeutic agents [269]. Two major strategies have been proposed: miRNA inhibition using antisense oligonucleotides (anti-miRs) [270], and miRNA replacement therapy using synthetic miRNA mimics [271]. Anti-miRs are designed to silence pathologically upregulated miRNAs, thereby restoring the expression of target proteins. These molecules have demonstrated efficient systemic delivery, tissue uptake, and minimal toxicity in preclinical models. Conversely, miRNA mimics aim to restore the function of downregulated miRNAs, suppressing aberrant protein expression and mitigating disease progression. The clinical interest in miRNA-based therapies is growing. A notable example is CDR132L, a locked nucleic acid (LNA)-based antisense inhibitor targeting miR-132-3p, which has shown promising first-in-human results in HF patients [272]. This study marks a critical step toward miRNA-targeted interventions in CVDs. Likewise, inhibition of miR-92a has been associated with enhanced neovascularization and reduced infarct size in ischemia models [273].

Notably, miRNAs are uniquely suited to modulate complex disease pathways, as a single miRNA can simultaneously regulate multiple mRNAs across interrelated biological processes [274]. This pleiotropic regulatory capacity makes them particularly attractive for therapeutic targeting in HF, where dysregulated gene networks contribute to disease progression. Furthermore, targeted delivery strategies such as exosome-based transport or catheter-guided administration have shown promise in animal models [31]. As also highlighted above emerging evidence suggests that miRNA profiles obtained through LB techniques can identify HF onset, monitor therapeutic response, and even detect early allograft rejection in transplant recipients.

Taken together, these findings highlight that, due to their ability to regulate multiple gene networks, miRNAs are emerging as valuable “theranostic” tools serving both therapeutic and diagnostic purposes in the field of precision cardiology [220]. Yet, despite these advances, significant challenges remain before miRNAs can be widely used as therapeutic targets in CVDs including HF. These include gaps in our understanding of their pharmacokinetics, concerns about potential off-target effects, and limitations in delivery methods.

## 6. Integrating Circulating miRNAs with Protein Biomarkers in Liquid Biopsies for Heart Failure Diagnosis

### 6.1. The Concept of Multi-Analyte Liquid Biopsy for Heart Failure Management

As discussed above, HF management currently relies heavily on established protein biomarkers in addition to imaging techniques. These biomarkers reflect distinct pathophysiological processes and NPs, Gal-3, sST2, and troponins. While these established biomarkers have proven clinical utility, they primarily capture end-stage pathophysiological processes and may lack the sensitivity needed for early detection or comprehensive disease characterization. The limitations of conventional protein biomarkers have prompted exploration of alternative molecular signatures that may provide earlier and more comprehensive disease insights. As extensively discussed in previous sections, c-miRNAs represent a particularly promising biomarker class for HF applications. Unlike traditional protein biomarkers that reflect downstream effects of disease processes, c-miRNAs are functional regulators that directly participate in cardiac pathophysiology.

**Figure 2:**
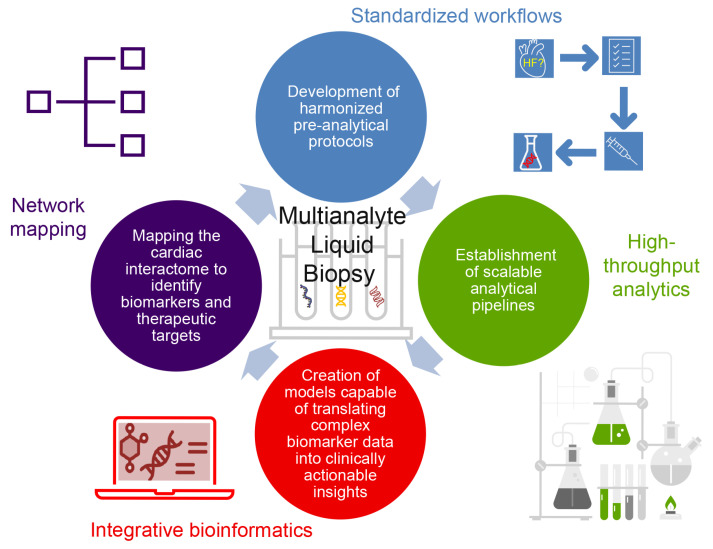
The essential components required to establish robust multi-analyte liquid biopsy platforms for heart failure, combining methodological standardization, analytical scalability, systems biology, and data integration.

Our recent review [275] demonstrated that multi-analyte approaches, particularly those combining cfDNA, miRNAs, and specific protein markers, offer superior diagnostic and prognostic accuracy compared to traditional single-marker tests. However, most multi-analyte LB studies have focused on oncology, leaving analogous research in cardiovascular diseases relatively scarce. Understanding the relationships among prognostic biomarkers of myocardial stress, injury, inflammation, and fibrosis, such as NT-proBNP, troponin, soluble urokinase-type plasminogen activator receptor (suPAR), and Gal-3, can elucidate mechanisms of HF pathogenesis, adverse ventricular remodeling, and key pathways important to disease progression [276]. Successful implementation of multi-analyte liquid biopsy platforms in HF depends on several critical pillars. Figure 2 outlines these essential components, which include methodological standardization, scalable analytical pipelines, systems biology approaches for network mapping, and integrative bioinformatics for translating complex data into clinical insights.

Overall, multi-analyte LB platforms that integrate c-miRNAs with protein biomarkers represent a transformative strategy for early diagnosis, refined phenotyping, and personalized HF management. Realizing this potential requires systematic biomarker discovery, standardized methodologies, and large-scale validation across diverse cohorts. Given the molecular heterogeneity of HF and the early onset of molecular changes before clinical symptoms, network-informed biomarker combinations could enable earlier interventions and improve long-term outcomes.

### 6.2. Combining c-miRNAs with Soluble Protein Biomarkers for Heart Failure Management: Success Stories

Recent studies have demonstrated the clinical utility of combining c-miRNAs with established protein biomarkersin HF. In addition to their individual diagnostic and prognostic capabilities, the integration of c-miRNAs with established clinical biomarkers including NT-proBNP, Gal-3, sST2, and hs-cTn represents a promising strategy for enhancing clinical utility while mitigating inter-platform variability. Multi-analyte approaches capitalize on complementary biological pathways and may simultaneously address critical limitations inherent to standalone miRNA assays, including analytical variability, absence of standardized protocols, and insufficient clinical validation studies. This integrated biomarker strategy potentially provides a more robust and clinically actionable molecular signature than individual markers alone. Furthermore, the complementary information provided by c-miRNAs (post-transcriptional regulation) and protein biomarkers (functional state) can enhance clinical decision-making and support ongoing standardization and validation efforts [277].

Importantly, standardization efforts in LB for oncology provide a valuable framework that can be adapted to cardiovascular applications, including HF. The Blood Profiling Atlas in Cancer (BloodPAC) Consortium has developed FDA-reviewed validation protocols and open-access standard operating procedures (SOPs) encompassing pre-analytical sample handling, cfDNA and miRNA extraction, and inter-laboratory ring-trial methodology for LB assays [278]. In addition, the European Liquid Biopsy Society (ELBS) facilitates multi-center benchmarking and external quality assessment programs that standardize sample processing and result reporting across multiple laboratories [279]. Implementing these established collaborative frameworks in HF research could expedite the clinical validation of integrated circulating miRNA and protein biomarker panels. From an analytical perspective, rigorous implementation of the updated MIQE 2.0 guidelines for reverse transcription quantitative PCR (RT-qPCR) and digital PCR methodologies, combined with National Institute of Standards and Technology (NIST) Standard Reference Materials and synthetic spike-in controls, can reduce inter-assay variability and facilitate cross-platform calibration of miRNA quantification [280]. Integration of these quality control frameworks into prospective HF cohort studies will enhance analytical reproducibility while facilitating regulatory approval pathways for multi-analyte liquid biopsy diagnostic platforms.

**Figure 3:**
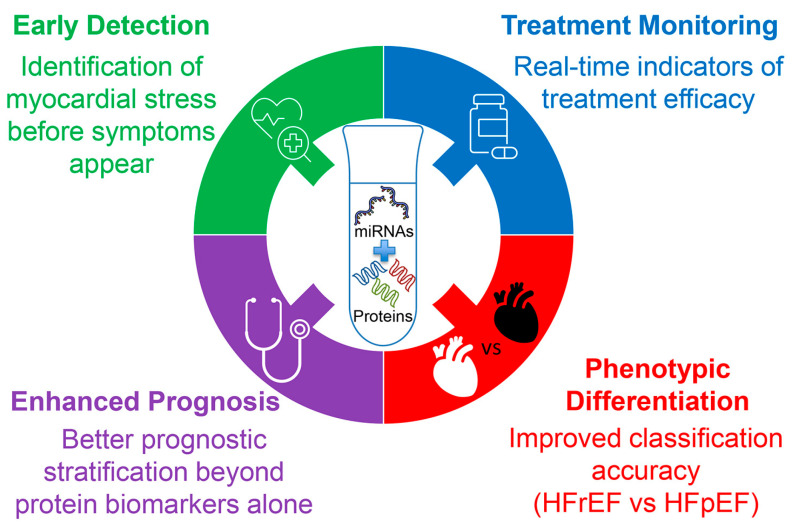
Clinical benefits of combining circulating miRNAs with protein biomarkers in heart failure management. This multi-analyte strategy supports early detection, more accurate phenotypic differentiation, enhanced prognostic stratification, and real-time treatment monitoring.

As summarized in Figure 3, such multi-analyte biomarker panels can enhance early detection, improve phenotypic classification, sharpen prognostic assessment, and support more precise treatment monitoring.

The following sections review key representative studies demonstrating the added value of combining c-miRNAs with conventional biomarkers, underscoring their potential to improve the accuracy of HF diagnosis, prognosis, and therapeutic response assessment.

#### 6.2.1. Integrating MicroRNAs with Natriuretic Peptides for Enhanced Heart Failure Management

To improve diagnostic and prognostic performance in HF, a multi-marker strategy that combines c-miRNAs with NPs is gaining attention. This approach leverages the complementary strengths of each biomarker class: miRNAs provide insights into post-transcriptional regulatory mechanisms involved in cardiac remodeling and stress responses, while NPs, such as BNP and NT-proBNP, reflect hemodynamic stress and ventricular dysfunction. Recent studies suggest that integrating miRNAs with conventional HF biomarkers may enhance the accuracy of both diagnosis and prognosis (Table 9).

In a combined pre-clinical/clinical investigation, Endo et al. [281], first demonstrated in Dahl salt-sensitive rats that plasma miR-210 increases approximately 15-fold after eight weeks of high-salt feeding and correlates tightly with BNP (r^2^ ≈ 0.75). Translating the finding to humans, they measured baseline plasma miR-210 in 39 out-patients with NYHA II HF and re-checked BNP three months later. Although miR-210 and BNP were not associated cross-sectionally, every patient whose BNP fell on follow-up belonged to the low-miR-210 subgroup (0.42 ± 0.10 vs. 0.65 ± 0.25 in non-improvers; *p* < 0.05). These data indicate that circulating miR-210, a hypoxia-responsive transcript mainly released from skeletal muscle and blood mononuclear cells, captures peripheral oxygen-supply mismatch rather than ventricular wall stress. Consequently, low baseline miR-210 can flag patients poised for favorable BNP trajectories, positioning miR-210 as a complementary—rather than redundant—partner to BNP in CHF monitoring.

Ellis et al. [282] investigated whether c-miRNAs could complement traditional protein biomarkers to improve diagnostic accuracy for HF in patients presenting with dyspnea. In a validation cohort of 150 individuals, including 44 with HF, 32 with chronic obstructive pulmonary disease (COPD), 59 with other causes of dyspnea, and 15 healthy controls, the authors quantified 17 candidate miRNAs and compared their diagnostic performance with that of established biomarkers: NT-proBNP (AUC = 0.896) and high-sensitivity troponin T (AUC = 0.750). Among the miRNAs studied, four—miR-103, miR-142-3p, miR-30b, and miR-342-3p—were significantly downregulated in HF patients (*p* < 0.03), although their individual diagnostic performance was modest (AUCs ranging from 0.621 to 0.668). While NT-proBNP alone outperformed any single miRNA, combining miR-423-5p with NT-proBNP improved the AUC by 3.2% (*p* = 0.030). More notably, integrating all significantly dysregulated miRNAs with NT-proBNP resulted in a 4.6% improvement in diagnostic accuracy (*p* = 0.013). These findings demonstrate that select circulating miRNAs, when used in conjunction with traditional biomarkers, can meaningfully enhance HF diagnosis in clinically ambiguous presentations such as dyspnea.

Expanding on the application of miRNA profiling in HF subtyping, Watson et al. [234] examined whether circulating miRNA signatures could differentiate between HFpEF and HFrEF, independent of imaging modalities such as echocardiography. Their analysis among non-HF, HFrEF, and HFpEF identified five miRNAs (miR-30c, miR-146a, miR-221, miR-328, and miR-375) that were differentially expressed between HFpEF and HFrEF, as well as between HF and non-HF patients. These findings were validated in an independent cohort of 225 individuals. Importantly, the combination of this five-miRNA panel with BNP significantly improved diagnostic accuracy for distinguishing HF subtypes, achieving an AUC exceeding 0.82. This study was among the first to demonstrate the feasibility and clinical value of multi-marker approaches combining miRNAs and NPs.

Wong et al. [283] conducted a comprehensive study to evaluate the diagnostic utility of c-miRNAs in HF, with a specific focus on differentiating between HFrEF and HFpEF. The study employed a two-cohort design involving a total of 176 participants. Through differential expression analysis, the authors identified 12 miRNAs with potential diagnostic relevance. Among these, miR-125a-5p, miR-550a-5p, and miR-638 were significantly upregulated in HFrEF compared to HFpEF, while miR-190a was downregulated, facilitating subtype discrimination. ROC analysis demonstrated that although individual miRNAs exhibited moderate diagnostic performance, combining them into a multi-miRNA panel markedly improved classification accuracy. The panel achieved an AUC of 0.80 for distinguishing between HFrEF and HFpEF. Crucially, when this miRNA panel was integrated with NT-proBNP, the diagnostic performance increased further, achieving perfect discrimination (AUC = 1.00) between the two HF phenotypes in some comparisons. This highlights the added value of multi-marker strategies in refining HF diagnosis. Additionally, pathway enrichment analysis revealed that the dysregulated miRNAs were associated with key molecular pathways involved in HF pathogenesis, including Wnt signaling, p53 regulation, Toll-like receptor signaling, and the PI3K-Akt pathway. These findings not only reinforce the diagnostic potential of circulating miRNAs but also suggest mechanistic links to the molecular biology of HF subtype.

Expanding on this multi-marker concept, Yan et al. [284] conducted a systematic review and meta-analysis involving 10 studies to evaluate the diagnostic performance of circulating miRNAs alone and in combination with BNP for HF detection. The analysis included 33 mixed-miRNA tests, four miR-423-5p-only tests, 11 mixed-miRNA + BNP tests, and three BNP-only tests. Pooled results for mixed-miRNA panels yielded a sensitivity of 0.74 (95% CI: 0.72–0.75), specificity of 0.69 (95% CI: 0.67–0.71), and a summary receiver operating characteristic (SROC) AUC of 0.7991. When evaluated independently, miR-423-5p demonstrated higher accuracy, with a sensitivity of 0.81, specificity of 0.67, and an AUC of 0.8600. However, BNP alone outperformed all miRNA-only strategies, achieving a sensitivity of 0.70, specificity of 0.80, and AUC of 0.9291. Critically, the combination of mixed-miRNA panels with BNP improved diagnostic accuracy significantly, achieving a sensitivity of 0.85, specificity of 0.81, and an AUC of 0.9146.

Another study by Wong et al. [285] identified specific circulating microRNA signatures that can accurately detect nonacute HF and differentiate between HFpEF and HFrEF—areas where clinical assessments and natriuretic peptide tests often fall short. In detail, in the discovery cohort of 546 Singaporean participants, an eight-miRNA panel achieved an AUC of 0.96, specificity of 0.88, and overall accuracy of 0.89. These impressive results were confirmed in two independent validation cohorts, yielding AUCs of 0.88 and 0.87, respectively. When combined with NT-proBNP, diagnostic accuracy improved further: the discovery cohort achieved an AUC of 0.99 (versus 0.96 with NT-proBNP alone), and Validation 1 reached 0.97 (versus 0.96 alone). Additionally, integration of the miRNA panel elevated subtype classification performance, increasing AUC from 0.71 (NT-proBNP alone) to 0.87 in the discovery cohort. Notably, the combined biomarkers correctly reclassified 72% of NT-proBNP false negatives in the Singapore cohort and 88% in the New Zealand cohort, most of whom had HFpEF, demonstrating the panel’s potential to significantly enhance the diagnosis and phenotyping of nonacute HF.

Together, the above suggest that integrating c-miRNAs with traditional HF biomarkers such as NPs holds strong potential for improving diagnostic accuracy, phenotypic differentiation, and risk stratification in HF. Multi-marker approaches capture the molecular complexity underlying diverse HF phenotypes and help address limitations of individual biomarkers. As validation studies grow, these strategies may enable more precise, earlier, and personalized care in HF.

#### 6.2.2. Beyond Natriuretic Peptides: Emerging Multi-Marker Models Incorporating MicroRNAs for Prognostic Enhancement

Beyond NPs, recent research has increasingly focused on integrating circulating microRNAs with other soluble biomarkers such as Gal-3, sST2, hs-TnI/T to further refine HF diagnosis and prognostication. This multi-marker strategy leverages the complementary biological pathways captured by these biomarkers, encompassing myocardial stress, fibrosis, inflammation, and cardiomyocyte injury. By combining these diverse molecular signals, emerging models aim to improve risk stratification and therapeutic decision-making in both HFrEF and HFpEF populations.

Key studies supporting these multi-analyte approaches are summarized in Table 10 and discussed in detail below, highlighting the growing evidence for the clinical utility of combined biomarker panels in HF management.

In a recent prospective cohort study, Spahillari et al. [276] enrolled 139 patients with symptomatic HF to determine whether circulating plasma miRNA profiles enhance characterization of cardiac structure, function, and clinical outcomes beyond established biomarkers. The authors quantified 32 miRNAs and measured NT-proBNP, hs-TnI, suPAR, and Gal-3, then correlated these with echocardiographic indices and a composite endpoint of HF hospitalization, LVAD implantation, transplant, or death. Principal component analysis reduced the miRNA data into five components, of which PC2, PC3, and PC4 were independently linked to left ventricular ejection fraction, chamber volumes, and right ventricular systolic function, respectively. Importantly, incorporating PC3 and PC4 into risk models alongside conventional biomarkers increased concordance from 0.75 to 0.82, demonstrating the incremental prognostic value of miRNA signatures. Moreover, the study provided novel evidence that miRNA principal components, especially when integrated with Gal-3, offer prognostic information independent of natriuretic peptides. This combined biomarker approach significantly enhances risk stratification in HF and underscores the emerging role of non-coding RNAs in clinical phenotyping

Al-Hayali et al. [286] valuated the diagnostic value of circulating miR-1 and miR-21 for detecting AHF and silent coronary artery disease (SCAD) in asymptomatic type 2 diabetes mellitus (T2DM) patients, and explored their associations with NT-proBNP and Gal-3. The study included 135 T2DM patients divided into three groups—diabetic (DM), DM + SCAD, and DM + AHF—and 45 healthy controls. They found that miR-1 was progressively downregulated across the diabetic groups: 0.54-fold in DM and DM + SCAD, dropping to just 0.12-fold in the DM + HF group compared to controls (*p* < 0.001) In contrast, miR-21 was upregulated—1.30-fold in DM, 1.79-fold in DM + SCAD, and 2.21-fold in DM + HF—reaching significantly higher levels in HF versus SCAD (*p* < 0.001). Importantly, in the DM + AHF group, miR-1 showed strong negative correlations with both NT-proBNP (r = −0.891) and Gal-3 (r = −0.886), while miR-21 showed strong positive correlations (r = 0.734 and r = 0.764, respectively; all *p* < 0.001) These results suggest that decreased miR-1 and increased miR-21 levels reflect elevated NT-proBNP and Gal-3 in AHF. The authors emphasized that miR-21, in particular, may outperform miR-1 in predicting AHF in asymptomatic T2DM and that a biomarker panel combining miR-21 with NT-proBNP and Gal-3 provides enhanced predictive accuracy. These findings support using miR-21 alongside established protein markers to improve early HF detection in T2DM patients. However, larger prospective studies are needed to confirm whether such miRNA–protein biomarker panels can effectively guide risk stratification and personalized HF management in this high-risk population.

The correlation of miR-1, miR-21, with serum Gal-3 has also been investigated in patients presenting with symptomatic HF, left ventricular hypertrophy, and clinically documented arterial hypertension [287]. It was shown that reductions in miR-1 and miR-21 expression occurred in patients with HFa, and these results were significantly correlated with the concentration of serum Gal-3, the important factor playing the key role in the fibrotic process.

In the study by Han et al. [288] the clinical relevance of miR-214 and serum Gal-3 levels was evaluated in patients with CHF. The study included 50 CHF patients and 30 age- and sex-matched healthy controls. MiR-214 expression and Gal-3 concentration were determined, both at baseline and after six months of standard therapy. At baseline, CHF patients exhibited significantly higher levels of miR-214, and Gal-3 compared to controls (*p* < 0.001), with a strong positive correlation between them (r = 0.712, *p* < 0.05). ROC analysis showed good diagnostic performance, with AUC values of 0.916 for miR-214 and 0.852 for Gal-3. Both markers declined significantly following treatment, and their pre-treatment levels predicted clinical response with AUCs of 0.874 and 0.897, respectively. These findings suggest that miR-214 and Gal-3 may act synergistically in CHF pathogenesis. Gal-3 promotes myocardial fibrosis, while miR-214 potentially regulates fibroblast proliferation. Together, they represent promising complementary biomarkers for CHF diagnosis, prognosis, and treatment monitoring.

Several c-miRNAs (miR-21, miR-208b, miR-499, miR-132, miR-423-5p) have myocardial injury dynamics similar to troponin. Studies show these miRNAs correlate with troponin and can enhance diagnostic and prognostic models in HF. However, few direct clinical studies simultaneously measure miRNA and troponin in HF patients—indicating a gap for future research. For example, Zhang et al. [289] investigated the relationship between circulating miR-145 and cardiac troponin levels in 100 patients undergoing coronary angiography. They observed that lower plasma miR-145 levels were strongly and inversely correlated with peak troponin I (Spearman ρ = −0.62; *p* < 0.0001), suggesting that miR-145 may reflect infarct size and the extent of cardiomyocyte injury. In the same study, plasma miR-21 was significantly elevated in patients with AMI compared to those with angina or healthy controls and showed a positive correlation with peak cTnI and CK-MB levels.

Masson et al. [221] evaluated whether circulating miR-132 predicted outcomes in CHF by measuring plasma miR-132 at randomization in 953 GISSI-HF trial participants and relating it to demographics, NT-proBNP, hs-cTnT, hs-CRP, pentraxin-3, and long-term events (all-cause/CV mortality and first CV/HF hospitalization) over 46 months. They found that lower baseline miR-132 levels were independently associated with worse renal function, higher NYHA class, ischaemic etiology; in multivariable Cox models, even after adjustment for NT-proBNP, they predicted HF hospitalizations (HR 0.79 per log unit; *p* = 0.01) but not mortality. Adding miR-132 to clinical risk factors plus NT-proBNP improved continuous net reclassification for HF admission (cNRI 0.205; *p* = 0.001). MiR-132 also inversely correlated with NT-proBNP and hs-cTnT, underscoring its potential as a complementary biomarker to refine risk stratification for HF-related rehospitalization.

To the best of our knowledge, studies examining c-miRNAs with sST2 remain limited. Only a single clinical study by Arul et al. [290] directly examined circulating miR-210-3p alongside sST2 and confirmed their correlation in HF patients. In this cross-sectional study of 270 hypertensive HF patients (170 with HFpEF, 100 with HFrEF) and 20 healthy controls, the authors compared miR-210-3p levels with NT-proBNP, sST2, and Gal-3 across HF phenotypes. All four biomarkers were significantly higher in HFrEF compared to HFpEF (*p* ≤ 0.03). Notably, miR-210-3p showed an 11.9-fold increase in HFrEF versus only a 1.08-fold increase in HFpEF, achieving an AUC of 0.79 (87% sensitivity, 54% specificity) at a cut-off value of 5.03. Additionally, miR-210-3p levels correlated positively with Gal-3, NT-proBNP, sST2, and the MAGGIC (Meta-Analysis Global Group in CHF) risk score (r ≥ 0.52, *p* < 0.001). While the MAGGIC score alone outperformed individual biomarkers (AUC 0.86), miR-210-3p provided independent diagnostic value and emerged as a promising marker for early detection and phenotypic differentiation in HF. These findings highlight the potential of integrating miR-210-3p into multi-marker panels to enhance HF characterization and risk stratification.

However, there remains a critical research gap in validating comprehensive models that combine miRNA profiles with sST2 and other protein biomarkers. Prospective cohort studies are warranted to determine whether such combined approaches, independent of NT-proBNP and Gal-3, can improve prognostic accuracy and guide personalized therapy in HF.

## 7. Limitations in Employing miRNAs in Clinical Practice

Although substantial evidence supports the diagnostic and prognostic potential of c-miRNAs in HF, several challenges still limit their routine clinical application. In our recent review, we outlined the key factors affecting c-miRNA profiles and concentrations when evaluated as biomarkers for CVDs, including HF [291]. These limitations are briefly summarized below. We emphasized the need for harmonizing technical procedures, including selecting the appropriate blood fraction, anticoagulant, centrifugation protocol, and storage conditions, to enhance the reproducibility and clinical utility of c-miRNA profiling across CVDs and other clinical contexts.

The absence of standardization across pre-analytical, analytical, and post-analytical phases remains a fundamental barrier to the clinical translation of c-miRNA biomarkers. Variables such as the biological source (whole blood, serum, plasma), type of anticoagulant, blood collection tubes, centrifugation protocols, storage conditions, and biomarker isolation and quantification techniques can all significantly affect data quality and reproducibility [261,262]. These technical inconsistencies undermine inter-study comparability and compromise the reliability of c-miRNA measurements across laboratories. Harmonizing these procedures is therefore critical for ensuring robust and reproducible findings, without which the clinical utility of c-miRNAs in cardiovascular diseases and other pathologies will remain limited.

Another issue is that c-miRNA levels are influenced by numerous biological and environmental factors, including age, sex, physical activity, medication use, and dietary habits, which further complicate data interpretation [292]. These confounding variables introduce additional layers of complexity that must be carefully controlled in clinical studies to ensure accurate and meaningful results. The multifactorial nature of c-miRNA regulation necessitates comprehensive patient phenotyping and standardized protocols for sample collection and processing.

A persistent challenge in translating c-miRNAs into clinical practice is the substantial methodological heterogeneity across studies, particularly in HFand atrial fibrillation research [293,294,295]. Variability in RNA extraction protocols, inconsistent internal reference controls, and the use of diverse analytical platforms have led to poor reproducibility and limited cross-study comparability. The lack of consensus on normalization strategies, whether using synthetic spike-in controls, endogenous miRNAs, or global mean normalization, further complicates data interpretation and benchmarking [291,295].

Beyond these methodological inconsistencies, technical barriers remain. RNA isolation from biological fluids is inherently challenging, compounded by the absence of universally accepted endogenous controls. Processing large clinical sample sets is also hindered by the high cost, procedural complexity, and need for specialized equipment and trained personnel. Although c-miRNAs exhibit relative stability compared to other RNA species, their integrity can still be affected by factors such as prolonged storage, repeated freeze-thaw cycles, and suboptimal handling conditions. Standardizing these pre-analytical variables is essential to ensure reliable and reproducible c-miRNA analysis in clinical settings [291,295].

Additionally, most studies have relied on small, single-center cohorts, which limits both the generalizability and statistical power of their findings. The absence of validation in independent, large-scale cohorts has contributed to ongoing skepticism regarding the clinical utility of c-miRNAs as reliable biomarkers for HF diagnosis and patient stratification. This limitation is particularly problematic given the heterogeneous nature of HF, which encompasses multiple phenotypes with distinct pathophysiological mechanisms.

The lack of longitudinal studies tracking c-miRNA changes over time and their relationship to clinical outcomes further limits our understanding of their prognostic value. Many studies have focused on cross-sectional analyses, which provide limited insight into the dynamic nature of miRNA expression during disease progression.

To overcome these limitations and facilitate successful clinical translation, rigorous study design is essential. This includes appropriate power and sample size calculations, harmonized methodologies, and multi-center validation studies [295]. Specifically, future research should prioritize the following:

- Standardization initiatives: Development of consensus protocols for sample collection, processing, and analysis;
- Validation studies: Large-scale, multi-center studies with independent validation cohorts;
- Longitudinal assessments: Prospective studies tracking c-miRNA changes over time;
- Cost-effectiveness analysis: Economic evaluations to support clinical implementation;
- Regulatory considerations: Alignment with regulatory requirements for biomarker qualification.

Addressing these methodological and technical gaps will be critical for the successful clinical translation of miRNA-based diagnostics in HF and related CVDs. Only through systematic resolution of these challenges can c-miRNAs fulfill their potential as clinically useful biomarkers for HF management.

## 8. Perspectives and Recommendations

HF represents a multifaceted clinical syndrome characterized by complex molecular processes, including inflammation, fibrosis, hypertrophy, apoptosis, and metabolic dysfunction. While traditional soluble biomarkers such as NPs, troponins, Gal-3, and sST2 have contributed significantly to HF diagnosis and prognosis, they remain insufficient for providing phenotypic precision and prognostic stratification across diverse clinical contexts. C-miRNAs have emerged as powerful, non-invasive biomarkers that reflect the dynamic interplay of molecular pathways driving HF progression. Their tissue specificity, remarkable stability, and regulatory functions make them ideal candidates for both biomarker discovery and therapeutic targeting. Specific miRNAs such as miR-21, miR-133, miR-155, miR-423-5p, and miR-29 are increasingly recognized for their roles in cardiac remodeling, immune modulation, and myocardial stress responses. The unique properties of c-miRNAs include the following:

• Mechanistic relevance: Direct involvement in cardiomyocyte apoptosis, fibrosis, and remodeling pathways;
• Early detection potential: Expression changes occur before clinical manifestations;
• Phenotypic specificity: Distinct expression patterns associated with different HF subtypes;
• Therapeutic implications: Potential targets for RNA-based interventions.

The integration of multiple circulating biomarker classes including microRNAs, cf DNA, proteins, and extracellular vesicle-derived RNAs, into unified LB platforms holds considerable promise for advancing HF diagnosis, prognosis, and phenotypic stratification. Compared with single-marker strategies, multi-analyte approaches offer a more comprehensive and dynamic molecular portrait of disease, capturing genetic, epigenetic, and functional alterations across distinct HF subtypes, as discussed in the following sections.

### 8.1. Advancing Heart Failure Diagnosis with Multi-Analyte Liquid Biopsy: Demographic Considerations and Emerging Biomarkers

As discussed above, recent evidence supports the clinical utility of combining c-miRNAs with NPs, Gal-3, sST2, troponins, and other protein biomarkers to enhance diagnostic precision and risk prediction in HF. This multi-marker strategy is particularly valuable for distinguishing HFrEF from HFpEF and for forecasting adverse outcomes. However, despite these promising insights, large-scale, prospective studies rigorously evaluating the additive value of such multimodal biomarker panels across diverse HF populations remain limited. This need is further underscored by accumulating evidence that biomarker levels vary significantly across patient subgroups [296], making demographic- and phenotype-specific validation a critical prerequisite for the clinical translation of c-miRNA panels and other biomarkers, in HF. Ethnic disparities in cardiovascular biomarker expression are well documented, with population-level differences observed in NP concentrations, troponin thresholds, and systemic inflammatory markers—all of which influence diagnostic sensitivity and specificity [297,298]. Age-related alterations in c-miRNA expression further complicate interpretation, as aging affects miRNA biogenesis, degradation pathways, and cellular stress responses, while also introducing age-specific comorbidity patterns that may confound biomarker reliability [299]. Sex-based differences in cardiovascular biology present another critical variable [300]. Hormonal influences on miRNA expression and sex-specific disease trajectories necessitate careful consideration to ensure diagnostic and prognostic consistency across male and female patients [301]. In parallel, phenotype-specific validation is essential, as HFpEF, HFrEF, and HFmrEF represent distinct pathophysiological entities with divergent molecular drivers, prognoses, and therapeutic responses [302]. Notably, HFpEF disproportionately affects older adults, women, and certain ethnic minorities, highlighting the intersection between demographic diversity and HF subtypes [303]. Overlooking these differences may compromise the clinical utility and generalizability of c-miRNA-based diagnostics. To ensure equitable clinical translation, future multi-center studies must incorporate stratified analyses across ethnicity, age, sex, and HF phenotype [303]. This approach will be essential for developing robust, population-sensitive biomarker platforms that advance precision cardiology while minimizing the risk of reinforcing existing healthcare disparities.

**Figure 4:**
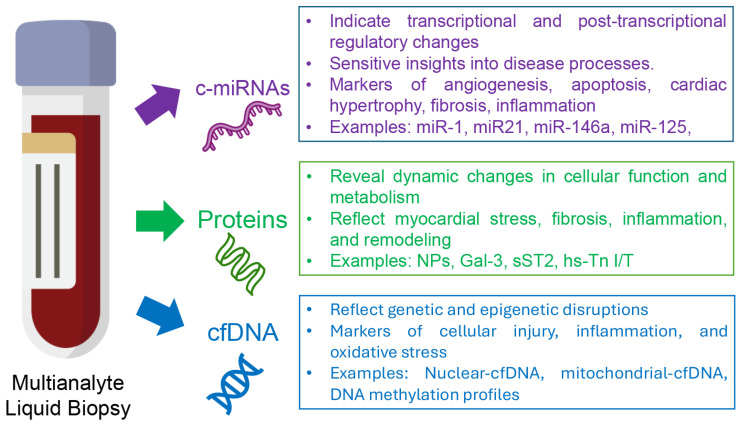
The concept of multi-analyte liquid biopsy framework for comprehensive heart failure management. This framework integrates circulating microRNAs (c-miRNAs), proteins, and cell-free DNA (cfDNA), each capturing distinct yet complementary biological processes in HF. Together, they have the potential to provide a multidimensional approach to enhance diagnosis, prognosis, and treatment monitoring.

Moreover, although the pathophysiological relevance of cfDNA in HF—particularly its role in reflecting genomic instability, cellular injury, and inflammation—is increasingly recognized, dedicated studies evaluating cfDNA as a clinical biomarker in HF are still scarce [304,305]. This gap underscores the need for more comprehensive biomarker strategies. Given the distinct and complementary insights provided by different biomarker classes, we propose a comprehensive multi-analyte approach that integrates c-miRNAs, cfDNA, and protein biomarkers (Figure 4).

As illustrated in Figure 4, c-miRNAs capture transcriptional and post-transcriptional regulatory changes, offering sensitive insights into disease processes. Cell-free DNA reflects genetic and epigenetic alterations, serving as a marker of cellular damage and genomic instability. Circulating proteins indicate dynamic changes in cellular function and metabolism, encompassing established HF biomarkers such as NPs, Gal-3, sST2, and troponins, along with emerging protein markers like cardiac myosin-binding protein C and growth differentiation factor-15 (GDF-15). Together, these biomarker classes offer a multidimensional perspective on HF pathophysiology, with the potential to advance diagnostic, prognostic, and therapeutic strategies. Notably, multi-analyte LB approaches offer substantial advantages over traditional single-analyte strategies by capturing the complex, multidimensional nature of disease processes. Such platforms deliver a high-resolution molecular snapshot of disease status, leveraging the complementary diagnostic contributions of each biomarker class [306,307].

Recent advances have expanded the spectrum of LB analytes beyond cfDNA and miRNAs for HF management. Of particular interest are cardiac-derived EVs, sometimes referred to as “cardiovesicles,” which are continuously released into circulation and carry a cargo of RNA and protein that reflects the functional state of the myocardium. Notably, Gokulnath et al. [308] investigated the potential of plasma EV-derived RNA profiles as a LB tool to differentiate between subtypes of acute decompensated HF (ADHF), specifically HFpEF and HFrEF. Utilizing RNA sequencing on EVs isolated from plasma samples of 214 patients, the study identified distinct transcriptomic signatures associated with each HF subtype. Principal component analysis and differential expression profiling revealed that HFrEF-associated transcripts were predominantly cardiomyocyte-derived, with enrichment in pathways related to dilated and hypertrophic cardiomyopathy. In contrast, HFpEF transcripts reflected more systemic origins, implicating biological processes such as mitochondrial metabolism and endoplasmic reticulum stress. These findings were subsequently validated in an independent cohort. Importantly, the study demonstrated that lncRNAs and mRNAs packaged in EVs are reliably detectable in plasma, offering a novel and minimally invasive approach for phenotype-specific HF classification. This work provides the first comprehensive transcriptomic blueprint of circulating EVs in AHF, supporting their integration into multi-analyte liquid biopsy platforms for improved diagnostic precision and individualized disease management.

### 8.2. Practical Integration Strategies for c-miRNA Biomarker Panels in Heart Failure Management

Despite promising findings, several hurdles must be addressed before c-miRNAs can be fully translated into clinical use. The lack of harmonization in miRNA isolation and quantification methods remains a critical barrier to implementation. To improve reproducibility across independent studies, the development and widespread adoption of SOPs and best-practice guidelines are essential. Additionally, efforts should focus on the automation, miniaturization, and standardization of miRNA-based assays to facilitate their transition from bench to bedside. Thorough assessments of cost-effectiveness are also necessary to support implementation in clinical laboratory settings. To address the scarcity of large, well-characterized patient cohorts, the creation and expansion of accessible biobanks should be prioritized. These resources would enable multicenter validation and improve the statistical power of future studies, accelerating the clinical adoption of miRNA biomarkers. Future efforts should prioritize the development of harmonized detection protocols, the establishment of robust reference ranges, and the design of prospective, large-scale studies evaluating c-miRNA panels across HF subtypes and disease stages.

As these limitations are progressively addressed, attention must also turn to the practical steps required for clinical integration. To translate the diagnostic potential of c-miRNAs into routine practice, a structured and context-sensitive implementation strategy will be essential. In acute care settings, c-miRNA panels offer promising integration into rapid diagnostic workflows for HFpEF [309]. Given their stability in biofluids and compatibility with qPCR-based detection, c-miRNAs can be incorporated alongside NPs and troponin testing through automated order sets triggered by clinical presentation patterns. Assay optimization for 2–4-h turnaround times would align with emergency department decision-making timelines, enhancing early diagnostic precision. For CHF management, c-miRNA testing could be embedded into routine 3–6-month follow-up cycles, enabling longitudinal biomarker trending that informs medication titration and identifies patients requiring intensified monitoring or advanced therapies. The validated multi-miRNA panel (let-7b-5p, let-7e-5p, miR-21-5p, and miR-140-3p) demonstrated robust diagnostic performance in both preclinical and clinical settings, with several combinations achieving AUCs >0.9 in animal models and >0.75 in human cohorts [310]. These findings support the translational potential of c-miRNA panels as both acute diagnostic tools and chronic management biomarkers in HFpEF care.

Successful implementation requires deep integration with electronic health record systems through clinical decision support systems (CDSS) that provide real-time interpretation algorithms and actionable recommendations based on c-miRNA results in clinical context. Laboratory workflow optimization must ensure compatibility with existing automation systems, batch processing schedules, and quality control procedures, while standardized reporting templates provide consistent result formats that include clinical interpretation and recommended follow-up actions. The integration strategy should employ a phased rollout approach, beginning with pilot implementation in specialized heart failure clinics, expanding to general cardiology practices with comprehensive training programs, and ultimately extending to primary care settings with robust decision support systems [137].

This comprehensive integration approach ensures that c-miRNA panels become natural extensions of existing care patterns rather than disruptive additions, maximizing adoption potential through streamlined decision-making, reduced diagnostic uncertainty, and enhanced care coordination. Cost-effectiveness is achieved through optimized resource utilization, more precise risk stratification leading to appropriate care intensity, and improved patient outcomes resulting in reduced hospitalizations [309]. Alignment with evolving clinical guidelines from professional societies, regulatory compliance pathways, and integration with existing heart failure quality measures ensures that implementation strategies support rather than complicate existing healthcare delivery frameworks, ultimately maintaining workflow efficiency while enhancing clinical impact and patient safety standards.

### 8.3. Harnessing Artificial Intelligence to Drive Multi-Marker Integration in Heart Failure

The integration of artificial intelligence (AI), particularly machine learning (ML) algorithms, offers a transformative approach to maximizing the diagnostic and prognostic utility of multi-analyte liquid biopsy data in heart failure (HF). AI-driven tools can significantly enhance clinical decision-making by addressing key challenges in HF care, such as identifying patients at highest risk, predicting adverse outcomes, allocating therapy to those most likely to benefit, and enabling early detection of subclinical disease or impending decompensation [311]. Given the high dimensionality and complexity of circulating biomarkers, including c-miRNAs, cfDNA, and soluble proteins, ML models such as random forests, support vector machines, and neural networks are well suited to uncover intricate, nonlinear relationships that often elude traditional statistical methods.

Incorporating AI into clinical workflows has the potential to enable real-time, individualized risk stratification based on integrated biomarker profiles. Such models could augment existing risk scores and improve phenotype-specific management strategies. For instance, recent studies have shown that ML algorithms trained on multi-omic datasets including biomarkers, gene expression, and epigenetic features can accurately predict hospitalization and mortality risk across diverse HF phenotypes, including HFpEF and HFmrEF [312].

Future research should focus on developing and validating AI-assisted clinical decision-support systems using large, ethnically diverse, and well-phenotyped cohorts. These platforms will be essential to translating complex biomarker signatures into actionable insights and advancing precision cardiology in routine HF care.

### 8.4. Final Perspectives

Despite extensive research on miRNAs, cfDNA, and NPs as individual biomarkers in HF, comprehensive studies evaluating their combined diagnostic and prognostic utility remain inconclusive. This represents a critical knowledge gap, as multi-analyte approaches are theoretically superior to single-marker assays due to their capacity to capture multiple layers of disease biology. Combining the established clinical utility of traditional protein biomarkers with the mechanistic insights provided by c-miRNAs and the genomic information captured by cfDNA, integrated analysis of these molecular layers aligns with precision medicine goals, enabling earlier diagnosis, refined phenotypic stratification, and real-time therapeutic monitoring tailored to individual patient profiles [307,313]. 

Additionally, the integration of c-miRNAs into multi-analyte platforms, potentially alongside cfDNA, lncRNAs, and EV-derived RNAs, offers exciting opportunities for precision cardiology. These comprehensive approaches promise to enhance our understanding of disease mechanisms while providing more accurate diagnostic and prognostic tools. Bridging current knowledge gaps through harmonized workflows, robust validation, and integrative bioinformatics will be essential to realizing the full potential of liquid biopsy-guided precision medicine in HF.

## 9. Conclusions 

In conclusion, miRNAs represent a powerful and versatile platform for both biomarker discovery and targeted therapy in HF. Realizing their full potential will require concerted efforts to standardize methodologies, validate findings in large, diverse populations, and advance therapeutic development through robust clinical trials. Ultimately, circulating miRNAs hold the potential to reshape HF management by enabling earlier diagnosis, individualized therapy, and improved patient outcomes. The path forward demands collaborative efforts across multiple disciplines to overcome current limitations and fully harness the transformative potential of miRNA-based precision medicine in cardiovascular care. By integrating miRNA profiling into multi-analyte liquid biopsy frameworks, clinicians may achieve a more comprehensive molecular understanding of HF, enabling precision interventions tailored to patient-specific pathophysiology. Continued investment in translational research, coupled with advances in bioinformatics and molecular diagnostics, will be key to accelerating the clinical adoption of miRNA-guided strategies in routine cardiovascular practice.

## Figures and Tables

**Table 1:** Soluble Biomarkers for Heart Failure Management ^1^.

Biomarker	Pathophysiological Role	Clinical Utility	Guideline Recommendation
B-type natriuretic peptide (BNP), N-terminal proBNP (NT-proBNP)	Myocardial stretch, volume overload	Diagnosis, prognosis, therapy guidance	ESC ^2^ and AHA/ACC/HFSA ^3^ Class I–Strongly recommended for diagnosis & risk stratification
High-sensitivity troponin (hs-TnT/I)	Myocardial injury	Prognosis, especially in AHF ^4^	ESC: recommend for acute injury AHA/ACC/HFSA Class IIa–useful for risk stratification
Soluble suppression of tumorigenicity 2 (sST2)	Myocardial stress, inflammation, fibrosis	Prognosis (mortality, hospitalization)	ESC and AHA/ACC/HFSA Class IIb–may be considered for additive risk stratification
Galectin-3 (Gal-3)	Fibrosis, inflammation	Prognosis (especially CHF ^5^, HFpEF ^6^)	ESC and AHA/ACC/HFSA Class IIb, may consider in specific HF ^7^ cases
High sensitivity C-reactive protein (hs-CRP)	Systemic inflammation	Prognostic in HFpEF and comorbid conditions	Not routinely recommended by ESC or AHA/ACC/HFSA
Growth differentiation factor-15 (GDF-15)	Oxidative stress, inflammation	Emerging prognostic marker	Research/experimental only
Uric Acid	Oxidative metabolism, renal dysfunction	Associated with adverse outcomes	No formal guideline recommendation
Mid-regional pro-adrenomedullin (MR-proADM)	Hemodynamic stress, endothelial dysfunction	Prognostic; under investigation	Mentioned in ESC acute HF research context
Mid-regional pro-atrial natriuretic peptide (MR-proANP)	Atrial stretch, volume overload	Diagnosis and prognosis; comparable to BNP in acute HF	ESC Class I–alternative to BNP/NT-proBNP for AHF rule-out
Copeptin	Surrogate for vasopressin activity	Prognosis, especially in acute HF	Emerging; cited in ESC discussion, not endorsed in guidelines
Neutrophil gelatinase-associated lipocalin (NGAL)	Renal tubular injury	Predicts acute kidney injury in HF	Experimental, not guideline-recommended
Osteoprotegerin	Vascular calcification, inflammation	Emerging cardiovascular risk biomarker	Experimental use only

^1^ Data obtained from [3,34]; ^2^ ESC: 2023 European Society of Cardiology guidelines [3]; ^3^ AHA/ACC/HFSA: 2022 American Heart Association/American College of Cardiology/Heart Failure Society of America [34]; ^4^ AHF: Acute heart failure; ^5^ CHF: Chronic heart failure; ^6^ HFpEF: Heart failure with preserved ejection fraction; ^7^ HF: Heart failure.

**Table 3:** Representative miRNAs involved in cardiac hypertrophy and structural remodeling.

miRNA/Cluster	Functional Role	Ref.
miR-208a/b/499	Pro-hypertrophic; regulates myosin gene expression, represses negative regulators of muscle growth; involved in cardiac conduction	[165,166]
miR-195	Pro-hypertrophic; promotes pathological remodeling, apoptosis, and oxidative stress by targeting Bcl-2, HMGB1, and AMPK signaling	[167,168]
miR-106b~25 cluster (miR-106b/93/25 )	Regulates cardiomyocyte growth: high in neonates to support cell division, while in stressed adult hearts, overexpression restrains hypertrophy and loss worsens remodeling	[169,170]
miR-210	Hypoxia-induced protective miRNA; modulates apoptosis, mitochondrial metabolism, angiogenesis, and cell survival	[171]
miR-1	Regulates cardiac contractility and conduction; suppresses hypertrophy and apoptosis under stress	[140,172]
miR-133	Anti-hypertrophic; suppresses fibrosis and remodeling via RhoA, Cdc42, and Nelf-A/WHSC2; maintains myocardial structure	[140,173]

**Table 4:** Examples of miRNAs in myocardial fibrosis and ECM regulation.

miRNA	Functional Role	Ref.
miR-21	Pro-fibrotic; promotes fibroblast survival and ECM ^1^ synthesis by targeting PTEN, SPRY1, and TGFBR3	[178,179]
miR-29 (a/b/c)	Anti-fibrotic; represses ECM genes (collagens, fibrillins); regulates anti-apoptotic genes	[180]
miR-214	Pro-fibrotic; enhances collagen synthesis and fibroblast proliferation via ERK1/2 MAPK activation	[181]
miR-144-3p	Pro-fibrotic; suppresses PTEN, promotes fibroblast proliferation and ECM protein expression	[182]
miR-30/miR-30c	Anti-fibrotic; inhibits CTGF ^2^; oxidative modification of miR-30c can lead to aberrant ECM regulation	[183]
miR-133	Anti-fibrotic; regulates multiple fibrosis pathways (RhoA, MAPK, TGF-β/Smad, PI3K/Akt)	[173]
miR-590-3p	Anti-fibrotic and regenerative; reduces fibrosis and promotes cardiac regeneration via TSC22D2 and PKM2	[184]

^1^ ECM: Excessive extracellular matrix, ^2^ CTGF: Connective tissue growth factor.

**Table 5:** Representative miRNAs that regulate immune activation in heart failure.

miRNA	Source	Immune Targets/Effects	Ref.
miR-21	Cardiac fibroblasts	Spry1, PTEN, TGFBR3; promotes fibrosis and immune activation	[178,187,188]
miR-155	Macrophages, T cells	SOCS-1; enhances inflammation, leukocyte infiltration, and injury	[154,189,190]
miR-146a/b/-5p	Cardiomyocytes, exosomes	TRAF6; feedback inhibition of NF-κB, regulates immune activation	[191,192]
miR-223	Myeloid cells	Cytokine production, immune cell activation	[186]
miR-17~92 cluster	Immune cells	Immune cell proliferation and cytokine signaling	[186]
miR-10a/b	Cardiomyocyte exosomes	Induces IL-6 in macrophages under hypoxia	[193]
miR-143/423	Cardiomyocyte exosomes	Amplifies cytokine expression (IL-6), enhances inflammation	[194]
miR-939-5p	Circulating blood	Correlated with inflammatory cytokines. regulated by lncRNAs	[195]
miR-181b	CDCs ^1^	Promotes M2 macrophage polarization, cardio-protection	[196]
miR-26a	CDCs	Enhances phagocytosis, macrophage efferocytosis	[197]
miR-27a-5p	CDCs	Modulates macrophage phenotype post-I/R injury	[198]
miR-222	Endothelial cells	Reduces ICAM-1 ^2^ expression, anti-inflammatory	[199]
miR-126	Endothelial cells	Reduces VCAM-1 ^3^, ICAM-1; attenuates immune infiltration	[200]

^1^ CDC: Cardio sphere-derived cell; ^2^ ICAM-1/intercellular cell adhesion molecules. ^3^ VCAM-1: vascular cell adhesion molecules.

**Table 6:** Representative miRNAs regulating cardiomyocyte apoptosis and survival.

miRNA	Target Genes/ Pathways	Role in Apoptosis	Ref.
miR-103-3p	Hlf (hepatic leukemia factor)	Promotes	[209]
miR-34a	Bcl-2, SIRT1, Notch1, PNUTS	Promotes	[156,210]
miR-15 family	Bcl-2, Arl2, Cyclin D2, MFN2, SIRT3, c-Myb, BCL2L2	Promotes	[211,216]
miR-30a-5p	E2F3	Promotes	[212]
miR-26a	PTEN, PI3K/Akt, JAK/STAT	Inhibits	[217]
miR-24	Bim, p53	Inhibits	[214]
miR-214	PTEN, Bim1, PI3K/Akt	Inhibits	[215]

**Table 7:** Examples of miRNAs in Angiogenesis and Endothelial Function in Heart Failure.

miRNA	Category	Key Targets /Pathways	Functional Role	Ref.
miR-126	Pro-angiogenic	SPRED1, PIK3R2; enhances VEGF signaling	Promotes endothelial proliferation and neovascularization	[130,159]
miR-132	Pro-angiogenic	PTEN/PI3K/Akt	Enhances angiogenesis and reduces fibrosis	[221,227]
miR-210	Pro-angiogenic	Ephrin-A3, PTP1B	Induces VEGF/FGF secretion and promotes endothelial tube formation	[222]
miR-92a	Anti-angiogenic/Dysfunction- promoting	ITGA5, SIRT1	Inhibits angiogenesis, promotes EndMT, and impairs endothelial gene expression	[223,224]
miR-34a	Anti-angiogenic/Dysfunction-promoting	SIRT1, eNOS	Induces endothelial senescence and dysfunction; enhances cardiac fibrosis	[210]
miR-1285-3p	Anti-angiogenic/Dysfunction-promoting	STAT3/VEGFA	Suppresses endothelial proliferation and induces apoptosis	[225]
miR-200b	Anti-angiogenic/Dysfunction- promoting	VEGF-A, VEGFR1, VEGFR2, ETS1, GATA2	Reduces NO bioavailability, increases oxidative stress, and impairs angiogenesis	[226]

**Table 8:** Representative examples of Diagnostic Utility of Circulating miRNAs in Heart Failure.

miRNA(s)	Source	Association	Regulation	Ref.
miR-423-5p	Plasma	HF diagnosis, correlation with NT-proBNP ^1^	Upregulated	[228]
miR-499	Plasma	AHF ^2^ diagnosis, cardiac injury specificity	Upregulated	[229]
miR-122-5p	Plasma	AHF, hepatic congestion	Upregulated	[230]
miR-423-5p, miR-320a, miR-22, miR-92b	Serum	Chronic systolic-HF ^3^ diagnosis. 4-miRNA score that discriminates HF from controls and score correlated with NT-proBNP, QRS widening, and left ventricle/left atrium dilatation	Upregulated	[231]
miR-155	Plasma	Chronic HF, ventricular arrhythmias (AT1R 1166C)	Downregulated	[232]
	Coronary- sinus plasma	Trans-cardiac release, oxidative-stress HF	Upregulated	[233]
miR-30c, miR-146a, miR-221, miR-328, and miR-375	Plasma	HFrEF ^4^ vs. HFpEF ^5^ classification	Differentially expressed	[234]
miR-107, miR-139, miR-142-5p	PBMCs ^6^	Downregulated in both NIDCM ^7^ and ICM ^8^	Downregulated	[235]
miR-142-3p, miR-29b	PBMCs (NIDCM)	Upregulated in NIDCM ^9^	Upregulated	[235]
miR-125b, miR-497	PBMCs (ICM)	Downregulated in ICM	Downregulated	[235]

^1^ NT-proBNP: N-terminal prohormone of B-type natriuretic peptide; ^2^ AHF: Acute heart failure; ^3^ HF: Heart failure; ^4^ HFrEF: HF with reduced ejection fraction; ^5^ HFpEF: HF with preserved ejection fraction; ^6^ PBMCs: peripheral blood mononuclear cell; ^7^ NIDC: non-ischemic dilated cardiomyopathy; ^8^ ICM: ischemic cardiomyopathy; ^9^ NIDCM: non-ischemic dilated cardiomyopathy.

**Table 9:** Combinations of c-miRNAs and natriuretic peptides in heart failure.

Study (Design/Cohort)	miRNA(s) Tested (Direction)	NP Comparator	HF Context/Endpoint	Incremental Value over NP Alone	Ref.
Dahl salt-sensitive rats (HF ^1^ n = 13, controls n = 9) NYHA ^2^ II outpatient subset (n = 39)	miR-210 ↑ in rat plasma, mononuclear cells and skeletal muscle; lower baseline miR-210 in patients predicted BNP improvement	BNP ^3^	Pre-clinical chronic HF model; prognostic change in BNP (rats and patients)	Rats: c-miR-210 strongly correlated with BNP Patients: low miR-210 identified those with falling BNP at 3 months follow-up despite no baseline cross-sectional correlation	[281]
Validation cohort; 150 patients with dyspnea, including 44 with HF	17 assayed; miR-103, -142-3p, -30b, -342-3p ↓ in HF	NT-proBNP ^4^ (AUC ^5^ 0.896)	Rule-in HF vs. non-cardiac dyspnea	miR-423-5p + NT-proBNP ↑AUC by 3.2% (*p* = 0.030); all miRNAs + NT-proBNP ↑AUC by 4.6% (*p* = 0.013)	[282]
Case-control; (n = 225)	miR-panel: miR-30c, -146a, -221, -328, -375	BNP	HFpEF ^6^ vs. HFrEF ^7^ discrimination	Panel + BNP AUC > 0.82 for subtype classification (vs. BNP alone < 0.75)	[234]
Two cohorts; (total n = 176)	12 DE miRNAs; key: miR-125a-5p, -550a-5p, -638 ↑ in HFrEF; miR-190a ↓	NT-proBNP	HFrEF vs. HFpEF diagnosis	Multi-miRNA panel AUC 0.80; panel and NT-proBNP AUC 1.00 for phenotype discrimination	[283]
Systematic review and meta-analysis (10 studies)	Mixed panels (33 tests) and miR-423-5p only	BNP	HF diagnosis (all phenotypes)	Mixed miRNA and BNP: Sensitivity 0.85, Specificity 0.81, AUC 0.915 (vs. BNP alone AUC 0.929; miRNA panels alone AUC 0.799)	[284]
Discovery and 2 validations; 546 + 235 patients	Panel of eight miRs	NT-proBNP	Non-AHF ^8^ detection; HFpEF/HFrEF discrimination	Panel AUC 0.96; panel and NT-proBNP AUC 0.99; reclassified 72–88% of NT-proBNP false negatives (mostly HFpEF)	[285]

^1^ HF: Heart failure; ^2^ NYHA: New York Heart Association; ^3^ BNP: B-type natriuretic peptide; ^4^ NT-proBNP: N-terminal pro-BNP; ^5^ AUC: Area under curve; ^6^ HFpEF: Heart failure with preserved ejection fraction; ^7^ HFrEF: Heart failure with reduced ejection fraction; ^8^ AHF: Acute heart failure; ↑: Increase; ↓: Decrease.

**Table 10:** Examples of studies evaluating the incremental value of c-miRNAs when combined with established biomarkers for enhancing heart failure diagnosis, subtyping, or risk stratification.

Study (Design/Cohort)	miRNA(s) and Protein(s) Tested	HF Setting/ Endpoint	Incremental Value over Proteins Alone	Ref.
Prospective; 139 symptomatic HF ^1^	32-miR panel (PC3: miR-21-5p/-30d-5p/-92a-3p) (PC4: miR-1-3p/-133a-3p) NT-proBNP ^2^, hs-TnI ^3^, suPAR ^4^, Gal-3 ^5^	Echo structure and composite endpoint	Adding PC3 + PC4 raised Harrell C from 0.75 → 0.82 and remained significant after Gal-3 adjustment	[276]
Cross-sectional. 45 controls and 135 T2DM ^6^ (45 DM ^7^, 45 DM + SCAD ^8^, 45 DM +AHF ^9^)	miR-1 ↓, miR-21 ↑ paired with NT-proBNP and Gal-3	Detection of acute HF in otherwise asymptomatic T2DM; correlation with fibrosis and load markers	miR-1 inversely (r = −0.89) and miR-21 positively (r = 0.73–0.76) correlated with NT-proBNP and Gal-3; combined miR-21 + Gal-3 + NT- proBNP markedly improved ROC ^10^ curves vs. any single marker	[286]
Hypertensive patients with LVH ^11^ and symptomatic HF	miR-1, miR-21 + Gal-3	Fibrosis burden in hypertensive HF	Both miRNAs down-regulated; levels tracked Gal-3 and LV wall thickness (*p* < 0.01)	[287]
50 CHF patients and 30 controls	miR-214 ↑ + Gal-3	Baseline vs. 6-month therapy response	miR-214: AUC ^12^ 0.916; Gal-3: AUC 0.852; pre-treatment values predicted response (AUC ≈ 0.88–0.90)	[288]
100 patients (AMI/angina/controls)	miR-145 ↓, miR-21 ↑ hs-TnI, CK-MB ^13^	Infarct size/injury severity	miR-145 strongly inversely correlated with peak hs-TnI (ρ = −0.62, *p* < 0.0001); miR-21 positively correlated with injury markers	[289]
GISSI-HF; 953 chronic HF	miR-132 NT-proBNP, hs-TnT ^14^, hs-CRP ^15^, PTX-3 ^16^	46-month HF rehospitalization and mortality	Low miR-132 predicted HF admission (HR 0.79); adding miR-132 to clinical and NP model improved cNRI by 0.21	[221]
Cross-sectional; 270 hypertensive HF	miR-210-3p ↑ + NT-proBNP, sST2 ^17^, Gal-3	HFpEF ^18^ vs. HFrEF ^19^ differentiation	miR-210-3p AUC 0.79 (87% Sensitivity/54% Specificity); correlated with sST2, Gal-3, NP & MAGGIC score (r ≥ 0.52)	[290]

^1^ HF: Heart failure; ^2^ NT-proBNP: N-terminal pro-B-type natriuretic peptide; ^3^ hs-TnI: High-sensitivity cardiac troponin I; ^4^ suPAR: Soluble urokinase-type plasminogen-activator receptor; ^5^ Gal-3: Galectin-3; ^6^ T2DM: Type 2 diabetes mellitus; ^7^ DM: Diabetic mellitus group; ^8^ SCAD: Silent coronary artery disease; ^9^ AHF: Acute heart failure; ^10^ ROC: Receiver-operating-characteristic curve; ^11^ LVH: Left-ventricular hypertrophy; ^12^ AUC: Area under the receiver-operating-characteristic curve; ^13^ CK-MB: Creatine-kinase myocardial band; ^14^ hs-TnT: High-sensitivity cardiac troponin T; ^15^ hs-CRP: High-sensitivity C-reactive protein; ^16^ PTX-3: Pentraxin 3; ^17^ sST2: Soluble suppression of tumorigenicity 2; ^18^ HFpEF: Heart failure with preserved ejection fraction; ^19^ HFrEF: Heart failure with reduced ejection fraction ↑: Increase; ↓: Decrease.

## Data Availability

No new data was created in this work.

## References

[1] Banerjee A., Mendis S. (2013). Heart failure: The need for global health perspective. Curr. Cardiol. Rev..

[2] Groenewegen A., Rutten F.H., Mosterd A., Hoes A.W. (2020). Epidemiology of heart failure. Eur. J. Heart. Fail..

[3] McDonagh T.A., Metra M., Adamo M., Gardner R.S., Baumbach A., Böhm M., Burri H., Butler J., Čelutkienė J., Chioncel O. (2024). 2023 Focused Update of the 2021 ESC Guidelines for the diagnosis and treatment of acute and chronic heart failure: Developed by the task force for the diagnosis and treatment of acute and chronic heart failure of the European Society of Cardiology (ESC) With the special contribution of the Heart Failure Association (HFA) of the ESC. Eur. J. Heart. Fail..

[4] Sapna F., Raveena F., Chandio M., Bai K., Sayyar M., Varrassi G., Khatri M., Kumar S., Mohamad T. (2023). Advancements in heart failure management: A comprehensive narrative review of emerging therapies. Cureus.

[5] Bozkurt B., Coats A.J.S., Tsutsui H., Abdelhamid C.M., Adamopoulos S., Albert N., Anker S.D., Atherton J., Böhm M., Butler J. (2021). Universal definition and classification of heart failure: A report of the Heart Failure Society of America, Heart Failure Association of the European Society of Cardiology, Japanese Heart Failure Society and Writing Committee of the Universal Definition of Heart Failure: Endorsed by the Canadian Heart Failure Society, Heart Failure Association of India, Cardiac Society of Australia and New Zealand, and Chinese Heart Failure Association. Eur. J. Heart Fail..

[6] Savarese G., Stolfo D., Sinagra G., Lund L.H. (2022). Heart failure with mid-range or mildly reduced ejection fraction. Nat. Rev. Cardiol..

[7] Savarese G., Vedin O., D’Amario D., Uijl A., Dahlström U., Rosano G., Lam C.S.P., Lund L.H. (2019). Prevalence and prognostic implications of longitudinal ejection fraction change in heart failure. JACC Heart Fail..

[8] Vedin O., Lam C.S.P., Koh A.S., Benson L., Teng T.H.K., Tay W.T., Braun O., Savarese G., Dahlström U., Lund L.H. (2017). Significance of ischemic heart disease in patients with heartf failure and preserved, midrange, and reduced ejection fraction: A nationwide cohort study. Circ. Heart Fail..

[9] Fonarow G.C., Stough W.G., Abraham W.T., Albert N.M., Gheorghiade M., Greenberg B.H., O’Connor C.M., Sun J.L., Yancy C.W., Young J.B. (2007). Characteristics, treatments, and outcomes of patients with preserved systolic function hospitalized for heart failure: A report from the OPTIMIZE-HF Registry. J. Am. Coll. Cardiol..

[10] Chioncel O., Lainscak M., Seferovic P.M., Anker S.D., Crespo-Leiro M.G., Harjola V.P., Parissis J., Laroche C., Piepoli M.F., Fonseca C. (2017). Epidemiology and one-year outcomes in patients with chronic heart failure and preserved, mid-range and reduced ejection fraction: An analysis of the ESC Heart Failure Long-Term Registry. Eur. J. Heart Fail..

[11] van Heerebeek L., Borbély A., Niessen H.W.M., Bronzwaer J.G.F., van der Velden J., Stienen G.J.M., Linke W.A., Laarman G.J., Paulus W.J. (2006). Myocardial structure and function differ in systolic and diastolic heart failure. Circulation.

[12] Czepluch F.S., Wollnik B., Hasenfuß G. (2018). Genetic determinants of heart failure: Facts and numbers. ESC Heart Fail..

[13] Roth G.A., Mensah G.A., Johnson C.O., Addolorato G., Ammirati E., Baddour L.M., Barengo N.C., Beaton A.Z., Benjamin E.J., Benziger C.P. (2020). Global burden of cardiovascular diseases and risk factors, 1990-2019: Update from the GBD 2019 study. J. Am. Coll. Cardiol..

[14] Bozkurt B., Colvin M., Cook J., Cooper L.T., Deswal A., Fonarow G.C., Francis G.S., Lenihan D., Lewis E.F., McNamara D.M. (2016). Current diagnostic and treatment strategies for specific dilated cardiomyopathies: A scientific statement from the American Heart Association. Circulation.

[15] Cabac-Pogorevici I., Muk B., Rustamova Y., Kalogeropoulos A., Tzeis S., Vardas P. (2020). Ischaemic cardiomyopathy. Pathophysiological insights, diagnostic management and the roles of revascularisation and device treatment. Gaps and dilemmas in the era of advanced technology. Eur. J. Heart Fail..

[16] Wu A.H. (2007). Management of patients with non-ischaemic cardiomyopathy. Heart.

[17] McKenna W.J., Maron B.J., Thiene G. (2017). Classification, epidemiology, and global burden of cardiomyopathies. Circ. Res..

[18] Elendu C., Amaechi D.C., Elendu T.C., Fiemotonghan B.E., Okoye O.K., Agu-Ben C.M., Onyekweli S.O., Amapu D.A., Ikpegbu R., Asekhauno M. (2024). A comprehensive review of heart failure: Unraveling the etiology, decoding pathophysiological mechanisms, navigating diagnostic modalities, exploring pharmacological interventions, advocating lifestyle modifications, and charting the horizon of emerging therapies in the complex landscape of chronic cardiac dysfunction. Medicine.

[19] Estep J.D., Salah H.M., Kapadia S.R., Burkhoff D., Lala A., Butler J., Hall S., Fudim M. (2024). HFSA scientific statement: Update on device based therapies in heart failure. J. Card. Fail..

[20] Shahim B., Kapelios C.J., Savarese G., Lund L.H. (2023). Global public health burden of heart failure: An updated review. Card. Fail. Rev..

[21] Savarese G., Becher P.M., Lund L.H., Seferovic P., Rosano G.M.C., Coats A.J.S. (2023). Global burden of heart failure: A comprehensive and updated review of epidemiology. Cardiovasc. Res..

[22] Smiseth O.A., Donal E., Boe E., Ha J.W., Fernandes J.F., Lamata P. (2023). Phenotyping heart failure by echocardiography: Imaging of ventricular function and haemodynamics at rest and exercise. Eur. Heart J. Cardiovasc. Imaging.

[23] Gallagher J., Watson C., Campbell P., Ledwidge M., McDonald K. (2017). Natriuretic peptide-based screening and prevention of heart failure. Card. Fail. Rev..

[24] Remmelzwaal S., van Ballegooijen A.J., Schoonmade L.J., Dal Canto E., Handoko M.L., Henkens M., van Empel V., Heymans S.R.B., Beulens J.W.J. (2020). Natriuretic peptides for the detection of diastolic dysfunction and heart failure with preserved ejection fraction-a systematic review and meta-analysis. BMC Med..

[25] Paul T., Nadeem A.U.R., Naqvi S.M., Liu J., Ali K.A. (2025). Heart failure with preserved ejection fraction and low B-type natriuretic peptide: A diagnostic dilemma. Cureus.

[26] Madamanchi C., Alhosaini H., Sumida A., Runge M.S. (2014). Obesity and natriuretic peptides, BNP and NT-proBNP: Mechanisms and diagnostic implications for heart failure. Int. J. Cardiol..

[27] Schindler E.I., Szymanski J.J., Hock K.G., Geltman E.M., Scott M.G. (2016). Short- and long-term biologic variability of galectin-3 and other cardiac biomarkers in patients with stable heart failure and healthy adults. Clin. Chem..

[28] Jacob R., Khan M. (2018). Cardiac biomarkers: What is and what can be. Indian J. Cardiovasc. Dis. Women WINCARS.

[29] Çakmak H.A., Demir M. (2020). MicroRNA and cardiovascular diseases. Balkan Med. J..

[30] Zhou S.-s., Jin J.-p., Wang J.-q., Zhang Z.-g., Freedman J.H., Zheng Y., Cai L. (2018). miRNAS in cardiovascular diseases: Potential biomarkers, therapeutic targets and challenges. Acta Pharmacol. Sin..

[31] Huang S., Zhou Y., Zhang Y., Liu N., Liu J., Liu L., Fan C. (2025). Advances in microRNA therapy for heart failure: Clinical trials, preclinical studies, and controversies. Cardiovasc. Drugs Ther..

[32] O’Brien J., Hayder H., Zayed Y., Peng C. (2018). Overview of microRNA biogenesis, mechanisms of actions, and circulation. Front. Endocrinol..

[33] Pang J.K.S., Phua Q.H., Soh B.-S. (2019). Applications of miRNAs in cardiac development, disease progression and regeneration. Stem. Cell Res. Ther..

[34] Heidenreich P.A., Bozkurt B., Aguilar D., Allen L.A., Byun J.J., Colvin M.M., Deswal A., Drazner M.H., Dunlay S.M., Evers L.R. (2022). 2022 AHA/ACC/HFSA Guideline for the Management of Heart Failure: A Report of the American College of Cardiology/American Heart Association Joint Committee on Clinical Practice Guidelines. Circulation.

[35] Topf A., Mirna M., Ohnewein B., Jirak P., Kopp K., Fejzic D., Haslinger M., Motloch L.J., Hoppe U.C., Berezin A. (2020). The diagnostic and therapeutic value of multimarker analysis in heartfailure. An approach to biomarker-targeted therapy. Front. Cardiovasc. Med..

[36] Baethge C., Goldbeck-Wood S., Mertens S. (2019). SANRA—A scale for the quality assessment of narrative review articles. Res. Integr. Peer Rev..

[37] Alix-Panabières C., Marchetti D., Lang J.E. (2023). Liquid biopsy: From concept to clinical application. Sci. Rep..

[38] Ma L., Guo H., Zhao Y., Liu Z., Wang C., Bu J., Sun T., Wei J. (2024). Liquid biopsy in cancer: Current status, challenges and future prospects. Sig. Transduct. Target Ther..

[39] Anitha K., Posinasetty B., Naveen Kumari K., Chenchula S., Padmavathi R., Prakash S., Radhika C. (2024). Liquid biopsy for precision diagnostics and therapeutics. Clin. Chim. Acta.

[40] Davidson S.M., Andreadou I., Antoniades C., Bartunek J., Basso C., Brundel B., Byrne R.A., Chiva-Blanch G., da Costa Martins P., Evans P.C. (2025). Opportunities and challenges for the use of human samples in translational cardiovascular research: A scientific statement of the ESC Working Group on Cellular Biology of the Heart, the ESC Working Group on Cardiovascular Surgery, the ESC Council on Basic Cardiovascular Science, the ESC Scientists of Tomorrow, the European Association of Percutaneous Cardiovascular Interventions of the ESC, and the Heart Failure Association of the ESC. Cardiovasc. Res..

[41] Abudurexiti M., Aimaier S., Wupuer N., Duan D., Abudouwayiti A., Nuermaimaiti M., Mahemuti A. (2025). Identification of noval diagnostic biomarker for HFpEF based on proteomics and machine learning. Proteome Sci..

[42] Mueller T., Leitner I., Egger M., Haltmayer M., Dieplinger B. (2015). Association of the biomarkers soluble ST2, galectin-3 and growth-differentiation factor-15 with heart failure and other non-cardiac diseases. Clin. Chim. Acta.

[43] Chauin A. (2021). The main causes and mechanisms of increase in cardiac troponin concentrations other than acute myocardial infarction (Part 1): Physical exertion, inflammatory heart disease, pulmonary embolism, renal failure, sepsis. Vasc. Health Risk Manag..

[44] Berezin A.E., Berezin A.A. (2023). Biomarkers in heart failure: From research to clinical practice. Ann. Lab. Med..

[45] Kambayashi Y., Nakao K., Mukoyama M., Saito Y., Ogawa Y., Shiono S., Inouye K., Yoshida N., Imura H. (1990). Isolation and sequence determination of human brain natriuretic peptide in human atrium. FEBS Lett..

[46] Samad M., Malempati S., Restini C.B.A. (2023). Natriuretic peptides as biomarkers: Narrative review and considerations in cardiovascular and respiratory dysfunctions. Yale J. Biol. Med..

[47] Emdin M., Passino C., Prontera C., Fontana M., Poletti R., Gabutti A., Mammini C., Giannoni A., Zyw L., Zucchelli G. (2007). Comparison of brain natriuretic peptide (BNP) and amino-terminal ProBNP for early diagnosis of heart failure. Clin. Chem..

[48] Gallo G., Rubattu S., Autore C., Volpe M. (2023). Natriuretic peptides: It is time for guided therapeutic strategies based on their molecular mechanisms. Int. J. Mol. Sci..

[49] Volpe M., Gallo G., Rubattu S. (2023). Endocrine functions of the heart: From bench to bedside. Eur. Heart J..

[50] Huelsmann M., Neuhold S., Strunk G., Moertl D., Berger R., Prager R., Abrahamian H., Riedl M., Pacher R., Luger A. (2008). NT-proBNP has a high negative predictive value to rule-out short-term cardiovascular events in patients with diabetes mellitus. Eur. Heart J..

[51] Maisel A.S., Krishnaswamy P., Nowak R.M., McCord J., Hollander J.E., Duc P., Omland T., Storrow A.B., Abraham W.T., Wu A.H. (2002). Rapid measurement of B-type natriuretic peptide in the emergency diagnosis of heart failure. N. Engl. J. Med..

[52] Wallén T., Landahl S., Hedner T., Hall C., Saito Y., Nakao K. (1997). Atrial natriuretic peptides predict mortality in the elderly. J. Intern. Med..

[53] Roberts E., Ludman A.J., Dworzynski K., Al-Mohammad A., Cowie M.R., McMurray J.J., Mant J. (2015). The diagnostic accuracy of the natriuretic peptides in heart failure: Systematic review and diagnostic meta-analysis in the acute care setting. BMJ.

[54] Mehra M.R., Uber P.A., Park M.H., Scott R.L., Ventura H.O., Harris B.C., Frohlich E.D. (2004). Obesity and suppressed B-type natriuretic peptide levels in heart failure. J. Am. Coll. Cardiol..

[55] Bayes-Genis A., Lloyd-Jones D.M., van Kimmenade R.R., Lainchbury J.G., Richards A.M., Ordoñez-Llanos J., Santaló M., Pinto Y.M., Januzzi J.L. (2007). Effect of body mass index on diagnostic and prognostic usefulness of amino-terminal pro-brain natriuretic peptide in patients with acute dyspnea. Arch. Intern. Med..

[56] Redfield M.M., Rodeheffer R.J., Jacobsen S.J., Mahoney D.W., Bailey K.R., Burnett J.C. (2002). Plasma brain natriuretic peptide concentration: Impact of age and gender. J. Am. Coll. Cardiol..

[57] Wang T.J., Larson M.G., Levy D., Leip E.P., Benjamin E.J., Wilson P.W., Sutherland P., Omland T., Vasan R.S. (2002). Impact of age and sex on plasma natriuretic peptide levels in healthy adults. Am. J. Cardiol..

[58] Gupta D.K., Daniels L.B., Cheng S., deFilippi C.R., Criqui M.H., Maisel A.S., Lima J.A., Bahrami H., Greenland P., Cushman M. (2017). Differences in natriuretic peptide levels by race/ethnicity (from the multi-ethnic study of atherosclerosis). Am. J. Cardiol..

[59] Kuwahara K. (2021). The natriuretic peptide system in heart failure: Diagnostic and therapeutic implications. Pharmacol. Ther..

[60] Sun R.R., Lu L., Liu M., Cao Y., Li X.C., Liu H., Wang J., Zhang P.Y. (2014). Biomarkers and heart disease. Eur. Rev. Med. Pharmacol. Sci..

[61] Schmitter D., Cotter G., Voors A.A. (2014). Clinical use of novel biomarkers in heart failure: Towards personalized medicine. Heart Fail. Rev..

[62] Pouleur A.C. (2015). Which biomarkers do clinicians need for diagnosis and management of heart failure with reduced ejection fraction?. Clin. Chim. Acta.

[63] Newlaczyl A.U., Yu L.G. (2011). Galectin-3--a jack-of-all-trades in cancer. Cancer Lett..

[64] Frunza O., Russo I., Saxena A., Shinde A.V., Humeres C., Hanif W., Rai V., Su Y., Frangogiannis N.G. (2016). Myocardial galectin-3 expression is associated with remodeling of the pressure-overloaded heart and may delay the hypertrophic response without affecting survival, dysfunction, and cardiac fibrosis. Am. J. Pathol..

[65] Ho J.E., Liu C., Lyass A., Courchesne P., Pencina M.J., Vasan R.S., Larson M.G., Levy D. (2012). Galectin-3, a marker of cardiac fibrosis, predicts incident heart failure in the community. J. Am. Coll. Cardiol..

[66] van der Velde A.R., Gullestad L., Ueland T., Aukrust P., Guo Y., Adourian A., Muntendam P., van Veldhuisen D.J., de Boer R.A. (2013). Prognostic value of changes in galectin-3 levels over time in patients with heart failure: Data from CORONA and COACH. Circ. Heart Fail..

[67] Siew J.J., Chen H.M., Chen H.Y., Chen H.L., Chen C.M., Soong B.W., Wu Y.R., Chang C.P., Chan Y.C., Lin C.H. (2019). Galectin-3 is required for the microglia-mediated brain inflammation in a model of Huntington’s disease. Nat. Commun..

[68] Zaborska B., Sikora-Frąc M., Smarż K., Pilichowska-Paszkiet E., Budaj A., Sitkiewicz D., Sygitowicz G. (2023). The role of galectin-3 in heart failure-the diagnostic, prognostic and therapeutic potential-where do we stand?. Int. J. Mol. Sci..

[69] Suthahar N., Meijers W.C., Silljé H.H.W., Ho J.E., Liu F.T., de Boer R.A. (2018). Galectin-3 activation and inhibition in heart failure and cardiovascular disease: An update. Theranostics.

[70] Srivatsan V., George M., Shanmugam E. (2020). Utility of galectin-3 as a prognostic biomarker in heart failure: Where do we stand?. Eur. J. Prev. Cardiol..

[71] Mueller T., Dieplinger B. (2016). Soluble ST2 and galectin-3: What we know and don’t know analytically. EJIFCC.

[72] Dieplinger B., Mueller T. (2015). Soluble ST2 in heart failure. Clin. Chim. Acta.

[73] Mueller T., Jaffe A.S. (2015). Soluble ST2-analytical considerations. Am. J. Cardiol..

[74] Villarreal D.O., Weiner D.B. (2014). Interleukin 33: A switch-hitting cytokine. Curr. Opin. Immunol..

[75] Pusceddu I., Dieplinger B., Mueller T. (2019). ST2 and the ST2/IL-33 signalling pathway-biochemistry and pathophysiology in animal models and humans. Clin. Chim. Acta.

[76] Veeraveedu P.T., Sanada S., Okuda K., Fu H.Y., Matsuzaki T., Araki R., Yamato M., Yasuda K., Sakata Y., Yoshimoto T. (2017). Ablation of IL-33 gene exacerbate myocardial remodeling in mice with heart failure induced by mechanical stress. Biochem. Pharmacol..

[77] Wojtczak-Soska K., Pietrucha T., Sakowicz A., Lelonek M. (2013). Soluble ST2 protein in chronic heart failure is independent of traditional factors. Arch. Med. Sci..

[78] Lotierzo M., Dupuy A.M., Kalmanovich E., Roubille F., Cristol J.P. (2020). sST2 as a value-added biomarker in heart failure. Clin. Chim. Acta.

[79] Zhang T., Xu C., Zhao R., Cao Z. (2021). Diagnostic value of sST2 in cardiovascular diseases: A systematic review and meta-analysis. Front. Cardiovasc. Med..

[80] Thygesen K., Alpert J.S., Jaffe A.S., Simoons M.L., Chaitman B.R., White H.D., Thygesen K., Alpert J.S., White H.D., Jaffe A.S. (2012). Third universal definition of myocardial infarction. J. Am. Coll. Cardiol..

[81] Januzzi J.L., Filippatos G., Nieminen M., Gheorghiade M. (2012). Troponin elevation in patients with heart failure: On behalf of the third Universal Definition of Myocardial Infarction Global Task Force: Heart Failure Section. Eur. Heart J..

[82] Jacob J., Roset A., Miró Ò., Alquézar A., Herrero P., Martín-Sanchez F.J., Möckel M., Müller C., Llorens P. (2017). EAHFE—TROPICA2 study. Prognostic value of troponin in patients with acute heart failure treated in Spanish hospital emergency departments. Biomarkers.

[83] Perna E.R., Macin S.M., Canella J.P., Augier N., Stival J.L., Cialzeta J.R., Pitzus A.E., Garcia E.H., Obregón R., Brizuela M. (2004). Ongoing myocardial injury in stable severe heart failure: Value of cardiac troponin T monitoring for high-risk patient identification. Circulation.

[84] Chen W.C., Tran K.D., Maisel A.S. (2010). Biomarkers in heart failure. Heart.

[85] Sze J., Mooney J., Barzi F., Hillis G.S., Chow C.K. (2016). Cardiac troponin and its relationship to cardiovascular outcomes in community populations—A systematic review and meta-analysis. Heart Lung Circ..

[86] Wettersten N., Maisel A. (2015). Role of cardiac troponin levels in acute heart failure. Card. Fail. Rev..

[87] Braghieri L., Badwan O.Z., Skoza W., Fares M., Menon V. (2023). Evaluating troponin elevation in patients with chronic kidney disease and suspected acute coronary syndrome. Clevel. Clin. J. Med..

[88] Long B., Belcher C.N., Koyfman A., Bronner J.M. (2020). Interpreting troponin in renal disease: A narrative review for emergency clinicians. Am. J. Emerg. Med..

[89] Gupta S., Alagona P. (2008). Troponins: Not always a myocardial infarction. Am. J. Med..

[90] Kociol R.D., Pang P.S., Gheorghiade M., Fonarow G.C., O’Connor C.M., Felker G.M. (2010). Troponin elevation in heart failure: Prevalence, mechanisms, and clinical implications. J. Am. Coll. Cardiol..

[91] Pös O., Biró O., Szemes T., Nagy B. (2018). Circulating cell-free nucleic acids: Characteristics and applications. Eur. J. Hum. Genet..

[92] Fleischhacker M., Schwab M. (2009). Circulating Nucleic Acids. Encyclopedia of Cancer.

[93] Biró O., Hajas O., Nagy-Baló E., Soltész B., Csanádi Z., Nagy B. (2018). Relationship between cardiovascular diseases and circulating cell-free nucleic acids in human plasma. Biomark. Med..

[94] Benincasa G., Mansueto G., Napoli C. (2019). Fluid-based assays and precision medicine of cardiovascular diseases: The ‘hope’ for Pandora’s box?. J. Clin. Pathol..

[95] Schiano C., Costa V., Aprile M., Grimaldi V., Maiello C., Esposito R., Soricelli A., Colantuoni V., Donatelli F., Ciccodicola A. (2017). Heart failure: Pilot transcriptomic analysis of cardiac tissue by RNA-sequencing. Cardiol. J..

[96] Rolfo C., Cardona A.F., Cristofanilli M., Paz-Ares L., Diaz Mochon J.J., Duran I., Raez L.E., Russo A., Lorente J.A., Malapelle U. (2020). Challenges and opportunities of cfDNA analysis implementation in clinical practice: Perspective of the International Society of Liquid Biopsy (ISLB). Crit. Rev. Oncol./Hematol..

[97] Diaz L.A., Bardelli A. (2014). Liquid biopsies: Genotyping circulating tumor DNA. J. Clin. Oncol..

[98] Fleischhacker M., Schmidt B. (2007). Circulating nucleic acids (CNAs) and cancer-a survey. Biochim. Biophys. Acta.

[99] Marsman G., Zeerleder S., Luken B.M. (2016). Extracellular histones, cell-free DNA, or nucleosomes: Differences in immunostimulation. Cell Death Dis..

[100] Yokokawa T., Misaka T., Kimishima Y., Shimizu T., Kaneshiro T., Takeishi Y. (2020). Clinical significance of circulating cardiomyocyte-specific cell-free DNA in patients with heart failure: A proof-of-concept study. Can. J. Cardiol..

[101] Zemmour H., Planer D., Magenheim J., Moss J., Neiman D., Gilon D., Korach A., Glaser B., Shemer R., Landesberg G. (2018). Non-invasive detection of human cardiomyocyte death using methylation patterns of circulating DNA. Nat. Commun..

[102] Krychtiuk K.A., Wurm R., Ruhittel S., Lenz M., Huber K., Wojta J., Heinz G., Hülsmann M., Speidl W.S. (2020). Release of mitochondrial DNA is associated with mortality in severe acute heart failure. Eur. Heart J. Acute Cardiovasc. Care.

[103] Zaravinos A., Tzoras S., Apostolakis S., Lazaridis K., Spandidos D.A. (2011). Levosimendan reduces plasma cell-free DNA levels in patients with ischemic cardiomyopathy. J. Thromb. Thrombolysis.

[104] Berezina T., Berezin O.O., Lichtenauer M., Berezin A.E. (2024). Circulating cell-free nuclear DNA predicted an improvement of systolic left ventricular function in individuals with chronic heart failure with reduced ejection fraction. Cardiogenetics.

[105] Salzano A., Israr M.Z., Garcia D.F., Middleton L., D’Assante R., Marra A.M., Arcopinto M., Yazaki Y., Bernieh D., Cassambai S. (2020). Circulating cell-free DNA levels are associated with adverse outcomes in heart failure: Testing liquid biopsy in heart failure. Eur. J. Prev. Cardiol..

[106] Zhang Q., He X., Ling J., Xiang Q., Li M., Zhao H., Fu Q., Tang Y., He J., Fan W. (2022). Association between circulating cell-free DNA level at admission and the risk of heart failure incidence in acute myocardial infarction patients. DNA Cell Biol..

[107] Sharma S., Artner T., Preissner K.T., Lang I.M. (2024). Nucleic acid liquid biopsies in cardiovascular disease: Cell-free RNA liquid biopsies in cardiovascular disease. Atherosclerosis.

[108] Lewandowski P., Goławski M., Baron M., Reichman-Warmusz E., Wojnicz R. (2022). A systematic review of miRNA and cfDNA as potential biomarkers for liquid biopsy in myocarditis and inflammatory dilated cardiomyopathy. Biomolecules.

[109] Shang R., Lee S., Senavirathne G., Lai E.C. (2023). microRNAs in action: Biogenesis, function and regulation. Nat. Rev. Genet..

[110] Fabian M.R., Sundermeier T.R., Sonenberg N. (2010). Understanding how miRNAs post-transcriptionally regulate gene expression. Prog. Mol. Subcell. Biol..

[111] Bartel D.P. (2018). Metazoan MicroRNAs. Cell.

[112] Ha M., Kim V.N. (2014). Regulation of microRNA biogenesis. Nat. Rev. Mol. Cell. Biol..

[113] Denli A.M., Tops B.B., Plasterk R.H., Ketting R.F., Hannon G.J. (2004). Processing of primary microRNAs by the microprocessor complex. Nature.

[114] Czech B., Hannon G.J. (2011). Small RNA sorting: Matchmaking for argonautes. Nat. Rev. Genet..

[115] Ruby J.G., Jan C.H., Bartel D.P. (2007). Intronic microRNA precursors that bypass Drosha processing. Nature.

[116] Yang J.S., Maurin T., Robine N., Rasmussen K.D., Jeffrey K.L., Chandwani R., Papapetrou E.P., Sadelain M., O’Carroll D., Lai E.C. (2010). Conserved vertebrate mir-451 provides a platform for Dicer-independent, Ago2-mediated microRNA biogenesis. Proc. Natl. Acad. Sci. USA.

[117] Chong M.M., Zhang G., Cheloufi S., Neubert T.A., Hannon G.J., Littman D.R. (2010). Canonical and alternate functions of the microRNA biogenesis machinery. Genes Dev..

[118] Mitchell P.S., Parkin R.K., Kroh E.M., Fritz B.R., Wyman S.K., Pogosova-Agadjanyan E.L., Peterson A., Noteboom J., O’Briant K.C., Allen A. (2008). Circulating microRNAs as stable blood-based markers for cancer detection. Proc. Natl. Acad. Sci. USA.

[119] Mori M.A., Ludwig R.G., Garcia-Martin R., Brandão B.B., Kahn C.R. (2019). Extracellular miRNAs: From biomarkers to mediators of physiology and disease. Cell. Metab..

[120] Gallo A., Tandon M., Alevizos I., Illei G.G. (2012). The majority of microRNAs detectable in serum and saliva is concentrated in exosomes. PLoS ONE.

[121] Arroyo J.D., Chevillet J.R., Kroh E.M., Ruf I.K., Pritchard C.C., Gibson D.F., Mitchell P.S., Bennett C.F., Pogosova-Agadjanyan E.L., Stirewalt D.L. (2011). Argonaute2 complexes carry a population of circulating microRNAs independent of vesicles in human plasma. Proc. Natl. Acad. Sci. USA.

[122] Zhao C., Sun X., Li L. (2019). Biogenesis and function of extracellular miRNAs. ExRNA.

[123] Vickers K.C., Palmisano B.T., Shoucri B.M., Shamburek R.D., Remaley A.T. (2011). MicroRNAs are transported in plasma and delivered to recipient cells by high-density lipoproteins. Nat. Cell. Biol..

[124] Zhang Y., Liu D., Chen X., Li J., Li L., Bian Z., Sun F., Lu J., Yin Y., Cai X. (2010). Secreted monocytic miR-150 enhances targeted endothelial cell migration. Mol. Cell..

[125] Iftikhar H., Carney G.E. (2016). Evidence and potential in vivo functions for biofluid miRNAs: From expression profiling to functional testing: Potential roles of extracellular miRNAs as indicators of physiological change and as agents of intercellular information exchange. Bioessays.

[126] Creemers E.E., Tijsen A.J., Pinto Y.M., van Rooij E. (2012). Circulating microRNAs. Circ. Res..

[127] Silva D., Carneiro F.D., Almeida K.C., Fernandes-Santos C. (2018). Role of miRNAs on the pathophysiology of cardiovascular diseases. Arq. Bras. Cardiol..

[128] Wang G.K., Zhu J.Q., Zhang J.T., Li Q., Li Y., He J., Qin Y.W., Jing Q. (2010). Circulating microRNA: A novel potential biomarker for early diagnosis of acute myocardial infarction in humans. Eur. Heart J..

[129] Fichtlscherer S., De Rosa S., Fox H., Schwietz T., Fischer A., Liebetrau C., Weber M., Hamm C.W., Röxe T., Müller-Ardogan M. (2010). Circulating microRNAs in patients with coronary artery disease. Circ. Res..

[130] Zampetaki A., Kiechl S., Drozdov I., Willeit P., Mayr U., Prokopi M., Mayr A., Weger S., Oberhollenzer F., Bonora E. (2010). Plasma microRNA profiling reveals loss of endothelial miR-126 and other microRNAs in type 2 diabetes. Circ. Res..

[131] Shaheen N., Shaheen A., Diab R.A., Desouki M.T. (2024). MicroRNAs (miRNAs) role in hypertension: Pathogenesis and promising therapeutics. Ann. Med. Surg..

[132] Wu Y., Li Q., Zhang R., Dai X., Chen W., Xing D. (2021). Circulating microRNAs: Biomarkers of disease. Clin. Chim. Acta..

[133] Pozniak T., Shcharbin D., Bryszewska M. (2022). Circulating microRNAs in medicine. Int. J. Mol. Sci..

[134] Budde H., Hassoun R., Mügge A., Kovács Á., Hamdani N. (2022). Current Understanding of molecular pathophysiology of heart failure with preserved ejection fraction. Front. Physiol..

[135] Miner E.C., Miller W.L. (2006). A look between the cardiomyocytes: The extracellular matrix in heart failure. Mayo Clin. Proc..

[136] Tham Y.K., Bernardo B.C., Ooi J.Y., Weeks K.L., McMullen J.R. (2015). Pathophysiology of cardiac hypertrophy and heart failure: Signaling pathways and novel therapeutic targets. Arch. Toxicol..

[137] D’Amato A., Prosperi S., Severino P., Myftari V., Correale M., Perrone Filardi P., Badagliacca R., Fedele F., Vizza C.D., Palazzuoli A. (2024). MicroRNA and heart failure: A novel promising diagnostic and therapeutic tool. J. Clin. Med..

[138] Vegter E.L., van der Meer P., de Windt L.J., Pinto Y.M., Voors A.A. (2016). MicroRNAs in heart failure: From biomarker to target for therapy. Eur. J. Heart Fail..

[139] Kim G.H., Uriel N., Burkhoff D. (2018). Reverse remodelling and myocardial recovery in heart failure. Nat. Rev. Cardiol..

[140] Wehbe N., Nasser S.A., Pintus G., Badran A., Eid A.H., Baydoun E. (2019). MicroRNAs in cardiac hypertrophy. Int. J. Mol. Sci..

[141] Morishima M., Iwata E., Nakada C., Tsukamoto Y., Takanari H., Miyamoto S., Moriyama M., Ono K. (2016). Atrial fibrillation-mediated upregulation of miR-30d regulates myocardial electrical remodeling of the G-protein-gated K(+) channel, IK.ACh. Circ. J..

[142] Balan A.I., Halaţiu V.B., Comșulea E., Mutu C.C., Cozac D.A., Aspru I., Păcurar D., Bănescu C., Perian M., Scridon A. (2025). The diagnostic and predictive potential of miR-328 in atrial fibrillation: Insights from a spontaneously hypertensive rat model. Int. J. Mol. Sci..

[143] Canale P., Borghini A. (2024). Mitochondrial microRNAs: New emerging players in vascular senescence and atherosclerotic cardiovascular disease. Int. J. Mol. Sci..

[144] Song R., Hu X.Q., Zhang L. (2019). Mitochondrial miRNA in cardiovascular function and disease. Cells.

[145] Qaisar R., Karim A., Muhammad T., Shah I., Khan J. (2021). Circulating microRNAs as biomarkers of accelerated sarcopenia in chronic heart failure. Glob. Heart.

[146] Cheng Y., Zhang C. (2010). MicroRNA-21 in cardiovascular disease. J. Cardiovasc. Transl. Res..

[147] Callis T.E., Pandya K., Seok H.Y., Tang R.H., Tatsuguchi M., Huang Z.P., Chen J.F., Deng Z., Gunn B., Shumate J. (2009). MicroRNA-208a is a regulator of cardiac hypertrophy and conduction in mice. J. Clin. Invest..

[148] Wang J., Liew O.W., Richards A.M., Chen Y.T. (2016). Overview of microRNAs in cardiac hypertrophy, fibrosis, and apoptosis. Int. J. Mol. Sci..

[149] Zhu X., Wang H., Liu F., Chen L., Luo W., Su P., Li W., Yu L., Yang X., Cai J. (2013). Identification of micro-RNA networks in end-stage heart failure because of dilated cardiomyopathy. J. Cell Mol. Med..

[150] Abonnenc M., Nabeebaccus A.A., Mayr U., Barallobre-Barreiro J., Dong X., Cuello F., Sur S., Drozdov I., Langley S.R., Lu R. (2013). Extracellular matrix secretion by cardiac fibroblasts. Circ. Res..

[151] Castoldi G., Di Gioia C.R., Bombardi C., Catalucci D., Corradi B., Gualazzi M.G., Leopizzi M., Mancini M., Zerbini G., Condorelli G. (2012). MiR-133a regulates collagen 1A1: Potential role of miR-133a in myocardial fibrosis in angiotensin II-dependent hypertension. J. Cell Physiol..

[152] Chen C., Cai S., Wu M., Wang R., Liu M., Cao G., Dong M., Yiu K.H. (2022). Role of cardiomyocyte-derived exosomal microRNA-146a-5p in macrophage polarization and activation. Dis. Markers.

[153] Lee H.M., Kim T.S., Jo E.K. (2016). MiR-146 and miR-125 in the regulation of innate immunity and inflammation. BMB Rep..

[154] Heymans S., Corsten M.F., Verhesen W., Carai P., van Leeuwen R.E.W., Custers K., Peters T., Hazebroek M., Stöger L., Wijnands E. (2013). Macrophage microRNA-155 promotes cardiac hypertrophy and failure. Circulation.

[155] Hullinger T.G., Montgomery R.L., Seto A.G., Dickinson B.A., Semus H.M., Lynch J.M., Dalby C.M., Robinson K., Stack C., Latimer P.A. (2012). Inhibition of miR-15 protects against cardiac ischemic injury. Circ. Res..

[156] Pan J., Zhou L., Lin C., Xue W., Chen P., Lin J. (2022). MicroRNA-34a promotes ischemia-induced cardiomyocytes apoptosis through targeting notch1. Evid. Based Complement. Alternat. Med..

[157] Chen C., Jia K.Y., Zhang H.L., Fu J. (2016). MiR-195 enhances cardiomyocyte apoptosis induced by hypoxia/reoxygenation injury via downregulating c-myb. Eur. Rev. Med. Pharmacol. Sci..

[158] Hu S., Huang M., Li Z., Jia F., Ghosh Z., Lijkwan M.A., Fasanaro P., Sun N., Wang X., Martelli F. (2010). MicroRNA-210 as a novel therapy for treatment of ischemic heart disease. Circulation.

[159] Wang S., Aurora A.B., Johnson B.A., Qi X., McAnally J., Hill J.A., Richardson J.A., Bassel-Duby R., Olson E.N. (2008). The endothelial-specific microRNA miR-126 governs vascular integrity and angiogenesis. Dev. Cell..

[160] Behnes M., Brueckmann M., Lang S., Weiß C., Ahmad-Nejad P., Neumaier M., Borggrefe M., Hoffmann U. (2014). Connective tissue growth factor (CTGF/CCN2): Diagnostic and prognostic value in acute heart failure. Clin. Res. Cardiol..

[161] Zhu L., Li C., Liu Q., Xu W., Zhou X. (2019). Molecular biomarkers in cardiac hypertrophy. J. Cell. Mol. Med..

[162] Wang J., Yang X. (2012). The function of miRNA in cardiac hypertrophy. Cell. Mol. Life Sci..

[163] van Rooij E., Sutherland L.B., Qi X., Richardson J.A., Hill J., Olson E.N. (2007). Control of stress-dependent cardiac growth and gene expression by a microRNA. Science.

[164] Tatsuguchi M., Seok H.Y., Callis T.E., Thomson J.M., Chen J.F., Newman M., Rojas M., Hammond S.M., Wang D.Z. (2007). Expression of microRNAs is dynamically regulated during cardiomyocyte hypertrophy. J. Mol. Cell. Cardiol..

[165] van Rooij E., Quiat D., Johnson B.A., Sutherland L.B., Qi X., Richardson J.A., Kelm R.J., Olson E.N. (2009). A family of microRNAs encoded by myosin genes governs myosin expression and muscle performance. Dev. Cell..

[166] Huang X.H., Li J.L., Li X.Y., Wang S.X., Jiao Z.H., Li S.Q., Liu J., Ding J. (2021). miR-208a in cardiac hypertrophy and remodeling. Front. Cardiovasc. Med..

[167] Xuan L., Zhu Y., Liu Y., Yang H., Wang S., Li Q., Yang C., Jiao L., Zhang Y., Yang B. (2020). Up-regulation of miR-195 contributes to cardiac hypertrophy-induced arrhythmia by targeting calcium and potassium channels. J. Cell. Mol. Med..

[168] van Rooij E., Sutherland L.B., Liu N., Williams A.H., McAnally J., Gerard R.D., Richardson J.A., Olson E.N. (2006). A signature pattern of stress-responsive microRNAs that can evoke cardiac hypertrophy and heart failure. Proc. Natl. Acad. Sci. USA.

[169] Dirkx E., Gladka M.M., Philippen L.E., Armand A.S., Kinet V., Leptidis S., El Azzouzi H., Salic K., Bourajjaj M., da Silva G.J. (2013). Nfat and miR-25 cooperate to reactivate the transcription factor Hand2 in heart failure. Nat. Cell. Biol..

[170] Raso A., Dirkx E., Sampaio-Pinto V., El Azzouzi H., Cubero R.J., Sorensen D.W., Ottaviani L., Olieslagers S., Huibers M.M., de Weger R. (2021). A microRNA program regulates the balance between cardiomyocyte hyperplasia and hypertrophy and stimulates cardiac regeneration. Nat. Commun..

[171] Ivan M., Huang X. (2014). miR-210: Fine-tuning the hypoxic response. Adv. Exp. Med. Biol..

[172] Chistiakov D.A., Orekhov A.N., Bobryshev Y.V. (2016). Cardiac-specific miRNA in cardiogenesis, heart function, and cardiac pathology (with focus on myocardial infarction). J. Mol. Cell. Cardiol..

[173] Li N., Zhou H., Tang Q. (2018). miR-133: A suppressor of cardiac remodeling?. Front. Pharmacol..

[174] Li L., Zhao Q., Kong W. (2018). Extracellular matrix remodeling and cardiac fibrosis. Matrix Biol..

[175] Fan D., Takawale A., Lee J., Kassiri Z. (2012). Cardiac fibroblasts, fibrosis and extracellular matrix remodeling in heart disease. Fibrogenes. Tissue Repair..

[176] Hashimoto H., Wang Z., Garry G.A., Malladi V.S., Botten G.A., Ye W., Zhou H., Osterwalder M., Dickel D.E., Visel A. (2019). Cardiac reprogramming factors synergistically activate genome-wide cardiogenic stage-specific enhancers. Cell. Stem. Cell..

[177] Paoletti C., Divieto C., Tarricone G., Di Meglio F., Nurzynska D., Chiono V. (2020). MicroRNA-mediated direct reprogramming of human adult fibroblasts toward cardiac phenotype. Front. Bioeng. Biotechnol..

[178] Thum T., Gross C., Fiedler J., Fischer T., Kissler S., Bussen M., Galuppo P., Just S., Rottbauer W., Frantz S. (2008). MicroRNA-21 contributes to myocardial disease by stimulating MAP kinase signalling in fibroblasts. Nature.

[179] Patrick D.M., Montgomery R.L., Qi X., Obad S., Kauppinen S., Hill J.A., van Rooij E., Olson E.N. (2010). Stress-dependent cardiac remodeling occurs in the absence of microRNA-21 in mice. J. Clin. Investig..

[180] van Rooij E., Sutherland L.B., Thatcher J.E., DiMaio J.M., Naseem R.H., Marshall W.S., Hill J.A., Olson E.N. (2008). Dysregulation of microRNAs after myocardial infarction reveals a role of miR-29 in cardiac fibrosis. Proc. Natl. Acad. Sci. USA.

[181] Sun M., Yu H., Zhang Y., Li Z., Gao W. (2015). MicroRNA-214 mediates isoproterenol-induced proliferation and collagen synthesis in cardiac fibroblasts. Sci. Rep..

[182] Yuan X., Pan J., Wen L., Gong B., Li J., Gao H., Tan W., Liang S., Zhang H., Wang X. (2019). MiR-144-3p enhances cardiac fibrosis after myocardial infarction by targeting PTEN. Front. Cell. Dev. Biol..

[183] Chang W., Xiao D., Fang X., Wang J. (2024). Oxidative modification of miR-30c promotes cardiac fibroblast proliferation via CDKN2C mismatch. Sci. Rep..

[184] Zhang Z., Li X., Zhuang J., Ding Q., Zheng H., Ma T., Meng Q., Gao L. (2024). miR-590-3p overexpression improves the efficacy of hiPSC-CMs for myocardial repair. JACC Basic Transl. Sci..

[185] Yibulayin K., Abulizi M. (2024). The function of miRNAs in the immune system’s inflammatory reaction to heart failure. Front. Cardiovasc. Med..

[186] van de Vrie M., Heymans S., Schroen B. (2011). MicroRNA involvement in immune activation during heart failure. Cardiovasc. Drugs Ther..

[187] Roy S., Khanna S., Hussain S.R., Biswas S., Azad A., Rink C., Gnyawali S., Shilo S., Nuovo G.J., Sen C.K. (2009). MicroRNA expression in response to murine myocardial infarction: miR-21 regulates fibroblast metalloprotease-2 via phosphatase and tensin homologue. Cardiovasc. Res..

[188] Li W., Li Y., Jiang F., Liu H. (2022). Correlation between serum levels of microRNA-21 and inflammatory factors in patients with chronic heart failure. Medicine.

[189] O’Connell R.M., Taganov K.D., Boldin M.P., Cheng G., Baltimore D. (2007). MicroRNA-155 is induced during the macrophage inflammatory response. Proc. Natl. Acad. Sci. USA.

[190] Eisenhardt S.U., Weiss J.B., Smolka C., Maxeiner J., Pankratz F., Bemtgen X., Kustermann M., Thiele J.R., Schmidt Y., Bjoern Stark G. (2015). MicroRNA-155 aggravates ischemia-reperfusion injury by modulation of inflammatory cell recruitment and the respiratory oxidative burst. Basic. Res. Cardiol..

[191] Beg F., Wang R., Saeed Z., Devaraj S., Masoor K., Nakshatri H. (2017). Inflammation-associated microRNA changes in circulating exosomes of heart failure patients. BMC Res. Notes.

[192] Fei Y., Chaulagain A., Wang T., Chen Y., Liu J., Yi M., Wang Y., Huang Y., Lin L., Chen S. (2020). MiR-146a down-regulates inflammatory response by targeting TLR3 and TRAF6 in *Coxsackievirus* B infection. RNA.

[193] Siegel P.M., Schmich J., Barinov G., Bojti I., Vedecnik C., Simanjuntak N.R., Bode C., Moser M., Peter K., Diehl P. (2020). Cardiomyocyte microvesicles: Proinflammatory mediators after myocardial ischemia?. J. Thromb. Thrombolysis.

[194] Guo D., Yan J., Yang Z., Chen M., Zhong W., Yuan X., Yu S. (2024). The immune regulatory role of exosomal miRNAs and their clinical application potential in heart failure. Front. Immunol..

[195] Chen C., Zong M., Lu Y., Guo Y., Lv H., Xie L., Fu Z., Cheng Y., Si Y., Ye B. (2020). Differentially expressed lnc-NOS2P3-miR-939-5p axis in chronic heart failure inhibits myocardial and endothelial cells apoptosis via iNOS/TNFα pathway. J. Cell. Mol. Med..

[196] de Couto G., Gallet R., Cambier L., Jaghatspanyan E., Makkar N., Dawkins J.F., Berman B.P., Marbán E. (2017). Exosomal microRNA transfer into macrophages mediates cellular postconditioning. Circulation.

[197] de Couto G., Jaghatspanyan E., DeBerge M., Liu W., Luther K., Wang Y., Tang J., Thorp E.B., Marbán E. (2019). Mechanism of enhanced MerTK-dependent macrophage efferocytosis by extracellular vesicles. Arterioscler. Thromb. Vasc. Biol..

[198] Ma Y., Su M., Qian W., Xuan Y., Chen T., Zhou R., Jiang T. (2023). CXCR4-overexpressed exosomes from cardiosphere-derived cells attenuate myocardial ischemia/reperfusion injury by transferring miRNA to macrophages and regulating macrophage polarization. Cell. Mol. Biol..

[199] Jansen F., Yang X., Baumann K., Przybilla D., Schmitz T., Flender A., Paul K., Alhusseiny A., Nickenig G., Werner N. (2015). Endothelial microparticles reduce ICAM-1 expression in a microRNA-222-dependent mechanism. J. Cell. Mol. Med..

[200] Zhang X., Wang X., Fan M., Tu F., Yang K., Ha T., Liu L., Kalbfleisch J., Williams D., Li C. (2020). Endothelial HSPA12B exerts protection against sepsis-induced severe cardiomyopathy via suppression of adhesion molecule expression by miR-126. Front. Immunol..

[201] Wu X.Y., Fan W.D., Fang R., Wu G.F. (2014). Regulation of microRNA-155 in endothelial inflammation by targeting nuclear factor (NF)-κB P65. J. Cell. Biochem..

[202] Gilyazova I., Asadullina D., Kagirova E., Sikka R., Mustafin A., Ivanova E., Bakhtiyarova K., Gilyazova G., Gupta S., Khusnutdinova E. (2023). MiRNA-146a-a key player in immunity and diseases. Int. J. Mol. Sci..

[203] Cheng H.S., Sivachandran N., Lau A., Boudreau E., Zhao J.L., Baltimore D., Delgado-Olguin P., Cybulsky M.I., Fish J.E. (2013). MicroRNA-146 represses endothelial activation by inhibiting pro-inflammatory pathways. EMBO Mol. Med..

[204] Feng L.L., Xin W.N., Tian X.L. (2019). MALAT1 modulates miR-146’s protection of microvascular endothelial cells against LPS-induced NF-κB activation and inflammatory injury. Innate Immun..

[205] Zhang Z., Zou Y., Song C., Cao K., Cai K., Chen S., Wu Y., Geng D., Sun G., Zhang N. (2024). Advances in the study of exosomes in cardiovascular diseases. J. Adv. Res..

[206] Kang P.M., Izumo S. (2000). Apoptosis and heart failure. Circ. Res..

[207] Zhang M., Liu Q., Meng H., Duan H., Liu X., Wu J., Gao F., Wang S., Tan R., Yuan J. (2024). Ischemia-reperfusion injury: Molecular mechanisms and therapeutic targets. Sig. Transduct. Target. Ther..

[208] Roiz-Valle D., Caravia X.M., López-Otín C. (2023). Mechanisms of mitochondrial microRNA regulation in cardiovascular diseases. Mech. Ageing Dev..

[209] Xue P., Liu Y., Wang H., Huang J., Luo M. (2023). miRNA-103-3p-Hlf regulates apoptosis and autophagy by targeting hepatic leukaemia factor in heart failure. ESC Heart Fail..

[210] Boon R.A., Iekushi K., Lechner S., Seeger T., Fischer A., Heydt S., Kaluza D., Tréguer K., Carmona G., Bonauer A. (2013). MicroRNA-34a regulates cardiac ageing and function. Nature.

[211] Zheng M., Wang M. (2021). A narrative review of the roles of the miR-15/107 family in heart disease: Lessons and prospects for heart disease. Ann. Transl. Med..

[212] Lv X.B., Niu Q.H., Zhang M., Feng L., Feng J. (2021). Critical functions of microRNA-30a-5p-E2F3 in cardiomyocyte apoptosis induced by hypoxia/reoxygenation. Kaohsiung. J. Med. Sci..

[213] Xing X., Guo S., Zhang G., Liu Y., Bi S., Wang X., Lu Q. (2020). miR-26a-5p protects against myocardial ischemia/reperfusion injury by regulating the PTEN/PI3K/AKT signaling pathway. Braz. J. Med. Biol. Res..

[214] Qian L., Van Laake L.W., Huang Y., Liu S., Wendland M.F., Srivastava D. (2011). miR-24 inhibits apoptosis and represses Bim in mouse cardiomyocytes. J. Exp. Med..

[215] Wang X., Ha T., Hu Y., Lu C., Liu L., Zhang X., Kao R., Kalbfleisch J., Williams D., Li C. (2016). MicroRNA-214 protects against hypoxia/reoxygenation induced cell damage and myocardial ischemia/reperfusion injury via suppression of PTEN and Bim1 expression. Oncotarget..

[216] Cimmino A., Calin G.A., Fabbri M., Iorio M.V., Ferracin M., Shimizu M., Wojcik S.E., Aqeilan R.I., Zupo S., Dono M. (2005). miR-15 and miR-16 induce apoptosis by targeting BCL2. Proc. Natl. Acad. Sci. USA.

[217] Wang J., Feng Q., Liang D., Shi J. (2021). MiRNA-26a inhibits myocardial infarction-induced apoptosis by targeting PTEN via JAK/STAT pathways. Cells Dev..

[218] Grego A., Fernandes C., Fonseca I., Dias-Neto M., Costa R., Leite-Moreira A., Oliveira S.M., Trindade F., Nogueira-Ferreira R. (2025). Endothelial dysfunction in cardiovascular diseases: Mechanisms and in vitro models. Mol. Cell. Biochem..

[219] Landskroner-Eiger S., Moneke I., Sessa W.C. (2013). miRNAs as modulators of angiogenesis. Cold Spring Harb. Perspect. Med..

[220] Sessa F., Salerno M., Esposito M., Cocimano G., Pomara C. (2023). miRNA dysregulation in cardiovascular diseases: Current opinion and future perspectives. Int. J. Mol. Sci..

[221] Masson S., Batkai S., Beermann J., Bär C., Pfanne A., Thum S., Magnoli M., Balconi G., Nicolosi G.L., Tavazzi L. (2018). Circulating microRNA-132 levels improve risk prediction for heart failure hospitalization in patients with chronic heart failure. Eur. J. Heart Fail..

[222] Shen L., Fan G., Yang G., Yang Z., Gui C. (2023). Paracrine effects of mir-210-3p on angiogenesis in hypoxia-treated c-kit-positive cardiac cells. Ann. Med..

[223] Kattih B., Fischer A., Muhly-Reinholz M., Tombor L., Nicin L., Cremer S., Zeiher A.M., John D., Abplanalp W.T., Dimmeler S. (2025). Inhibition of miR-92a normalizes vascular gene expression and prevents diastolic dysfunction in heart failure with preserved ejection fraction. J. Mol. Cell. Cardiol..

[224] Bonauer A., Carmona G., Iwasaki M., Mione M., Koyanagi M., Fischer A., Burchfield J., Fox H., Doebele C., Ohtani K. (2009). MicroRNA-92a controls angiogenesis and functional recovery of ischemic tissues in mice. Science.

[225] Zhang C., He J., Xiong D., Mei Y., Zhu Y., Deng P., Duan Y. (2025). Effect of miR-1285-3p as a diagnostic biomarker for chronic heart failure on vascular endothelial cells: (Effect of miR-1285-3p as a biomarker for CHF on HUVECs). J. Cardiothorac. Surg..

[226] Magenta A., Ciarapica R., Capogrossi M.C. (2017). The emerging role of miR-200 family in cardiovascular diseases. Circ. Res..

[227] Liu X., Tong Z., Chen K., Hu X., Jin H., Hou M. (2018). The role of miRNA-132 against apoptosis and oxidative stress in heart failure. Biomed. Res. Int..

[228] Tijsen A.J., Creemers E.E., Moerland P.D., de Windt L.J., van der Wal A.C., Kok W.E., Pinto Y.M. (2010). MiR423-5p as a circulating biomarker for heart failure. Circ. Res..

[229] Corsten M.F., Dennert R., Jochems S., Kuznetsova T., Devaux Y., Hofstra L., Wagner D.R., Staessen J.A., Heymans S., Schroen B. (2010). Circulating microRNA-208b and microRNA-499 reflect myocardial damage in cardiovascular disease. Circ. Cardiovasc. Genet..

[230] Koyama S., Kuragaichi T., Sato Y., Kuwabara Y., Usami S., Horie T., Baba O., Hakuno D., Nakashima Y., Nishino T. (2017). Dynamic changes of serum microRNA-122-5p through therapeutic courses indicates amelioration of acute liver injury accompanied by acute cardiac decompensation. ESC Heart Fail..

[231] Goren Y., Kushnir M., Zafrir B., Tabak S., Lewis B.S., Amir O. (2012). Serum levels of microRNAs in patients with heart failure. Eur. J. Heart Fail..

[232] Blanco R.R., Austin H., Vest R.N., Valadri R., Li W., Lassegue B., Song Q., London B., Dudley S.C., Bloom H.L. (2012). Angiotensin receptor type 1 single nucleotide polymorphism 1166A/C is associated with malignant arrhythmias and altered circulating miR-155 levels in patients with chronic heart failure. J. Card. Fail..

[233] Ali Sheikh M.S., Salma U., Zhang B., Chen J., Zhuang J., Ping Z. (2016). Diagnostic, prognostic, and therapeutic value of circulating miRNAs in heart failure patients associated with oxidative stress. Oxid. Med. Cell. Longev..

[234] Watson C.J., Gupta S.K., O’Connell E., Thum S., Glezeva N., Fendrich J., Gallagher J., Ledwidge M., Grote-Levi L., McDonald K. (2015). MicroRNA signatures differentiate preserved from reduced ejection fraction heart failure. Eur. J. Heart Fail..

[235] Voellenkle C., van Rooij J., Cappuzzello C., Greco S., Arcelli D., Di Vito L., Melillo G., Rigolini R., Costa E., Crea F. (2010). MicroRNA signatures in peripheral blood mononuclear cells of chronic heart failure patients. Physiol. Genomics.

[236] Danowski N., Manthey I., Jakob H.G., Siffert W., Peters J., Frey U.H. (2013). Decreased expression of miR-133a but not of miR-1 is associated with signs of heart failure in patients undergoing coronary bypass surgery. Cardiology.

[237] Matkovich S.J., Van Booven D.J., Youker K.A., Torre-Amione G., Diwan A., Eschenbacher W.H., Dorn L.E., Watson M.A., Margulies K.B., Dorn G.W. (2009). Reciprocal regulation of myocardial microRNAs and messenger RNA in human cardiomyopathy and reversal of the microRNA signature by biomechanical support. Circulation.

[238] Wei X.J., Han M., Yang F.Y., Wei G.C., Liang Z.G., Yao H., Ji C.W., Xie R.S., Gong C.L., Tian Y. (2015). Biological significance of miR-126 expression in atrial fibrillation and heart failure. Braz. J. Med. Biol. Res..

[239] Zhao D.S., Chen Y., Jiang H., Lu J.P., Zhang G., Geng J., Zhang Q., Shen J.H., Zhou X., Zhu W. (2013). Serum miR-210 and miR-30a expressions tend to revert to fetal levels in Chinese adult patients with chronic heart failure. Cardiovasc. Pathol..

[240] Parvan R., Hosseinpour M., Moradi Y., Devaux Y., Cataliotti A., da Silva G.J.J. (2022). Diagnostic performance of microRNAs in the detection of heart failure with reduced or preserved ejection fraction: A systematic review and meta-analysis. Eur. J. Heart Fail..

[241] Kuai Z., Ma Y., Gao W., Zhang X., Wang X., Ye Y., Zhang X., Yuan J. (2024). Potential diagnostic value of circulating miRNAs in HFrEF and bioinformatics analysis. Heliyon.

[242] Chen F., Yang J., Li Y., Wang H. (2018). Circulating microRNAs as novel biomarkers for heart failure. Hell. J. Cardiol..

[243] Seronde M.F., Vausort M., Gayat E., Goretti E., Ng L.L., Squire I.B., Vodovar N., Sadoune M., Samuel J.L., Thum T. (2015). Circulating microRNAs and outcome in patients with acute heart failure. PLoS ONE.

[244] Xiao J., Gao R., Bei Y., Zhou Q., Zhou Y., Zhang H., Jin M., Wei S., Wang K., Xu X. (2017). Circulating miR-30d predicts survival in patients with acute heart failure. Cell. Physiol. Biochem..

[245] De Rosa S., Eposito F., Carella C., Strangio A., Ammirati G., Sabatino J., Abbate F.G., Iaconetti C., Liguori V., Pergola V. (2018). Transcoronary concentration gradients of circulating microRNAs in heart failure. Eur. J. Heart Fail..

[246] Sucharov C.C., Kao D.P., Port J.D., Karimpour-Fard A., Quaife R.A., Minobe W., Nunley K., Lowes B.D., Gilbert E.M., Bristow M.R. (2017). Myocardial microRNAs associated with reverse remodeling in human heart failure. JCI Insight.

[247] Marfella R., Di Filippo C., Potenza N., Sardu C., Rizzo M.R., Siniscalchi M., Musacchio E., Barbieri M., Mauro C., Mosca N. (2013). Circulating microRNA changes in heart failure patients treated with cardiac resynchronization therapy: Responders vs. non-responders. Eur. J. Heart Fail..

[248] Melman Y.F., Shah R., Danielson K., Xiao J., Simonson B., Barth A., Chakir K., Lewis G.D., Lavender Z., Truong Q.A. (2015). Circulating MicroRNA-30d is associated with response to cardiac resynchronization therapy in heart failure and regulates cardiomyocyte apoptosis: A translational pilot study. Circulation.

[249] Melak T., Baynes H.W. (2019). Circulating microRNAs as possible biomarkers for coronary artery disease: A narrative review. EJIFCC.

[250] Tanase D.M., Gosav E.M., Ouatu A., Badescu M.C., Dima N., Ganceanu-Rusu A.R., Popescu D., Floria M., Rezus E., Rezus C. (2021). Current knowledge of microRNAs (miRNAs) in acute coronary syndrome (ACS): ST-elevation myocardial infarction (STEMI). Life.

[251] Wu Y., Pan N., An Y., Xu M., Tan L., Zhang L. (2021). Diagnostic and prognostic biomarkers for myocardial infarction. Front. Cardiovasc. Med..

[252] Wang X., Lu Y., Zhao R., Zhu B., Liu J., Yue Q., Wu R., Han S., Gao Y., Chen J. (2024). Global surveillance of circulating microRNA for diagnostic and prognostic assessment of acute myocardial infarction based on the plasma small RNA sequencing. Biomark. Res..

[253] Scărlătescu A.I., Micheu M.M., Popa-Fotea N.-M., Dorobanțu M. (2021). MicroRNAs in acute ST elevation myocardial infarction—A new tool for diagnosis and prognosis: Therapeutic implications. Int. J. Mol. Sci..

[254] He F., Guan W. (2025). The role of miR-21 as a biomarker and therapeutic target in cardiovascular disease. Clin. Chim. Acta.

[255] Kaur A., Mackin S.T., Schlosser K., Wong F.L., Elharram M., Delles C., Stewart D.J., Dayan N., Landry T., Pilote L. (2019). Systematic review of microRNA biomarkers in acute coronary syndrome and stable coronary artery disease. Cardiovasc. Res..

[256] Kayvanpour E., Gi W.T., Sedaghat-Hamedani F., Lehmann D.H., Frese K.S., Haas J., Tappu R., Samani O.S., Nietsch R., Kahraman M. (2021). microRNA neural networks improve diagnosis of acute coronary syndrome (ACS). J. Mol. Cell. Cardiol..

[257] Cunningham K.S., Veinot J.P., Butany J. (2006). An approach to endomyocardial biopsy interpretation. J. Clin. Pathol..

[258] Duong Van Huyen J.-P., Tible M., Gay A., Guillemain R., Aubert O., Varnous S., Iserin F., Rouvier P., François A., Vernerey D. (2014). MicroRNAs as non-invasive biomarkers of heart transplant rejection. Eur. Heart J..

[259] Wang E., Nie Y., Zhao Q., Wang W., Huang J., Liao Z., Zhang H., Hu S., Zheng Z. (2013). Circulating miRNAs reflect early myocardial injury and recovery after heart transplantation. J. Cardiothorac. Surg..

[260] Sukma Dewi I., Hollander Z., Lam K.K., McManus J.W., Tebbutt S.J., Ng R.T., Keown P.A., McMaster R.W., McManus B.M., Gidlöf O. (2017). Association of serum miR-142-3p and miR-101-3p levels with acute cellular rejection after heart transplantation. PLoS ONE.

[261] Zhou L., Zang G., Zhang G., Wang H., Zhang X., Johnston N., Min W., Luke P., Jevnikar A., Haig A. (2013). MicroRNA and mRNA signatures in ischemia reperfusion Injury in heart transplantation. PLoS ONE.

[262] Coutance G., Racapé M., Baudry G., Lécuyer L., Roubille F., Blanchart K., Epailly E., Vermes E., Pattier S., Boignard A. (2023). Validation of the clinical utility of microRNA as noninvasive biomarkers of cardiac allograft rejection: A prospective longitudinal multicenter study. J. Heart Lung Transplant..

[263] Shah P., Bristow M.R., Port J.D. (2017). MicroRNAs in heart failure, cardiac transplantation, and myocardial recovery: Biomarkers with therapeutic potential. Curr. Heart Fail. Rep..

[264] Zannad F., Ferreira J.P., Pocock S.J., Anker S.D., Butler J., Filippatos G., Brueckmann M., Ofstad A.P., Pfarr E., Jamal W. (2020). SGLT2 inhibitors in patients with heart failure with reduced ejection fraction: A meta-analysis of the EMPEROR-Reduced and DAPA-HF trials. Lancet.

[265] Sutanto H., Dobrev D., Heijman J. (2021). Angiotensin receptor-neprilysin inhibitor (ARNI) and cardiac arrhythmias. Int. J. Mol. Sci..

[266] Mone P., Lombardi A., Kansakar U., Varzideh F., Jankauskas S.S., Pansini A., Marzocco S., De Gennaro S., Famiglietti M., Macina G. (2023). Empagliflozin improves the microRNA signature of endothelial dysfunction in patients with heart failure with preserved ejection fraction and diabetes. J. Pharmacol. Exp. Ther..

[267] Kiyak-Kirmaci H., Hazar-Yavuz A.N., Polat E.B., Alsaadoni H., Cilingir-Kaya O.T., Aktas H.S., Elcioglu H.K. (2025). Effects of empagliflozin and dapagliflozin, SGLT2 inhibitors, on miRNA expressions in diabetes-related cardiovascular damage in rats. J. Diabetes Complicat..

[268] Brioschi M., D’Alessandra Y., Mapelli M., Mattavelli I., Salvioni E., Eligini S., Mallia A., Ricci V., Gianazza E., Ghilardi S. (2023). Impact of Sacubitril/Valsartan on circulating microRNA in patients with heart failure. Biomedicines.

[269] Liu B., Wang B., Zhang X., Lock R., Nash T., Vunjak-Novakovic G. (2021). Cell type-specific microRNA therapies for myocardial infarction. Sci. Transl. Med..

[270] Lima J.F., Cerqueira L., Figueiredo C., Oliveira C., Azevedo N.F. (2018). Anti-miRNA oligonucleotides: A comprehensive guide for design. RNA Biol..

[271] Reid G., Williams M., Cheng Y.Y., Sarun K., Winata P., Kirschner M.B., Mugridge N., Weiss J., Molloy M., Brahmbhatt H. (2025). Therapeutic potential of synthetic microRNA mimics based on the miR-15/107 consensus sequence. Cancer Gene Ther..

[272] Bauersachs J., Solomon S.D., Anker S.D., Antorrena-Miranda I., Batkai S., Viereck J., Rump S., Filippatos G., Granzer U., Ponikowski P. (2024). Efficacy and safety of CDR132L in patients with reduced left ventricular ejection fraction after myocardial infarction: Rationale and design of the HF-REVERT trial. Eur. J. Heart Fail..

[273] Bellera N., Barba I., Rodriguez-Sinovas A., Ferret E., Asín M.A., Gonzalez-Alujas M.T., Pérez-Rodon J., Esteves M., Fonseca C., Toran N. (2014). Single intracoronary injection of encapsulated antagomir-92a promotes angiogenesis and prevents adverse infarct remodeling. J. Am. Heart Assoc..

[274] Nalbant E., Akkaya-Ulum Y.Z. (2024). Exploring regulatory mechanisms on miRNAs and their implications in inflammation-related diseases. Clin. Exp. Med..

[275] Felekkis K., Papaneophytou C. (2024). The circulating biomarkers league: Combining miRNAs with cell-free DNAs and proteins. Int. J. Mol. Sci..

[276] Spahillari A., Jackson L., Varrias D., Michelhaugh S.A., Januzzi J.L., Shahideh B., Daghfal D., Valkov N., Murtagh G., Das S. (2024). MicroRNAs are associated with cardiac biomarkers, cardiac structure and function and incident outcomes in heart failure. ESC Heart Fail..

[277] Condrat C.E., Thompson D.C., Barbu M.G., Bugnar O.L., Boboc A., Cretoiu D., Suciu N., Cretoiu S.M., Voinea S.C. (2020). miRNAs as biomarkers in disease: Latest findings regarding their role in diagnosis and prognosis. Cells.

[278] Godsey J.H., Silvestro A., Barrett J.C., Bramlett K., Chudova D., Deras I., Dickey J., Hicks J., Johann D.J., Leary R. (2020). Generic protocols for the analytical validation of next-generation sequencing-based ctDNA assays: A Joint consensus recommendation of the BloodPAC’s analytical variables working group. Clin. Chem..

[279] Pantel K., Alix-Panabières C., Hofman P., Stoecklein N.H., Lu Y.-J., Lianidou E., Giacomini P., Koch C., de Jager V., Deans Z.C. (2025). Fostering the implementation of liquid biopsy in clinical practice: Meeting report 2024 of the European Liquid Biopsy Society (ELBS). J. Exp. Clin. Cancer Res..

[280] Bustin S.A., Ruijter J.M., van den Hoff M.J.B., Kubista M., Pfaffl M.W., Shipley G.L., Tran N., Rödiger S., Untergasser A., Mueller R. (2025). MIQE 2.0: Revision of the minimum information for publication of quantitative real-time PCR experiments guidelines. Clin. Chem..

[281] Endo K., Naito Y., Ji X., Nakanishi M., Noguchi T., Goto Y., Nonogi H., Ma X., Weng H., Hirokawa G. (2013). MicroRNA 210 as a biomarker for congestive heart failure. Biol. Pharm. Bull..

[282] Ellis K.L., Cameron V.A., Troughton R.W., Frampton C.M., Ellmers L.J., Richards A.M. (2013). Circulating microRNAs as candidate markers to distinguish heart failure in breathless patients. Eur. J. Heart Fail..

[283] Wong L.L., Armugam A., Sepramaniam S., Karolina D.S., Lim K.Y., Lim J.Y., Chong J.P.C., Ng J.Y.X., Chen Y.-T., Chan M.M.Y. (2015). Circulating microRNAs in heart failure with reduced and preserved left ventricular ejection fraction. Eur. J. Heart Fail..

[284] Yan H., Ma F., Zhang Y., Wang C., Qiu D., Zhou K., Hua Y., Li Y. (2017). miRNAs as biomarkers for diagnosis of heart failure: A systematic review and meta-analysis. Medicine.

[285] Wong L.L., Zou R., Zhou L., Lim J.Y., Phua D.C.Y., Liu C., Chong J.P.C., Ng J.Y.X., Liew O.W., Chan S.P. (2019). Combining circulating microRNA and NT-proBNP to detect and categorize heart failure subtypes. J. Am. Coll. Cardiol..

[286] Al-Hayali M.A., Sozer V., Durmus S., Erdenen F., Altunoglu E., Gelisgen R., Atukeren P., Atak P.G., Uzun H. (2019). Clinical value of circulating microribonucleic acids miR-1 and miR-21 in evaluating the diagnosis of acute heart failure in asymptomatic type 2 diabetic patients. Biomolecules.

[287] Tomaniak M., Sygitowicz G., Błaszczyk O., Kołtowski Ł., Puchta D., Malesa K., Kochanowski J., Sitkiewicz D., Filipiak K.J. (2018). miR-1, miR-21, and galectin-3 in hypertensive patients with symptomatic heart failure and left ventricular hypertrophy. Kardiol. Pol..

[288] Han R., Li K., Li L., Zhang L., Zheng H. (2020). Expression of microRNA-214 and galectin-3 in peripheral blood of patients with chronic heart failure and its clinical significance. Exp. Ther. Med..

[289] Zhang Y., Liu Y.J., Liu T., Zhang H., Yang S.J. (2016). Plasma microRNA-21 is a potential diagnostic biomarker of acute myocardial infarction. Eur. Rev. Med. Pharmacol. Sci..

[290] Arul J.C., Raja Beem S.S., Parthasarathy M., Kuppusamy M.K., Rajamani K., Silambanan S. (2025). Association of microRNA-210-3p with NT-proBNP, sST2, and Galectin-3 in heart failure patients with preserved and reduced ejection fraction: A cross-sectional study. PLoS ONE.

[291] Felekkis K., Papaneophytou C. (2020). Challenges in using circulating micro-RNAs as biomarkers for cardiovascular diseases. Int. J. Mol. Sci..

[292] Kaneto C.M., Nascimento J.S., Prado M., Mendonça L.S.O. (2019). Circulating miRNAs as biomarkers in cardiovascular diseases. Eur. Rev. Med. Pharmacol. Sci..

[293] Zendjabil M. (2024). Preanalytical, analytical and postanalytical considerations in circulating microRNAs measurement. Biochem. Med..

[294] Godoy P.M., Barczak A.J., DeHoff P., Srinivasan S., Etheridge A., Galas D., Das S., Erle D.J., Laurent L.C. (2019). Comparison of reproducibility, accuracy, sensitivity, and specificity of miRNA quantification platforms. Cell. Rep..

[295] de Gonzalo-Calvo D., Pérez-Boza J., Curado J., Devaux Y. (2022). Challenges of microRNA-based biomarkers in clinical application for cardiovascular diseases. Clin. Transl. Med..

[296] Ou F.S., Michiels S., Shyr Y., Adjei A.A., Oberg A.L. (2021). Biomarker discovery and validation: Statistical considerations. J. Thorac. Oncol..

[297] Hackler E., Lew J., Gore M.O., Ayers C.R., Atzler D., Khera A., Rohatgi A., Lewis A., Neeland I., Omland T. (2019). Racial differences in cardiovascular biomarkers in the general population. J. Am. Heart Assoc..

[298] Rao R.A., Bhardwaj A., Munagala M., Abraham S., Adig S., Shen A., Hamad E. (2024). Sex differences in circulating biomarkers of heart failure. Curr. Heart Fail. Rep..

[299] Turko R., Hajja A., Magableh A.M., Omer M.H., Shafqat A., Khan M.I., Yaqinuddin A. (2025). The emerging role of miRNAs in biological aging and age-related diseases. Noncoding RNA Res..

[300] Loveridge A. (2025). Mind the gap: Sex-related differences in cardiovascular disease. Nat. Rev. Cardiol..

[301] Emerson J.I., Shi W., Paredes-Larios J., Walker W.G., Hutton J.E., Cristea I.M., Marzluff W.F., Conlon F.L. (2025). X-Chromosome–linked miRNAs regulate sex differences in cardiac physiology. Circ. Res..

[302] Palazzuoli A., Beltrami M. (2021). Are HFpEF and HFmrEF so different? The need to understand distinct phenotypes. Front. Cardiovasc. Med..

[303] Goyal P., Zainul O., Sharma Y., Reich A., Osma P., Lau J.D., Massou E., Turchioe M., Russell D., Creber R.M. (2025). Geriatric vulnerabilities among adults with heart failure with preserved ejection fraction. JACC Adv..

[304] Thorsen S.U., Moseholm K.F., Clausen F.B. (2024). Circulating cell-free DNA and its association with cardiovascular disease: What we know and future perspectives. Curr. Opin. Lipidol..

[305] Polina I.A., Ilatovskaya D.V., DeLeon-Pennell K.Y. (2020). Cell free DNA as a diagnostic and prognostic marker for cardiovascular diseases. Clin. Chim. Acta..

[306] Keup C., Kimmig R., Kasimir-Bauer S. (2022). Multimodality in liquid biopsy: Does a combination uncover insights undetectable in individual blood analytes?. J. Lab. Med..

[307] Bortolini Silveira A., Bidard F.C., Tanguy M.L., Girard E., Trédan O., Dubot C., Jacot W., Goncalves A., Debled M., Levy C. (2021). Multimodal liquid biopsy for early monitoring and outcome prediction of chemotherapy in metastatic breast cancer. NPJ Breast Cancer.

[308] Gokulnath P., Spanos M., Lehmann H.I., Sheng Q., Rodosthenous R., Chaffin M., Varrias D., Chatterjee E., Hutchins E., Li G. (2024). Distinct plasma extracellular vesicle transcriptomes in acute decompensated heart failure subtypes: A liquid biopsy approach. Circulation.

[309] Parvan R., Becker V., Hosseinpour M., Moradi Y., Louch W.E., Cataliotti A., Devaux Y., Frisk M., Silva G.J.J., Athero N.E.T.C.A.C.A. (2025). Prognostic and predictive microRNA panels for heart failure patients with reduced or preserved ejection fraction: A meta-analysis of Kaplan–Meier-based individual patient data. BMC Med..

[310] Parvan R., Rolim N., Gevaert A.B., Cataliotti A., van Craenenbroeck E.M., Adams V., Wisløff U., Silva G.J.J., OptimEx Study Group (2025). Multi-microRNA diagnostic panel for heart failure with preserved ejection fraction in preclinical and clinical settings. ESC Heart Fail..

[311] Khan M.S., Arshad M.S., Greene S.J., Van Spall H.G.C., Pandey A., Vemulapalli S., Perakslis E., Butler J. (2023). Artificial intelligence and heart failure: A state-of-the-art review. Eur. J. Heart Fail..

[312] Saqib M., Perswani P., Muneem A., Mumtaz H., Neha F., Ali S., Tabassum S. (2024). Machine learning in heart failure diagnosis, prediction, and prognosis: Review. Ann. Med. Surg..

[313] Liu H.E., Vuppalapaty M., Wilkerson C., Renier C., Chiu M., Lemaire C., Che J., Matsumoto M., Carroll J., Crouse S. (2020). Detection of EGFR mutations in cfDNA and CTCs, and comparison to tumor tissue in non-small-cell-lung-cancer (NSCLC) patients. Front. Oncol..

